# Photoluminescence control by atomically precise surface metallization of C-centered hexagold(i) clusters using N-heterocyclic carbenes†

**DOI:** 10.1039/d3sc01976d

**Published:** 2023-05-11

**Authors:** Zhen Lei, Pei Zhao, Xiao-Li Pei, Hitoshi Ube, Masahiro Ehara, Mitsuhiko Shionoya

**Affiliations:** a Department of Chemistry, Graduate School of Science, The University of Tokyo 7-3-1 Hongo Bunkyo-ku Tokyo 113-0033 Japan shionoya@chem.s.u-tokyo.ac.jp; b Research Center for Computational Science, Institute for Molecular Science Myodaiji Okazaki Aichi 444-8585 Japan ehara@ims.ac.jp

## Abstract

The properties of metal clusters are highly dependent on their molecular surface structure. The aim of this study is to precisely metallize and rationally control the photoluminescence properties of a carbon(C)-centered hexagold(i) cluster (CAu^I^_6_) using N-heterocyclic carbene (NHC) ligands with one pyridyl, or one or two picolyl pendants and a specific number of silver(i) ions at the cluster surface. The results suggest that the photoluminescence of the clusters depends highly on both the rigidity and coverage of the surface structure. In other words, the loss of structural rigidity significantly reduces the quantum yield (QY). The QY in CH_2_Cl_2_ is 0.04 for [(C)(Au^I^-BIPc)_6_Ag^I^_3_(CH_3_CN)_3_](BF_4_)_5_ (BIPc = *N*-isopropyl-*N*′-2-picolylbenzimidazolylidene), a significant decrease from 0.86 for [(C)(Au^I^-BIPy)_6_Ag^I^_2_](BF_4_)_4_ (BIPy = *N*-isopropyl-*N*′-2-pyridylbenzimidazolylidene). This is due to the lower structural rigidity of the ligand BIPc because it contains a methylene linker. Increasing the number of capping Ag^I^ ions, *i.e.*, the coverage of the surface structure, increases the phosphorescence efficiency. The QY for [(C)(Au^I^-BIPc^2^)_6_Ag^I^_4_(CH_3_CN)_2_](BF_4_)_6_ (BIPc^2^ = *N*,*N*′-di(2-pyridyl)benzimidazolylidene) recovers to 0.40, 10-times that of the cluster with BIPc. Further theoretical calculations confirm the roles of Ag^I^ and NHC in the electronic structures. This study reveals the atomic-level surface structure–property relationships of heterometallic clusters.

## Abstract 2

The properties of metal clusters are highly dependent on their molecular surface structure.

## Graphical abstract

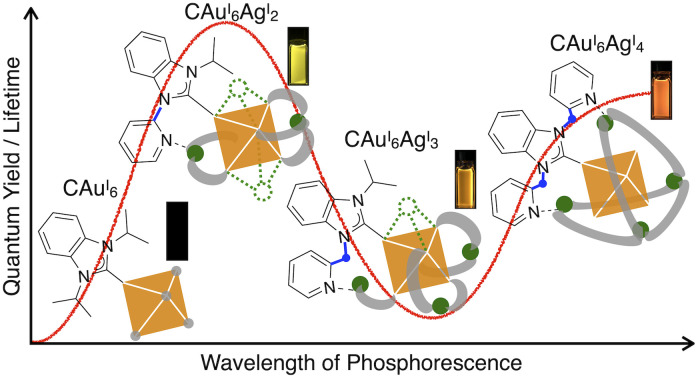

## Introduction

Gold clusters have attracted a lot of attention in recent years due to their molecular structures based on aurophilicity between gold atoms and their optical properties such as structure-specific photoluminescence.^1,2^ Gold clusters with such properties have been applied to a variety of applications such as OLEDs and bioimaging *etc.*^3^ To realize on-demand synthesis of cluster-based phosphorescent materials, great efforts have been made over the past decades to correlate the structure of gold clusters with their photoluminescence.^4–6^ In the meantime, several general strategies were developed to enhance or tune the photoluminescence. Examples include changes in electronic structure due to alloying of metal kernels,^7–10^ surface hardening by additives to suppress non-radiative relaxation,^11–14^ supramolecular networks due to self-assembly of clusters,^15–18^ and chemical modification of supporting ligands by electronic donating/withdrawing groups and use of steric hindrance.^19^

On the other hand, surface molecular design of metal clusters is a promising strategy for developing chemical and physical properties specific to metal arrays. Compared to the widely used phosphine ligands, NHC ligands are similarly stable monodentate ligands with flexible electronic properties such as electron-donating properties and high designability of chemical structures.^20–27^ Therefore, we have been working on photoluminescence control of C-centered Au^I^ and Au^I^–Ag^I^ clusters by chemical modification with *N*-heterocyclic carbene (NHC) ligands.^28–32^ We found that the introduction of different types of ligands on the surface of Au^I^ and Au^I^–Ag^I^ clusters dramatically changed their photoluminescence behavior, including excitation and emission energies, quantum yields (QYs, *φ*) and phosphorescence lifetimes (*τ*). For example, the homometallic [(C)(Au^I^-BI*i*Pr)_6_](BF_4_)_2_ (1, BI*i*Pr = *N*,*N*′-diisopropylbenzimidazolylidene) and the classic [(C)(Au^I^-PPh_3_)_6_](BF_4_)_2_ (ref. 33–36) are structurally similar but exhibit very different emission properties in the solid state.^30^ More surprisingly, the heterometallic [(C)(Au^I^-BIPy)_6_Ag^I^_2_](BF_4_)_4_ (2, BIPy = *N*-isopropyl-*N*′-2-pyridylbenzimidazolylidene) shows blue-shifted emission and 3 times higher QY in solution than [(C)(Au^I^-dppy)_6_Ag^I^_2_](BF_4_)_4_ (dppy = 2-pyridyldiphenylphosphine).^32,37^

Previously, Wang and co-workers reported a series of carbon-centered, phosphine-protected Au^I^–Ag^I^ clusters.^37–44^ The second layer of Ag^I^ ions on the cluster surface fully protects the core portion, resulting in long life and highly efficient emission.^43,44^ In our recent work, we found that NHC ligands may further enhance the emission of heterometallic clusters.^32^ These results suggest that, the silver(i) adatoms and NHC ligands synergistically contribute to the photoluminescence properties of heterometallic clusters.

**Scheme 1:**
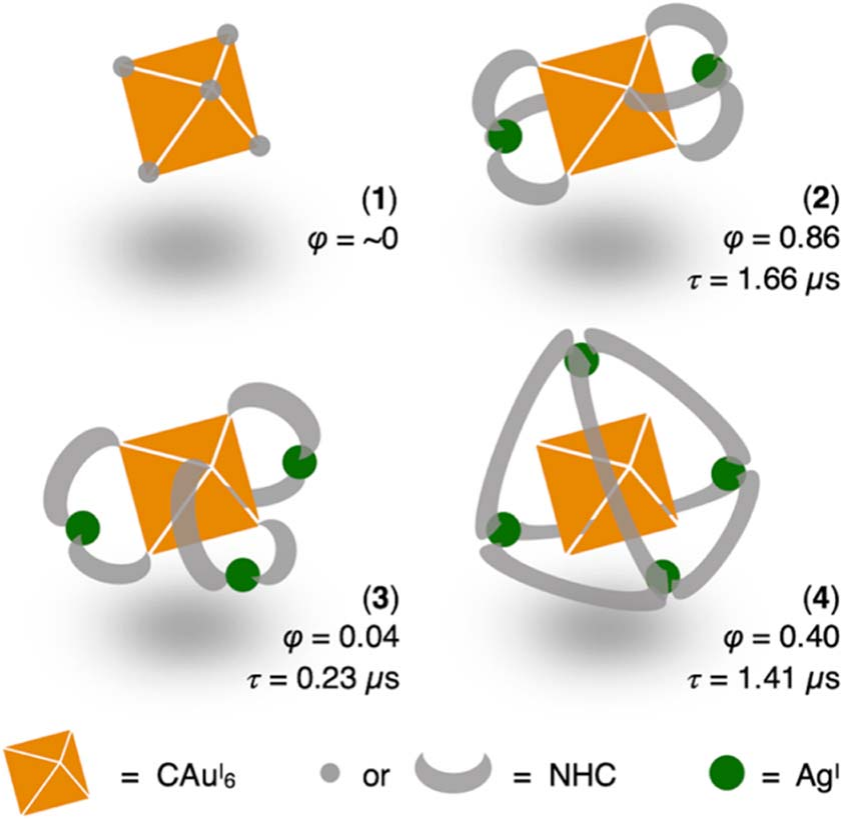
Phosphorescence properties of CAu^I^_6_ clusters surrounded by different NHC or NHC–Ag^I^ shells in CH_2_Cl_2_ at room temperature (*φ* represents QY, *τ* represents lifetime).

In this study, we aimed to investigate this synergistic effects of NHC ligands and the silver(i) adatoms on the surface of heterometallic clusters. By taking advantage of pre-designed benzimidazolylidene-type NHC ligands with one or two picolyl pendant(s), we have achieved atomically precise metallization to the CAu^I^_6_ cluster site, and established correlations among the ligand structure, metal adatom number, and photoluminescence behavior (Scheme 1). We emphasize that the results of this work are not a mirror universe of phosphine-protected clusters, since (a) ligand design here is more elaborate; (b) the heterometallic clusters obtained show distinctly different molecular geometries; and (c) the photoluminescence of the clusters does not simply follow the “full protection” rule.

**Fig. 1:**
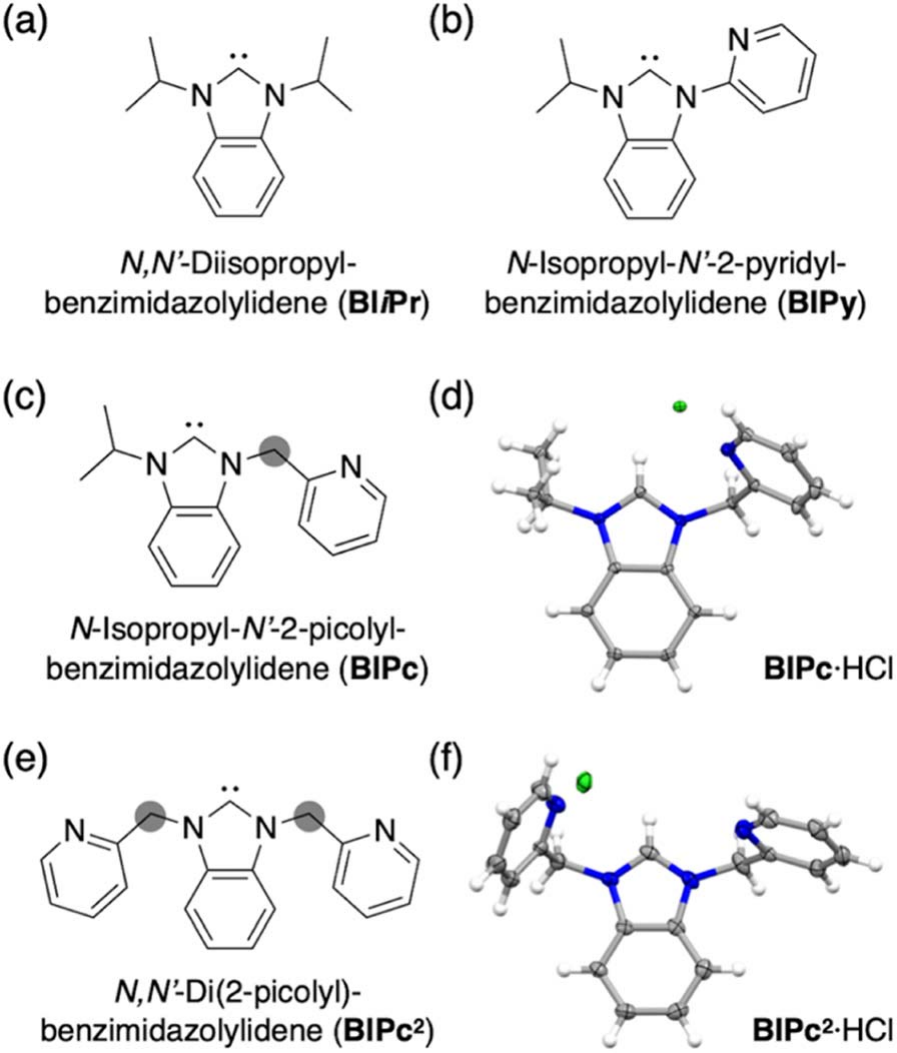
(a) Structure of ligand BI*i*Pr. (b) Structure of ligand BIPy. (c) Structure 2 of ligand BIPc. (d) Molecular structure of BIPc·HCl. (e) Structure of ligand BIPc. (f) Molecular structure of BIPc^2^·HCl.

We first added a methylene linker between the benzimidazole and pyridyl groups of BIPy (Fig. 1). When acting as a bidentate ligand as in the case of BIPy, the methylene group seemed to give some structural flexibility to the resulting ligand *N*-isopropyl-*N*′-2-picolylbenzimidazolylidene (BIPc). Furthermore, since the pyridyl groups of BIPy mainly affect the electronic structure of the hexagold(i) cluster (Scheme S1†),^32^ the fundamental motivation for this design was the thought that the introduced methylene linker could separate the two heterocyclic moieties, thereby breaking the electronic perturbation from the directly connected imidazole and benzimidazole groups. By using this BIPc, a moderately phosphorescent cluster [(C)(Au^I^-BIPc)_6_Ag^I^_3_(CH_3_CN)_3_](BF_4_)_5_ (3) was constructed, in which the octahedral CAu^I^_6_ core was capped by three (BIPc)_2_Ag moieties in a triangular arrangement (Scheme 1). Note that the tricapped octahedral structure has not been achieved yet in neither the NHC- nor the phosphine-protected CAu^I^_6_ cluster family (Scheme S2†). The newly synthesized CAu^I^_6_Ag^I^_3_-type cluster 3 showed a red-shifted emission, but the QY was significantly lower (*φ* = 0.04) and the lifetime was shorter (0.23 μs) compared to the excellent photophysical properties of bicapped octahedral, CAu^I^_6_Ag^I^_2_-type cluster 2. The use of the picolyl group resulted in the coordination of another silver ion around the octahedral CAu^I^_6_ structure, causing a red-shift in the emission. On the other hand, the introduction of a methylene linker to the picolyl group resulted in a much looser structure of the coordination sphere, which was found to be responsible for the low QY and short lifetime.

Next, the carbene ligand *N*,*N*′-di(2-picolyl)benzimidazolylidene (BIPc^2^), which has a higher symmetry with two picolyl groups, was used to make the structure of the coordination sphere more rigid (Fig. 1).^45–47^ As a result, a tetrahedral form of [(C)(Au^I^-**BIPc**^**2**^)_6_Ag^I^_4_(CH_3_CN)_2_](BF_4_)_6_ (4), a CAu^I^_6_Ag^I^_4_-type cluster, was obtained in which the outer shell part composed of (BIPc^2^)_6_Ag^I^_4_ covered the octahedral CAu^I^_6_ cluster (Scheme 1). By introducing an additional ligand in the wingtip group of the carbene ligand and increasing the number of Ag^I^ ions bound to CAu^I^_6_, strong red phosphorescence with a QY of 0.40 and a lifetime of 1.41 μs was observed in CH_2_Cl_2_, both of which were significantly improved compared to cluster 3. The CAu^I^_6_Ag^I^_4_ tetrahedron has edges of 5.544–5.947 Å, which is comparable to the Au_10_ nanocluster,^48,49^ one of the long pursued tetrahedral gold nanoclusters. This result indicates that not only the coverage of the cluster surface by additional metal ions but also the rigidity of the coordination structure is important for the strong and long-lived phosphorescent properties of metal clusters. The structure will also provide a useful new idea for rational synthesis of tetrahedral Au_10_ and Au_20_.^48–54^

## Results and discussion

### Synthesis

**Fig. 2:**
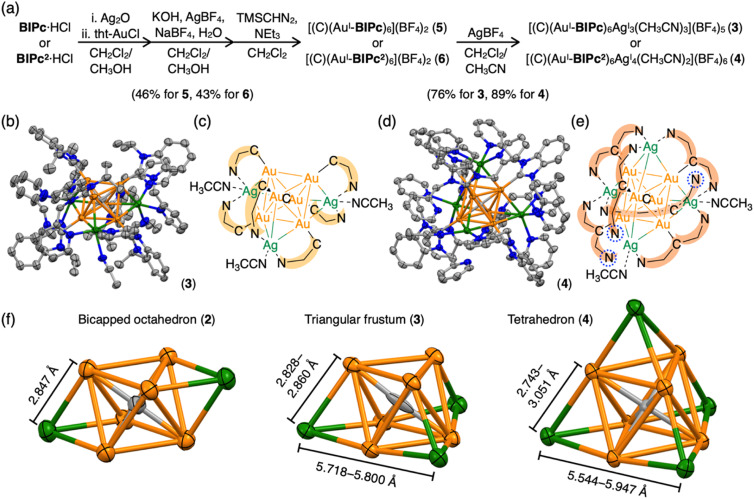
(a) Synthetic schemes of the homometallic clusters [(C)(Au^I^-BIPc)_6_](BF_4_)_2_ (5) and [(C)(Au^I^-BIPc^2^)_6_](BF_4_)_2_ (6), and heterometallic clusters [(C)(Au^I^-BIPc)_6_Ag^I^_3_(CH_3_CN)_3_](BF_4_)_5_ (3) and [(C)(Au^I^-BIPc^2^)_6_Ag_4_(CH_3_CN)_2_](BF_4_)_6_ (4). (b) Molecular structure of cluster 3. (c) A simplified scheme showing the structure of cluster 3. C^N represents ligand BIPc. (d) Molecular structure of cluster 4. (e) A simplified scheme showing the structure of cluster 4. N^C^N represents ligand BIPc^2^. Uncoordinated pyridyl groups are highlighted by dashed blue cycles. (f) Comparison of structures of bicapped octahedral CAu^I^_6_Ag^I^_2_ in cluster 2, triangular frustum-shaped CAu^I^_6_Ag^I^_3_ in cluster 3, and tetrahedral CAu^I^_6_Ag^I^_4_ in cluster 4. The length of the edge of each structure is shown.

Structures of BI*i*Pr, BIPy, BIPc and BIPc^2^ are shown in Fig. 1. The ligands BIPc and BIPc^2^ were synthesized as hydrochloride salts by a modified procedure of the literature (Fig. 1 and S1†). The CAu^I^_6_ clusters [(C)(Au^I^-BIPc)_6_](BF_4_)_2_ (5) and [(C)(Au^I^-BIPc^2^)_6_](BF_4_)_2_ (6) as precursors were prepared in 46% and 43% overall yields based on the amount of gold, respectively (Fig. 2a, S2–S22 and Tables S1–S3†).§§Crystallographic data for BIPc·HCl: C_16_H_18_N_3_Cl, *a* = 9.26934(12), *b* = 14.15286(15), *c* = 12.30478(15) Å, *β* = 111.3536(14)°, *V* = 1503.42(3) Å^3^, monoclinic space group *P*2_1_/*c*, *Z* = 4, *T* = 93(2) K, 9271 reflections measured, 2931 unique (*R*_int_ = 0.0357), final *R*_1_ = 0.0366, *wR*_2_ = 0.0974 for 2797 observed reflections [*I* > 2*σ*(*I*)]. For BIPc^2^·HCl: C_19_H_17_N_4_Cl·H_2_O, *a* = 13.3991(2), *b* = 9.82400(10), *c* = 13.8269(2) Å, *β* = 105.9170(10)°, *V* = 1750.29(4) Å^3^, monoclinic space group *P*2_1_/*n*, *Z* = 4, *T* = 93(2) K, 8598 reflections measured, 3359 unique (*R*_int_ = 0.0176), final *R*_1_ = 0.0393, *wR*_2_ = 0.1039 for 3247 observed reflections [*I* > 2*σ*(*I*)]. For [(C)(Au^I^-BIPc)_6_Ag_3_(CH_3_CN)_3_](BF_4_)_5_ (3): C_103_H_111_B_5_N_21_F_20_Ag_3_Au_6_, *a* = 28.2746(8), *b* = 33.2019(8), *c* = 29.4805(10) Å, *β* = 106.484(3)°, *V* = 26 537.9(14) Å^3^, monoclinic space group *I*2/*a*, *Z* = 8, *T* = 93(2) K, 62 923 reflections measured, 25 505 unique (*R*_int_ = 0.0481), final *R*_1_ = 0.0970, *wR*_2_ = 0.2906 for 15 540 observed reflections [*I* > 2*σ*(*I*)]. For [(C)(Au^I^-BIPc^2^)_6_Ag_4_(CH_3_CN)_2_](BF_4_)_6_ (4): C_119_H_102_B_6_N_26_F_24_Ag_4_Au_6_·2CH_2_Cl_2_·CH_3_CN, *a* = 35.5410(3), *b* = 15.07664(17), *c* = 50.5683(5) Å, *β* = 93.2056(9)°, *V* = 27 054.0(5) Å^3^, monoclinic space group *I*2/*a*, *Z* = 8, *T* = 93(2) K, 74 304 reflections measured, 26 513 unique (*R*_int_ = 0.0522), final *R*_1_ = 0.0605, *wR*_2_ = 0.1686 for 22 778 observed reflections [*I* > 2*σ*(*I*)]. For [(C)(Au^I^-BIPc)_6_](BF_4_)_2_ (5): C_97_H_102_B_2_N_18_F_8_Au_6_·CH_2_Cl_2_, *a* = 13.57251(16), *b* = 22.5778(2), *c* = 17.07448(17) Å, *β* = 99.9220(11)°, *V* = 5153.99(10) Å^3^, monoclinic space group *P*2_1_/*n*, *Z* = 2, *T* = 93(2) K, 31 050 reflections measured, 10 136 unique (*R*_int_ = 0.0363), final *R*_1_ = 0.0496, *wR*_2_ = 0.1311 for 8499 observed reflections [*I* > 2*σ*(*I*)]. For [(C)(Au^I^-BIPc^2^)_6_](BF_4_)_2_ (6): C_115_H_96_B_2_N_24_F_8_Au_6_·CH_2_Cl_2_, *a* = 14.44142(13), *b* = 43.0804(4), *c* = 17.47381(12) Å, *β* = 92.7197(7)°, *V* = 10 858.95(16) Å^3^, monoclinic space group *P*2_1_/*c*, *Z* = 4, *T* = 93(2) K, 59 653 reflections measured, 21 354 unique (*R*_int_ = 0.0392), final *R*_1_ = 0.0379, *wR*_2_ = 0.0949 for 18 478 observed reflections [*I* > 2*σ*(*I*)]. All crystallographic data have been deposited at the CCDC under deposition numbers CCDC 2153516 (for BIPc·HCl), 2153517 (for BIPc^2^·HCl), 2153518 (for 3), 2153519 (for 4), 2153520 (for 5), and 2153521 (for 6). CAu^I^_6_Ag^I^_2_-type cluster 2 was synthesized according to our previously reported literature.^32^

Next, heterometallic clusters were synthesized from clusters 5 and 6. The CAu^I^_6_ cluster of 5 or 6 was mixed with AgBF_4_ in CH_2_Cl_2_/CH_3_CN (v : v = 9 : 1) and the reaction was monitored by ESI-MS spectrometry. When AgBF_4_ was added to cluster 5, signals indicating the formation of heterometallic species of [(C)(Au^I^-BIPc)_6_Ag^I^](BF_4_)^2+^, [(C)(Au^I^-BIPc)_6_Ag^I^_2_](BF_4_)_2_^2+^, [(C)Au^I^_5_Ag^I^(BIPc)_4_](BF_4_)^+^, and [(C)Au^I^_5_Ag^I^_2_(BIPc)_4_](BF_4_)_2_^+^ appeared (Fig. S23†), which were found to be similar to the pattern of peaks seen in the BIPy-protected cluster.^32^ This complexation process in solution was also monitored by UV-vis absorption, fluorescence and ^1^H NMR spectroscopies (Fig. S24–S26†). In the UV-vis spectra, the intensity of the peak around 340 nm decreased significantly with the addition of silver ions, and a new series of peaks and shoulders appeared. The emission spectra showed an increase in the intensity of the broad peak in the lower energy region. In fact, when the reaction mixture was irradiated with a UV lamp, a bright orange-yellow emission was observed. The decrease and increase in absorbance at 343 and 440 nm, respectively, and the increase in emission intensity at 609 nm were all strongly suggestive of the incorporation of three Ag^I^ ions into cluster 5. The ^1^H NMR spectra showed that the sharp signals became unstructured with the addition of Ag^I^ ions, suggesting a flexible coordination structure of the product.

The complexation between cluster 6 and AgBF_4_ in CH_2_Cl_2_/CH_3_CN was first monitored by ^1^H NMR spectroscopy. The heterometallic species formed *in situ* exhibited a single set of relatively sharp signals, distinct from the complexation of cluster 5 and AgBF_4_ (Fig. S26 and 27†). ESI-MS spectroscopic analysis revealed the gradual formation of heterometallic species ([(C)(Au^I^-BIPc^2^)_6_Ag^I^_*n*_](BF_4_)_*n*_^2+^ (*n* = 1–4)) containing one to four Ag^I^ ions (Fig. S28†). In the UV-vis absorption and emission spectra, a plot of absorbance/emission intensity against the amount of Ag^I^ added to cluster 6 indicates that three to four Ag^I^ ions were incorporated into cluster 6 (Fig. S29 and 30†). These data strongly suggest that a tetra-capped heterometallic structure was formed.

### Molecular structures and characterizations

Single crystals of heterometallic clusters were obtained by layering dry Et_2_O on a CH_2_Cl_2_/CH_3_CN solution of products in tubes. Single-crystal X-ray diffraction (ScXRD) analysis determined the structures of the tricapped CAu^I^_6_Ag^I^_3_-type 3 and the tetrahedral CAu^I^_6_Ag^I^_4_-type 4.§ The molecular structure and simplified scheme of the cationic part of cluster 3 are shown in Fig. 2b and c, respectively. The structure of 3 is clearly different from that of CAu^I^_6_Ag^I^_2_-type cluster 2, as the three Ag^I^ atoms, supported by the ligand BIPc, cap the three octahedral faces of the CAu^I^_6_ cluster, forming a triangulated monorectified tetrahedron (triangular frustum) structure. Specifically, two BIPc ligands adjacent *via* pyridyl groups and one additional acetonitrile ligand are coordinated to each Ag^I^ ion in cluster 3, so the overall structure can be viewed as a CAu^I^_6_ center supported by three (BIPc)_2_Ag motifs. The reason why the synthesis in CH_2_Cl_2_/CH_3_OH did not work (Fig. S31 and S32, see ESI† for details) can be explained by the co-stabilization with acetonitrile. The Au^I^–Ag^I^ distances in cluster 3 (2.8268(14)–2.9754(14) Å, Table S4†) were found slightly longer than that in cluster 2 (2.8467(17) Å). The ligand BIPc has one more methylene group than BIPy, making it relatively flexible. Notably, when cluster 3 was formed, the ^1^H NMR spectra showed that the singlet signal of the methylene group of CAu^I^_6_-type cluster 5 (*δ* = 5.77 ppm in CD_2_Cl_2_) was split into two doublet signals (*δ* = 6.10, 6.66 ppm in CD_2_Cl_2_) (Fig. S4 and S33†). This is because, when the pyridyl group coordinates to Ag^I^, the two hydrogen atoms of the methylene group become chemically inequivalent. One hydrogen atom is oriented toward the metal kernel to form a hydrogen-gold bond, while the other is oriented away from it. Indeed, in the structure of cluster 3, the estimated intramolecular C(sp^3^)–H⋯Au bond between the hydrogen atom and the gold atom was very short^55–60^ (Fig. S37,† 2.677 Å). The results of the ^1^H NMR measurements of cluster 3 were found to be affected from the deuterated solvent in the solvent used. In fact, very broadened signals were observed when measured in CD_2_Cl_2_/CD_3_CN (v : v = 9 : 1, Fig. S38†), reproducing the results of the complexation between cluster 5 and AgBF_4_ (Fig. S26†). This result suggests that the presence of coordinating acetonitrile molecules makes the coordination of ligand BIPc in cluster 3 slightly more fluxional. UV-vis absorption spectroscopy and ESI-MS were also employed to characterize cluster 3 (Fig. S39 and S40†). The results were similar to the spectra of heterometallic species formed *in situ* (Fig. S23 and S24†).

More interestingly, CAu^I^_6_Ag^I^_4_-type cluster 4 has a tetrahedral structure (Fig. 2d and e). Tetrahedral gold clusters (*e.g.* Au_4_, Au_10_, Au_20_) have long been explored because they can be regarded as fragments of bulk gold and have been found or predicted to exhibit unique structural properties such as wide energy gap and spherical aromaticity.^48–54,61–63^ Despite the fact that Au_4_ has actually been reported, structure information on the latter two clusters is not yet available. As shown in the simplified CAu^I^_6_Ag^I^_4_ structure in Fig. 2e, four Ag^I^ atoms cap the four faces of the octahedral CAu^I^_6_ in the central of cluster 4. The Au^I^–Ag^I^ distances for cluster 4 are in the range of 2.7425(7) to 3.0507(7) Å, slightly off distances in 2 and 3. Another way to look at it is that the central CAu^I^_6_ is encapsulated in a tetrahedral (BIPc^2^)_6_Ag_4_ cage. Three of the twelve pyridyl groups were found not to be involved in metal coordination in the crystalline state, as indicated by the blue dashed circles. The coordinating acetonitrile was thought to contribute to the stabilization. Similarly, in the case of cluster 3, two pyridyl groups and one acetonitrile are considered to be uniquely coordinated to the three Ag^I^ ions to stabilize the complex in solution. In the case of cluster 4, on the other hand, the ligand exchange reaction in solution is considered to be more complex because there can be multiple combinations of pyridyl groups and acetonitrile as ligands to the four Ag^I^ ions. The mismatched structure of the ligand BIPc^2^ probably prevents the formation of a perfect tetrahedral structure.

An important structural point is that the composition of 4 is similar to previously reported [(C)(Au^I^-PPhPy_2_)Ag^I^_4_](BF_4_)_6_ (PPhPy_2_ = bis(2-pyridyl)phenylphosphine), but the arrangement of four Ag^I^ ions on the octahedral CAu^I^_6_ surface is distinctly different from each other.^43^ The latter cluster has a butterfly-like molecular structure, that is, two opposite pairs of neighboring triangular faces are capped; whereas the four Ag^I^ ions in 4 are in a tetrahedral arrangement as far apart from each other as possible (Scheme S2†). This difference may be attributed to the different geometries of the NHC and phosphine ligands. In PPhPy_2_, the two coordinative pyridyl groups are bonded directly connected to the same phosphorous atom, so the angle between these two pyridyl groups should be approximately 120°, and the distance between the two N donors are relatively short. These two features are in geometrical contrast to BIPc^2^, in which the two picolyl groups are on two sides of the benzimidazolylidene. The introduction of a methylene linker at the picolyl group further increased the distance between the two N donors in BIPc^2^, making the coordination mode more flexible. These results demonstrate the designability of the NHC ligands and their superiority.

For heterometallic cluster 4, we first performed a detailed evaluation of the solution structure by ^1^H NMR spectroscopy (Fig. S41–S44†) and observed only one set of signals in the spectrum. Second, all peaks in the ^1^H and ^13^C NMR spectra were sharp and showed no apparent fluxing, despite the use of a deuterated mixed solvent containing coordinating acetonitrile. This suggests that cluster 4 exists in a fairly symmetric structure in solution. A splitting of the methylene signal was also observed in the ^1^H NMR spectrum of 4, as in cluster 3. The sharp singlet peak at 5.63 ppm in CAu^I^_6_ cluster 6 split into two doublet peaks at 5.38 and 6.06 ppm when forming cluster 4 (Fig. S8 and S41†). This result suggests that the hydrogen atom on one side of the methylene chain of cluster 4 forms an intramolecular C(sp^3^)–H⋯Au bond and the pyridyl group is fixed in solution, as shown in Fig. S37.† Cluster 4 was further characterized by ESI-MS and UV-vis spectroscopy (Fig. S45 and S46†). As a result, we were able to reproduce most of the data for the heterometallic products formed at the site (Fig. S28 and S29†). This indicates that cluster 4 is the main product of the complexation of CAu^I^_6_ cluster 6 with AgBF_4_.

### Correlation between phosphorescence and structure

Clusters 2–4 have a common CAu^I^_6_ octahedral core structure and exhibit properties characteristic of different external structures. In clusters 2–4, the two, three, and four faces of the central octahedral CAu^I^_6_ were capped by Ag^I^ ions, forming bicapped octahedral, triangular frustum-shaped (monorectified tetrahedral), and tetrahedral metal kernels, respectively (Fig. 2f). In cluster 2, the Au^I^–Ag^I^ distance is 2.847 Å. In cluster 3, the side edges of the frustum (Au^I^–Ag^I^) ranged from 2.828 to 2.860 Å and the lower bottom (Ag^I^–Ag^I^) side edges ranged from 5.718 to 5.800 Å. Tetrahedral cluster 4 was slightly distorted, with all edges in the 5.544–5.947 Å range. In the case of NHC ligands, on the other hand, the rigidity of the entire molecule depends on the presence or absence of a methylene linker in the ligand. As a result, it is inferred that clusters 2–4 exhibit structure-specific emission.

**Fig. 3:**
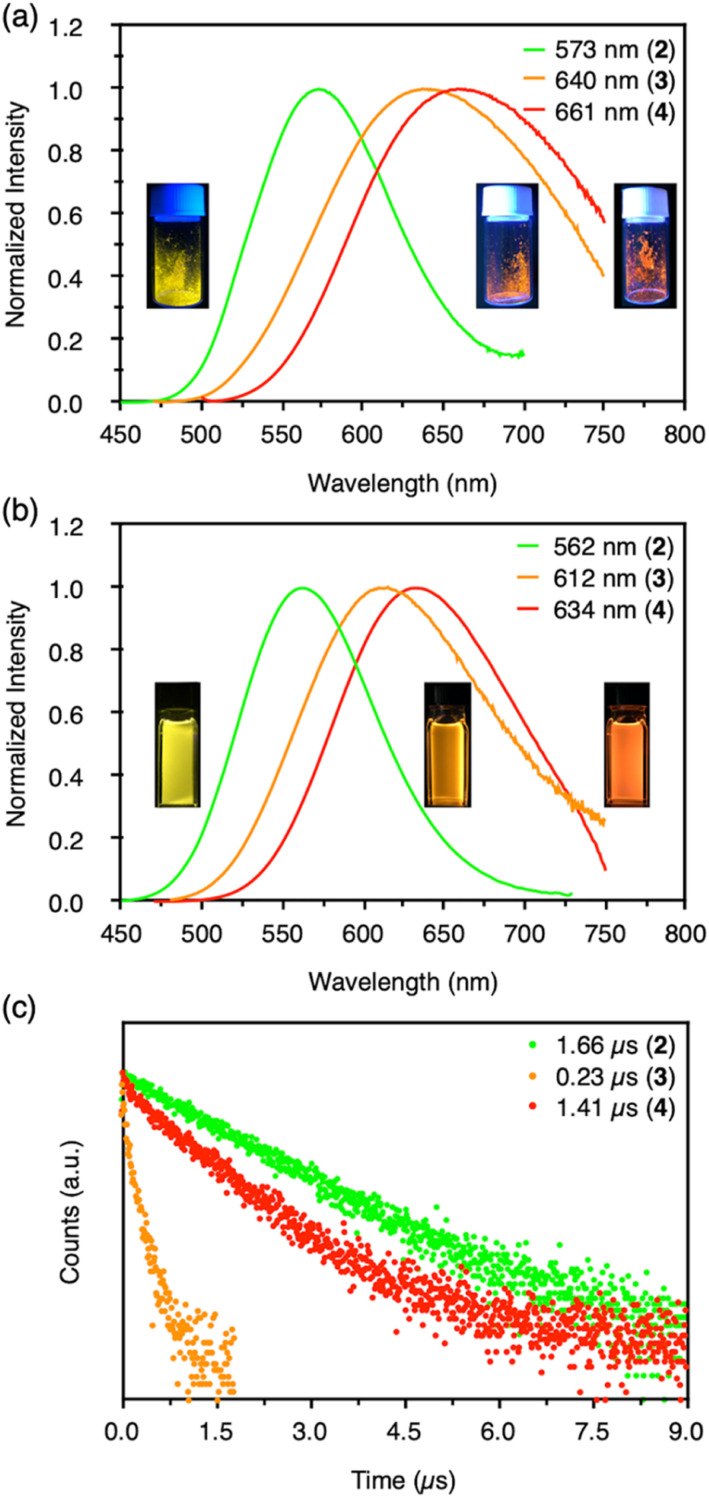
(a) Emission spectra of CAu^I^_6_Ag^I^_*n*_-type clusters 2–4 (*n* = 2, 3, 4) in the solid state at room temperature and corresponding emission photographs of 365 nm excitation (from left to right). (b) Emission spectra of clusters 2–4 in CH_2_Cl_2_ at 20 °C and corresponding emission photographs of 365 nm excitation (from left to right). (c) Emission decays of clusters 2–4 in CH_2_Cl_2_ at room temperature.

In the solid state, clusters 3 and 4 show orange and red emission with maxima at 640 and 661 nm, respectively, both significantly red-shifted compared to cluster 2 (573 nm; Fig. 3a, S47 and S48†). The emission maxima of clusters 2–4 also showed a similar red-shift in the CH_2_Cl_2_ solution (Fig. 3b, S49 and S50†). Both clusters 3 and 4 are very stable under ambient conditions. The absorption spectra of CH_2_Cl_2_ solutions of 3 and 4 (*c* = 3.3 × 10^−5^ mol L^−1^) remain virtually unchanged after one week in sunlight at room temperature (Fig. S51†). Surprisingly, 4 remains robust in CH_2_Cl_2_/CH_3_CN (v : v = 9 : 1, *c* = 3.3 × 10^−5^ mol L^−1^) at 4 °C for about 18 months (Fig. S52†). The wingtip group of ligand BIPc in cluster 3 is one methylene chain longer than that of BIPy in cluster 2, so that one more Ag^I^ ion could be accommodated on the central hexagold(i) cluster. In the case of cluster 4, the number of pyridyl groups increased to twelve, creating new chances to bind further one more adatom. As a result, more red-shifted emission was observed from the clusters with more metal atoms, and the stability of cluster 4 was also improved by introducing a fourth silver atom.

More importantly, the photoluminescence QYs and lifetimes of clusters 2–4 were very different from each other. First, the QY and lifetime of cluster 3 were measured to be 0.04 and 0.23 μs, respectively, about an order of magnitude smaller than those of cluster 2 (0.86 and 1.66 μs, respectively). However, these values for cluster 4 were 0.40 and 1.41 μs, respectively, almost recovering to the level of the values for cluster 2. The high QY, microsecond-scale lifetime, and large Stokes shift indicate that the emissions of clusters 2–4 are phosphorescence.

The radiative and non-radiative rate constants (*k*_r_ and *k*_nr_, respectively) of each cluster were then calculated from the QY and lifetime values (Table 1). Because all of these clusters are protected by benzimidazolylidene-type ligands, we were able to correlate two factors with respect to the emissions of clusters: the wingtip groups of the carbene ligand, and number of Ag^I^ atoms in the cluster.

**Table 1:** Emission QYs (*φ*), lifetimes (*τ*), radiative (*k*_r_) and non-radiative (*k*_nr_) rate constants of CAu^I^_6_Ag^I^_*n*_-type clusters 2–4 (*n* = 2, 3, 4) in CH_2_Cl_2_

Cluster	*φ*	*τ* (μs)	*k*_r_ (×10^5^ s^−1^)	*k*_nr_ (×10^5^ s^−1^)
2	0.86	1.66	5.18	0.84
3	0.04	0.23	1.74	41.7
4	0.40	1.41	2.84	4.26

First, the values of *k*_r_ and *k*_nr_ for CAu^I^_6_Ag^I^_3_-type cluster 3 were changed by a factor of 1/3 and about 50 times, respectively, compared to those of cluster 2. These marked differences are likely due to a significant decrease in the stability of the bond between the methylene group of the BIPc ligand of cluster 3 and the Ag^I^ ion (Scheme S2†). This increase in the non-radiation deactivation process of 3 is due to the introduction of a flexible structure, which may have had a significant impact on the emission efficiency. Furthermore, the *k*_nr_ value of CAu^I^_6_Ag^I^_4_-type cluster 4 decreased by about a factor of 10, indicating that non-radiation deactivation process was significantly suppressed. In cluster 3, the six ligands have one picolyl ligand each, with two picolyl nitrogens and one acetonitrile coordinating to the three Ag^I^ ions. In cluster 4, six ligands have two picolyl groups each, and most or all of them can interact with the four Ag^I^ atoms, as shown by ScXRD and NMR data. Considering that the bond formation between the picolyl group and Ag^I^ has a small effect on the bond lengths and bond angles of the methylene-bridged N–C–C bonds in the ligand moiety of clusters 3 and 4, it is estimated that the incorporation of the fourth Ag^I^ atom into the CAu^I^_6_ core of cluster 4 serves to further enhance QY by efficiently suppressing the non-radiation process, and that the immobilizing effect of the picolyl groups, although not negligible, is not a decisive factor.

### Theoretical calculations

Density functional theory (DFT) and time-dependent density functional theory (TD-DFT) calculations were carried out to disclose electronic structures of the clusters and the origin of the phosphorescence. First, the Bader and NPA (natural population analysis) charges of the central C, Au and Ag atoms in CAu^I^_6_Ag^I^_3_-type cluster 3 and CAu^I^_6_Ag^I^_4_-type cluster 4 were analyzed (Table S5†). Compared to CAu^I^_6_-type clusters 5 and 6, the negative charges of the central C atoms in clusters 2, 3 and 4 are reduced and the positive charges of the Au atoms are reduced. These results indicate that Ag ions covering the CAu^I^_6_ kernel affect the charge distribution of the metal clusters. Furthermore, the larger the number of Ag ions, the greater the effect on the charge distribution.

**Fig. 4:**
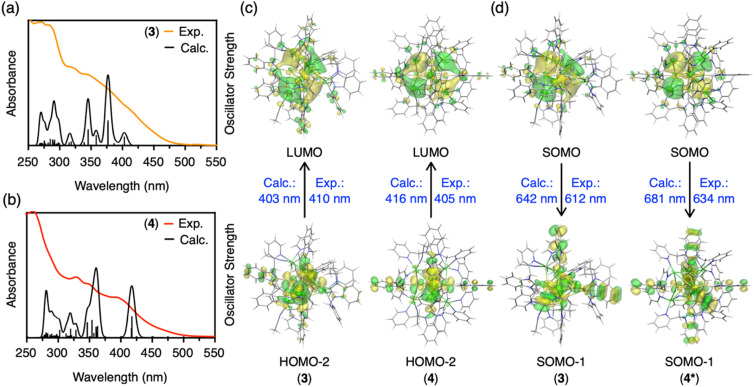
(a) Calculated and experimental UV-vis absorption spectra of cluster 3. (b) Calculated and experimental UV-vis absorption spectra of cluster 4. (c) Calculated HOMO and LUMO, and comparison of calculated and experimental excitation energies of clusters 3 and 4. (d) Calculated SOMOs (singly occupied molecular orbitals, in the triplet state), and comparisons of calculated and experimental emission energies of clusters 3 and 4*.

Next, we simulated the UV-vis absorption spectra of 3 and 4. As shown in Fig. 4a and b, the appearance of shoulder bands in the lower energy region (∼410 nm for cluster 3, 405 nm for cluster 4) was well reproduced. The first peak at 403 nm of cluster 3 is mainly due to the transition from HOMO−2 (highest occupied molecular orbital) to LUMO (lowest unoccupied molecular orbital), as shown in Fig. 4c, S54 and Table S6.† The strongest peak at 377 nm is mostly derived from transitions between three occupied molecular orbitals (HOMO, HOMO−1, and HOMO−2) and two unoccupied molecular orbitals (LUMO and LUMO+1). The occupied MOs are localized in the Au kernel, and a large contribution from Ag atoms is observed in the unoccupied MOs. In the case of 4 with four Ag atoms, an onset peak appears around 416 nm, with the strongest peak located around 359 nm. As summarized in Table S7 and Fig. S55,† these absorption peaks originate from transitions among MOs of Au and Ag, and the distribution of orbitals involved is similar to that of 3. Orbital composition analysis by Mulliken partition was then performed for 2–6 (Table S8†). In all clusters, the central C and Au atoms show large contributions (∼70%) to the occupied molecular orbitals (HOMO, HOMO−1, and HOMO−2). For the virtual molecular orbitals (LUMO+2, LUMO+1, and LUMO), the ligands contribute significantly (∼60%) in homometallic clusters 5 and 6, while the silver atoms contribute mainly in LUMO (∼30–40%) in heterometallic clusters 2–4.

Finally, we calculated the lowest triplet excited state to estimate the phosphorescence energies of CAu^I^_6_Ag^I^_3_-type cluster 3 and CAu^I^_6_Ag^I^_4_-type cluster 4, which were determined as 1.94 (642 nm) and 1.97 eV (629 nm), respectively (Fig. 4d, S56 and Table S9†). However, these results were in conflict with the experimental results, in which clusters 2–4 clearly showed a trend of red-shift. Considering that cluster 4 maintains a fairly symmetric structure in solution, as indicated by the NMR results, we removed the two acetonitrile molecules from cluster 4 and constructed a highly symmetric structure with a *T* point group, namely [(C)(Au^I^-BIPc^2^)_6_Ag^I^_4_](BF_4_)_6_ (4*, Table S10 and Fig. S57, S58†). The phosphorescence energy of 4* was calculated to be 1.82 eV (681 nm), supporting a red-shift compared to cluster 3. As shown in Fig. 4d, the SOMOs of clusters 3 and 4* were mainly located in the silver atoms of the clusters, similar to the LUMOs of clusters 3 and 4. On the other hand, a large contribution from the benzimidazolylidene motif of the ligand was observed in the SOMO−1 orbitals of clusters 3 and 4*. These are very different from the occupied MOs of clusters 3 and 4, because the central CAu^I^_6_ cores are more involved in HOMO−2 *etc.* Thus, the calculations indicate that the contribution of the outer shells (ligands and silver atoms) in the phosphorescent relaxation process is significant. Theoretical evaluation of the phosphorescence lifetimes and radiative rate constants of 3 and 4* lead to similar conclusions that the outer shells are significantly involved in each state, which changed the coupling of the spin–orbit states of each cluster (Tables S11 and S12, for the details see ESI†).

## Conclusions

By using pre-designed NHC ligands with one pyridyl, or one or two picolyl pendants, we have achieved atomically precise surface metallization of C-centered Au^I^_6_ clusters with a specific number of silver(i) atoms. It was confirmed experimentally and theoretically that the NHC–Ag^I^ shells contribute significantly to the photoluminescence properties of the resulting heterometallic clusters. Specifically, reducing rigidity will favor non-radiative pathways more, while increasing coverage and protection will efficiently suppress those pathways. This study has precisely elucidated the relationship between heterometallic cluster structure and phosphorescent properties at the atomic level. Advancements in basic design methods for highly photoluminescent metal assemblies confined in organic and organometallic shells are expected.

## Data availability

All the data are shown in the ESI.†

## Author contributions

Z. L. performed the synthesis, characterizations and analysed the data. P. Z. performed the theoretical calculations. X.-L. P. assisted in the synthesis and characterizations. H. U., M. E. and M. S. directed the study. All authors prepared the manuscript.

## Conflicts of interest

There are no conflicts to declare.

## Supplementary Material

SC-014-D3SC01976D-s001

SC-014-D3SC01976D-s002
