# Supplementary material for: Photoluminescence control by atomically precise surface metallization of C-centered hexagold(i) clusters using N-heterocyclic carbenes
Source: Chem Sci. 2023 May 11;14(23):6207–15. doi: 10.1039/d3sc01976d (PMC10266449; doi:10.1039/d3sc01976d)
Supplement: SC-014-D3SC01976D-s001 [file SC-014-D3SC01976D-s001.pdf]

Electronic Supplementary Material (ESI) for Chemical Science.
This journal is © The Royal Society of Chemistry 2023

Electronic Supplementary Information

Photoluminescence control by atomically precise surface metallization of *C*-centered hexagold(I) clusters using *N*-heterocyclic carbenes

Zhen Lei,[a†] Pei Zhao,[b†] Xiao-Li Pei,[a] Hitoshi Ube,[a] Masahiro Ehara*[b] and Mitsuhiko Shionoya*[a]

[a]Department of Chemistry, Graduate School of Science, The University of Tokyo, 7-3-1 Hongo, Bunkyo-ku, Tokyo 113-0033, Japan. E-mail: shionoya@chem.s.u-tokyo.ac.jp
[b]Research Center for Computational Science, Institute for Molecular Science, Myodaiji, Okazaki, Aichi 444-8585, Japan. E-mail: ehara@ims.ac.jp
[†]These authors contributed equally to this work

1. Materials and reagents
2. Physical measurements and instrumentation
3. Synthesis and characterizations
3.1 **BIPc**·HCl and **BIPc^2**·HCl
3.2 Homometallic CAu^I_6 clusters $[(C)(Au^I$-**BIPc**$)_6](BF_4)_2$ (**5**) and $[(C)(Au^I$-**BIPc^2**$)_6](BF_4)_2$ (**6**)
3.3 Complexation experiments of **5** and **6** with $AgBF_4$
3.4 Heterometallic $CAu^I_6Ag^I_3$ cluster $[(C)(Au^I$-**BIPc**$)_6Ag^I_3(CH_3CN)_3](BF_4)_5$ (**3**) and $CAu^I_6Ag^I_4$ cluster $[(C)(Au^I$-**BIPc^2**$)_6Ag^I_4(CH_3CN)_2](BF_4)_6$ (**4**)
4. X-ray crystallography
5. Computation details
6. Additional results and discussion on the homometallic CAu^I_6 clusters 5 and 6
7. Additional results and discussion on the complexation experiments of 5 and 6 with $AgBF_4$ in CH_2Cl_2/CH_3OH (9:1, v:v)
8. Additional results and discussion on the theoretical calculations of phosphorescence lifetimes and radiative rate constants of 3 and $[(C)(Au^I$-BIPc$^2)_6Ag^I_4](BF_4)_6$ (4*)
9. Supplementary schemes, figures, and tables
Scheme S1. Aromatic wingtip group of (benz)imidazolylidene may significantly contribute to the photophysical properties of corresponding metal clusters
Scheme S2. Coordination modes of ligands, structures of metal kernels, and QYs (in CH_2Cl_2) of CAu^I_6-based clusters protected by NHC and phosphine ligands
Figure S1. ^{1}H NMR spectrum of **BIPc**·HCl (500 MHz, $CDCl_3$, 300 K)
Figure S2. Molecular structures of **5** and **6**
Table S1. Key structural parameters of **5**, **6**, and some reported CAu^I_6 clusters
Figure S3. Schematic illustration of intramolecular Au···H−C bonds in **5** and **6**
Figure S4. ^{1}H NMR spectrum of **5** (500 MHz, CD_2Cl_2, 300 K)
Figure S5. ^{1}H-^{1}H COSY NMR spectrum of **5** (500 MHz, CD_2Cl_2, 300 K)
Figure S6. ^{13}C NMR spectrum of **5** (126 MHz, CD_2Cl_2, 300 K)
Figure S7. ^{1}H-^{13}C HSQC spectrum of **5** (500 MHz, CD_2Cl_2, 300 K)
Figure S8. ^{1}H NMR spectrum of **6** (500 MHz, CD_2Cl_2, 300 K)
Figure S9. ^{1}H-^{1}H COSY NMR spectrum of **6** (500 MHz, CD_2Cl_2, 300 K)
Figure S10. ^{13}C NMR spectrum of **6** (126 MHz, CD_2Cl_2, 300 K)
Figure S11. ^{1}H-^{13}C HSQC spectrum of **6** (500 MHz, CD_2Cl_2, 300 K)
Figure S12. UV-vis absorption spectrum of **5** in CH_2Cl_2
Figure S13. UV-vis absorption spectrum of **6** in CH_2Cl_2
Figure S14. ESI MS spectra of **5**
Figure S15. ESI MS spectra of **6**
Figure S16. Excitation and emission spectra of **5** in the solid state
Figure S17. Excitation and emission spectra of **6** in the solid state
Figure S18. Optimized structures of **5** and **6**
Figure S19. Simulated and experimental UV-vis absorption spectra of **5** in CH_2Cl_2
Figure S20. Simulated and experimental UV-vis absorption spectra of **6** in CH_2Cl_2
Table S2. Excited states of **5** with oscillator strength (*f*) larger than 0.02
Table S3. Excited states of **6** with oscillator strength (*f*) larger than 0.02
Figure S21. Involved molecular orbitals of **5**
Figure S22. Involved molecular orbitals of **6**
Figure S23. MS spectra for complexation of **5** with $AgBF_4$ in CH_2Cl_2/CH_3CN (9:1, v:v)
Figure S24. UV-vis absorption spectra for complexation of **5** with $AgBF_4$ in CH_2Cl_2/CH_3CN (9:1, v:v)
Figure S25. Emission spectra for complexation of **5** with $AgBF_4$ in CH_2Cl_2/CH_3CN (9:1, v:v)
Figure S26. ^{1}H NMR spectra for complexation of **5** with $AgBF_4$ in CD_2Cl_2/CD_3CN (9:1, v:v)
Figure S27. ^{1}H NMR spectra for complexation of **6** with $AgBF_4$ in CD_2Cl_2/CD_3CN (9:1, v:v)
Figure S28. MS spectra for complexation of **6** with $AgBF_4$ in CH_2Cl_2/CH_3CN (9:1, v:v)
Figure S29. UV-vis absorption spectra for complexation of **6** with $AgBF_4$ in CH_2Cl_2/CH_3CN (9:1, v:v)
Figure S30. Emission spectra for complexation of **6** with $AgBF_4$ in CH_2Cl_2/CH_3CN (9:1, v:v)

Figure S31. MS spectra for complexation of **5** with $AgBF_4$ in CH_2Cl_2/CH_3OH (9:1, v:v)
Figure S32. MS spectra for complexation of **6** with $AgBF_4$ in CH_2Cl_2/CH_3OH (9:1, v:v)
Table S4. Key structural parameters for **2**-**4**, and some reported $CAu^I_6Ag^I_n$ clusters
Figure S33. ^{1}H NMR spectrum of **3** (500 MHz, CD_2Cl_2, 300 K)
Figure S34. ^{1}H-^{1}H COSY NMR spectrum of **3** (500 MHz, CD_2Cl_2, 300 K)
Figure S35. ^{13}C NMR spectrum of **3** (126 MHz, CD_2Cl_2, 300 K)
Figure S36. ^{1}H-^{13}C HSQC spectrum of **3** (500 MHz, CD_2Cl_2, 300 K)
Figure S37. Schematic illustration of intramolecular Au···H−C bonds in **2**-**4**
Figure S38. ^{1}H NMR spectrum of **3** (500 MHz, CD_2Cl_2/CD_3CN (9:1, v:v), 300 K)
Figure S39. UV-vis absorption spectrum of **3** in CH_2Cl_2
Figure S40. ESI MS spectra of **3**
Figure S41. ^{1}H NMR spectrum of **4** (500 MHz, CD_2Cl_2/CD_3CN (9:1, v:v), 300 K)
Figure S42. ^{1}H-^{1}H COSY NMR spectrum of **4** (500 MHz, CD_2Cl_2/CD_3CN (9:1, v:v), 300 K)
Figure S43. ^{13}C NMR spectrum of **4** (126 MHz, CD_2Cl_2/CD_3CN (9:1, v:v), 300 K)
Figure S44. ^{1}H-^{13}C HSQC spectrum of **4** (500 MHz, CD_2Cl_2/CD_3CN (9:1, v:v), 300 K)
Figure S45. ESI MS spectra of **4**
Figure S46. UV-vis absorption spectrum of **4** in CH_2Cl_2
Figure S47. Excitation and emission spectra of **3** in the solid state
Figure S48. Excitation and emission spectra of **4** in the solid state
Figure S49. Excitation and emission spectra of **3** in CH_2Cl_2
Figure S50. Excitation and emission spectra of **4** in CH_2Cl_2
Figure S51. UV-vis absorption spectra of **3** (a) and **4** (b) in CH_2Cl_2 that are kept under daylight at room temperature for one week to check their stability
Figure S52. UV-vis absorption, excitation, and emission spectra of **3** and **4** in CH_2Cl_2/CH_3CN (9:1, v:v) before and after stored at 4°C for 18 months
Figure S53. Optimized structures of **3** and **4**
Table S5. Bader charges and natural population analysis (NPA) charges of the central C, Au, and Ag atoms in **2**-**6**
Table S6. Excited states of **3** with oscillator strength (*f*) larger than 0.02
Figure S54. Involved molecular orbitals of **3**
Table S7. Excited states of **4** with oscillator strength (*f*) larger than 0.02
Figure S55. Involved molecular orbitals of **4**
Table S8. Orbital composition analysis with Mulliken partition of **2**-**6**
Figure S56. SOMO and SOMO-1 orbitals of **4**
Table S9. Phosphorescence energies of **2**-**4** and **4***
Table S10. Key structural parameters of **4***
Figure S57. Molecular structure of simulated **4***, and intramolecular Au···H−C bonds in **4***
Figure S58. Calculated UV-vis absorption spectra of cluster **4*** in CH_2Cl_2
Table S11. Calculated radiative rate constants of the low-lying spin-orbit states of **2**, **3**, and **4***
Table S12. Singlet and triplet components and spin-orbit coupling (SO) of the low-lying spin-orbit states of **2**, **3**, and **4***
10. References

1. Materials and reagents

All commercially available reagents were used as received. Benzimidazole, 2-bromopropane, potassium carbonate, 2-(chloromethyl)pyridine hydrochloride, sodium carbonate, anhydrous magnesium sulfate, silver oxide, chloroauric acid trihydrate, potassium hydroxide, sodium tetrafluoroborate, trimethylamine and all the dry solvents were purchased from Fujifilm Wako Pure Chemical Corporation. Tetrahydrothiophene (tht) and silver tetrafluoroborate were obtained from Tokyo Chemical Industry Co., LTD. Trimethylsilyldiazomethane (2.0 M in *n*-hexane) was bought from Sigma-Aldrich.

2. Physical measurements and instrumentation

NMR data were recorded on a Bruker Avance III spectrometer (500 MHz). When $CDCl_3$ was used, tetramethylsilane was used as the internal standard (0 ppm) for ^{1}H NMR. In the cases when CD_2Cl_2 was used, a mono-protonated solvent signal of CD_2Cl_2 ($CDHCl_2$, 5.32 ppm) and a solvent signal of CD_2Cl_2 ($^{13}CD_2Cl_2$, 53.84 ppm) were used as the internal standards for ^{1}H and ^{13}C NMR measuremants, respectively. Abbreviations: s, singlet; d, doublet; sept, septet; dd, double doublet; br, broad. ESI-TOF-MS spectrum were recorded on a Micromass LCT Premier XE. Experimental conditions: ion mode, positive; desolvation temperature, 150 °C; source temperature, 80 °C. UV-vis spectra were recorded on a Jasco V-770 spectrophotometer. Luminescence was measured on a Jasco FP-8300 spectrofluorometer. Luminescence quantum yields were determined on a Hamamatsu C9920-02G spectrometer. Luminescence lifetimes were determined on a Hamamatsu C11367-02 spectrometer. Elemental analysis was conducted in the Microanalytical Laboratory, Department of Chemistry, Graduated School of Science, the University of Tokyo.

3. Synthesis and characterizations

$[(C)(Au\text{-}\mathbf{BIPy})_6](BF_4)_2$ (**BIPy** = *N*-isopropyl-*N′*-2-pyridylbenzimidazolylidene) and $[(C)(Au\text{-}\mathbf{BIPy})_6Ag_2](BF_4)_4$ (**2**) were synthesized according to the literature procedures.[1]

3.1 **BIPc**·HCl (*N*-isopropyl-*N′*-2-picolylbenzimidazolium hydrochloride) and **BIPc²·**HCl (*N*,*N′*-Di-2-picolylbenzimidazolium hydrochloride)

BIPc·HCl was synthesized according to the literature procedures with modifications.[2,3]

Under an atmosphere of nitrogen, a mixture of benzimidazole (1.18 g, 10 mmol) and potassium carbonate (1.52 g, 11 mmol) was suspended in CH_3CN (30 mL) in a Schlenk flask and stirred at ambient temperature for 1 h. Then, isopropyl bromide (2.2 mL, 20 mmol) was added into the suspension and the reaction mixture was heated at reflux for 36 h. After the solvent was removed under vacuum the residue extracted with H_2O/CH_2Cl_2 (20:15 mL). Removal of the solvent from the organic layer gave 1-isopropylbenzimidazole as an oil. The resulting oil, 2-(chloromethyl)pyridine hydrochloride (1.64 g, 10 mmol) and potassium carbonate (2.76 g, 20 mmol) were then suspended in CH_3CN (50 mL) in a Schlenk flask under nitrogen atmosphere. This mixture was heated at reflux for 48 h. The solvent was then removed in vacuo, and the residue was dissolved in CH_2Cl_2 (50 mL). Filtration and removal of the solvent from the filtrate yielded a viscous residue. Colorless block-like crystals of **BIPc**·HCl were obtained by layering Et_2O on a filtered CH_2Cl_2/CH_3OH (9:1, v:v) solution of product in tubes. Yield: 1.30 g (45%, based on benzimidazole).

^{1}H NMR (500 MHz, $CDCl_3$, 300 K, ppm): δ 12.12 (s, 1H), 8.50 (d, *J* = 4.1 Hz, 1H), 8.08–8.05 (m, 2H), 7.75 (td, *J* = 7.7, 1.7 Hz, 1H), 7.70–7.68 (m, 1H), 7.61–7.59 (m, 2H), 7.24 (dd, *J* = 6.9, 5.0 Hz, 1H), 6.13 (s, 2H), 4.93 (sept, *J* = 6.7 Hz, 1H), 1.84 (d, *J* = 6.7 Hz, 6H).

BIPc²·HCl was synthesized according to the literature procedures.[4] Crystallization was performed by layering Et_2O on a filtered CH_2Cl_2/CH_3OH (9:1, v:v) solution of **BIPc²**·HCl in tubes.

3.2 Homometallic CAu^I_6 clusters $[(C)(Au^I\text{-}\mathbf{BIPc})_6](BF_4)_2$ (**5**) and $[(C)(Au^I\text{-}\mathbf{BIPc^2})_6](BF_4)_2$ (**6**)

Complexes [(C)(Au^I-**BIPc**)$_6$](BF$_4$)$_2$ (**5**) and [(C)(Au^I-**BIPc^2**)$_6$](BF$_4$)$_2$ (**6**) were synthesized according to the literature procedures with modifications.[1,5,6]

Ag_2O (35.0 mg, 0.15 mmol) was added to a solution of benzimidazolium chloride (**BIPc**·HCl (80.4 mg, 0.30 mmol) for **5**; **BIPc^2**·HCl (98.7 mg, 0.30 mmol) for **6**) in CH_2Cl_2/CH_3OH (7.5 mL/2.5 mL). The suspension was stirred for 2.5 h in the dark and then filtered through Celite. After tht-AuCl (96.0 mg, 0.30 mmol) was added, the solution was stirred overnight (~12 h) in the dark. The suspension was again filtered through Celite, and the solvents were then removed using a rotary evaporator. After adding $NaBF_4$ (165 mg, 1.5 mmol) and CH_3OH (5 mL), the suspension was stirred for 5 min. CH_2Cl_2 (15 mL), a solution of KOH (28.0 mg, 0.50 mmol) in CH_3OH (3 mL), a solution of $AgBF_4$ (58.5 mg, 0.30 mmol) in CH_3OH (1 mL), and H_2O (50 µL) were then sequentially dropwise added to the mixture under stirring, which leads to a brown suspension. After another stirring for 5 min, the suspension was again filtered through Celite and evaporated to dryness. The solid was then transferred to a Schlenk flask with nitrogen atmosphere, and dry CH_2Cl_2 (5 mL), Et_3N (30.0 µL, 0.20 mmol) and a 2.0 M solution of Me_3SiCHN_2 in *n*-hexane (48.0 µL, 0.10 mmol) were added. The resulting mixture was stirred for another 1 h. After filtration into a tube, a layer of dry Et_2O was added on the CH_2Cl_2 solution, which gave the products as colorless block-like crystals. Yields: 66.1 mg (46%, based on tht-AuCl) for **5**; 68.1 mg (43%, based on tht-AuCl) for **6**.

For **5**: Anal. Calcd for $C_{97}H_{102}B_2N_{18}F_8Au_6{\cdot}CH_2Cl_2$: C, 39.76; H, 3.54; N, 8.52. Found: C, 39.46; H, 3.70; N, 8.46. ^{1}H NMR (500 MHz, CD_2Cl_2, 300 K, ppm): δ 7.66 (d, *J* = 4.4 Hz, 1H), 7.61 (d, *J* = 8.3 Hz, 1H), 7.44 (t, *J* = 7.7 Hz, 1H), 7.31 (q, *J* = 8.3 Hz, 2H), 7.10 (d, *J* = 8.1 Hz, 1H), 6.88 (dd, *J* = 7.2, 5.0 Hz, 1H), 6.68 (d, *J* = 7.8 Hz, 1H), 5.77 (s, 2H), 5.28 (sept, *J* = 6.9 Hz, 1H), 1.67 (d, *J* = 6.9 Hz, 6H). ^{13}C NMR (126 MHz, CD_2Cl_2, 300 K, ppm): δ 188.3, 155.2, 149.6, 137.1, 133.4, 132.6, 124.86, 124.76, 123.0, 120.0, 112.66, 112.64, 53.9, 52.5, 22.8. MS (ESI-TOF, positive-mode, solvent: CH_2Cl_2) *m/z* calcd. for [(C)(Au-**BIPc**)$_6$]$^{2+}$: 1350.3; found: 1350.2.

For **6**: Anal. Calcd for $C_{115}H_{96}B_2N_{24}F_8Au_6$: C, 43.58; H, 3.05; N, 10.61. Found: C, 43.90; H, 3.35; N, 10.54. ^{1}H NMR (500 MHz, CD_2Cl_2, 300 K, ppm): δ 8.08 (d, *J* = 4.0 Hz, 1H), 7.33 (d, *J* = 1.5 Hz, 2H), 7.19 (d, *J* = 7.8 Hz, 1H), 7.09 (td, *J* = 7.7, 1.7 Hz, 1H), 6.81 (dd, *J* = 7.1, 5.1 Hz, 1H), 5.63 (s, 2H). ^{13}C NMR (126 MHz, CD_2Cl_2, 300 K, ppm): δ 190.7, 155.0, 149.6, 137.2, 133.4, 125.0, 123.3, 121.9, 112.8, 53.7. MS (ESI-TOF, positive-mode, solvent: CH_2Cl_2) *m/z* calcd. for [(C)(Au-**BIPc^2**)$_6$]$^{2+}$: 1497.3; found: 1497.3.

3.3 Complexation experiments of **5** and **6** with $AgBF_4$

For MS, UV-vis, and luminescence monitoring of complexation of **5** and **6** with $AgBF_4$, 3.0 mL solutions (CH_2Cl_2/CH_3CN (9:1, v:v) or CH_2Cl_2/CH_3OH (9:1, v:v)) containing 5.0×10^{-5} mmol CAu$_6$ and different equiv. of $AgBF_4$ were prepared. UV-vis, luminescence, photo of luminescence, and MS spectra were then sequentially measured or taken. For ^{1}H NMR, solutions of **5** or **6** (1.0×10^{-3} mmol, 400 µL) in CD_2Cl_2/CD_3CN (9:1, v:v) were first prepared in an NMR tube and measured. A solution of $AgBF_4$ (1.0×10^{-1} mol/L, 10.0 µL) in CD_3CN and 40 µL of CD_2Cl_2 were added in turn to each sample in the tube and measured again. This was repeated for 5 times.

3.4 Heterometallic CAu$^I_6$Ag$^I_3$ cluster [(C)(Au^I-**BIPc**)$_6$Ag$_3$(CH$_3$CN)$_3$](BF$_4$)$_5$ (**3**) and CAu$^I_6$Ag$^I_4$ cluster [(C)(Au^I-**BIPc^2**)$_6$Ag$_4$(CH$_3$CN)$_2$](BF$_4$)$_6$ (**4**)

Complexes [(C)(Au^I-**BIPc**)$_6$Ag$_3$(CH$_3$CN)$_3$](BF$_4$)$_5$ (**3**) and [(C)(Au^I-**BIPc^2**)$_6$Ag$_4$(CH$_3$CN)$_2$](BF$_4$)$_6$ (**4**) were synthesized according to the literature procedures with modifications.[1,7–9]

CAu$^I_6$ cluster (**5** (28.8 mg, 10 µmol) for **3**; **6** (31.7 mg, 10 µmol) for **4**) was dissolved in dry CH_2Cl_2 (4.5 mL). $AgBF_4$ (8.0 mg, 40 µmol for **3**; 10.0 mg, 50 µmol for **4**) in dry CH_3CN (0.5 mL) was added to the solution under stirring. The mixture was then filtered and the filtrate was layered with dry Et_2O. Orange crystals can be obtained within one week. Yield: 27.3 mg (76%, based on **5**) for **3**; 36.0 mg (89%, based on **6**) for **4**.

For **3**: Anal. Calcd for $C_{97}H_{102}B_5N_{18}F_{20}Ag_3Au_6{\cdot}2CH_2Cl_2$: C, 32.76; H, 2.94; N, 6.95. Found: C, 32.53; H, 3.20; N, 7.12. ^{1}H NMR (500 MHz, CD_2Cl_2, 300 K, ppm): δ 8.37 (d, *J* = 7.6 Hz, 1H), 7.60–7.57 (m, 1H), 7.54 (d, *J* = 6.1 Hz, 1H), 7.45 (s, 1H), 7.40 (d, *J* = 3.4 Hz, 3H), 6.79–6.64 (m, 2H), 6.10 (d, *J* = 14.5 Hz, 1H), 5.03 (t, *J* = 2.6 Hz, 1H). ^{13}C NMR (126 MHz, CD_2Cl_2, 300 K, ppm): δ 152.6, 151.6, 140.4, 133.3, 128.5, 126.09, 125.95, 125.80, 124.9, 113.08, 113.00, 54.9, 53.47, 23.6. MS (ESI-TOF, positive-mode, solvent: CH_2Cl_2) *m/z* calcd. for [(C)(Au-**BIPc**)$_6$Ag$_2$](BF$_4$)$_3^+$: 3175.5; found: 3175.5. Quantum yield (r.t.): 0.04 in CH_2Cl_2, 0.01 in CH_2Cl_2/CH_3CN = 9:1 (v:v). Lifetime (r.t.): 0.23 µs in CH_2Cl_2.

For **4**: Anal. Calcd for $C_{115}H_{96}B_6N_{24}F_{24}Ag_4Au_6{\cdot}3CH_2Cl_2$: C, 33.72; H, 2.45; N, 8.00. Found: C, 33.61; H, 2.80; N, 8.19. ^{1}H NMR (500 MHz, CD_2Cl_2/CD_3CN = 9:1 (v:v), 300 K, ppm): δ 8.03 (d, *J* = 6.2 Hz, 1H), 7.78 (t, *J* = 7.4 Hz, 1H), 7.35–7.26 (m, 3H), 6.57 (s, 1H), 6.06 (d, *J* = 14.1 Hz, 1H), 5.38 (d, *J* = 15.2 Hz, 1H). ^{13}C NMR (126 MHz, CD_2Cl_2/CD_3CN =

9:1 (v:v), 300 K, ppm): δ 153.0, 150.1, 140.1, 132.4, 128.0, 126.3, 125.0, 112.6, 54.6. MS (ESI-TOF, positive-mode, solvent: CH_2Cl_2/CH_3CN = 9:1 (v:v)) *m/z* calcd. for $[(C)(Au\text{-}\mathbf{BIPc^2})_6Ag_4](BF_4)_4^{2+}$: 1885.1; found: 1885.1. Quantum yield (r.t.): 0.40 in CH_2Cl_2, 0.09 in CH_2Cl_2/CH_3CN = 9:1 (v:v). Lifetime (r.t.): 1.41 μs in CH_2Cl_2.

4. X-ray crystallography

Intensity data of compounds **BIPc**·HCl, **BIPc²**·HCl, $[(C)(Au^I\text{-}\mathbf{BIPc})_6](BF_4)_2$ (**5**), $[(C)(Au^I\text{-}\mathbf{BIPc^2})_6](BF_4)_2$ (**6**), $[(C)(Au^I\text{-}\mathbf{BIPc})_6Ag_3(CH_3CN)_3](BF_4)_5$ (**3**), and $[(C)(Au^I\text{-}\mathbf{BIPc^2})_6Ag_4(CH_3CN)_2](BF_4)_6$ (**4**) were collected on a Rigaku XtaLAB Synergy-DW system (CuKα) at 93 K. The structures were solved by direct methods, and non-hydrogen atoms except for the disordered BF_4^- in **6** and the central carbon atom in **4** were refined anisotropically by the least-squares on F^2 using the SHELXTL program. As a result, **4** has one level B alert (Isotropic non-H Atoms in Main Residue). The hydrogen atoms of organic ligands were generated geometrically. Squeeze tool of PLATON and absorption correction using WinGX were applied to **3**, due to the large solvent voids and heavy absorption.

5. Computation details

DFT and TD-DFT calculations were performed for all clusters using the B3LYP functional.[10] Relativistic effective core potential LANL2DZ[11] was used for Au and Ag atoms and the basis sets of other atoms were 6-31G*.[12] Optimizations were carried out based on the crystal structures and vibrational frequency analyses were conducted to verify the stationary points to be local minima on the potential energy surface. **4*** was constructed by removing the two acetonitrile groups of **4**, and a highly symmetric structure was obtained after optimization.

For simulating absorption spectra, 100 excited states were solved to cover the spectrum in the energy range up to about 200 nm and the rotatory strength was calculated in the velocity form. TD-DFT calculation was conducted including the solvent effects of CH_2Cl_2 with the polarizable continuum model (PCM) and the non-equilibrium linear response scheme.[13] To calculate phosphorescence energies, we obtained the optimal geometry of the lowest triplet excited state (T_1) and, at this geometry, calculated the emission energy with the Δ self-consistent-field (ΔSCF) approach. All calculations were conducted using the Gaussian 16 suite of programs.[14]

The natural population analysis (NPA) charges were calculated at the level of B3LYP/6-31G* using NBO 3.1 as implemented in Gaussian 16.[14] Bader charges and orbital compositions were obtained by the Multiwfn program.[15]

The radiative rate constants of **3** and **4*** by ZORA method including spin-orbit interaction in the perturbative method[16] implemented in ADF program package.[17] The B3LYP functional combined with DZ basis set was utilized.

Note that some metal-metal bonds of the triplet structure of **3** are extraordinarily elongated (dissociated) during optimization in the gas phase, which provides the problem for DFT calculations with a single determinant. Therefore, the lowest triplet excited state was obtained by optimization including the solvent effects. Meanwhile, it is practically impossible to locate MECP of S0/T1, which makes the discussion of non-radiative decay difficult theoretically.

6. Additional results and discussion on the homometallic CAu^I_6 clusters 5 and 6

Single crystal X-ray diffraction (ScXRD) analysis was successfully performed on the hexagold(I) clusters $[(C)(Au^I\text{-}\mathbf{BIPc})_6](BF_4)_2$ (**5**) and $[(C)(Au^I\text{-}\mathbf{BIPc^2})_6](BF_4)_2$ (**6**). As shown in Fig. S2, both of them have an octahedral structure with a carbon tetraanion in the center. Each NHC ligand **BIPc** or **BIPc²** in the complexes coordinates to one gold atom. The Au-Au distances in **6** were found in a range from 2.8585(3) to 3.1911(3) Å, which shows slight larger deviation than other known hexagold(I) clusters (Table S1).[1,5,6,17] As a result, the arrangement of ligands **BIPc²** in **6**, especially their wingtip groups, are less ordered. Other key structure parameters such as Au-C (center) and Au-C (ligand) distances in **5** and **6** are comparable with known NHC-protected CAu^I_6 clusters.

Global characterizations were performed on **5** and **6**, including NMR, UV-vis, ESI MS, luminescence etc (Figures S4–17). First, there is only one set of signals in the NMR spectra of both compounds. That is to say, the six Au^+-**BIPc** or Au^+-**BIPc²** moieties are equivalent when the clusters are dissolved in solution. The slightly altered octahedral structure of **6**, as indicated by ScXRD, is probably due to the bulkiness and large number of picolyl wingtip groups, which affected the packing of molecules when forming crystals. Second, both **5** and **6** have triplet absorption peaks at around 350 nm in CH_2Cl_2, which are very similar to reported hexagold(I) clusters protected by NHC ligands.[1,5,6] Metal-to-ligand charge transfer (MLCT) from the metal kernel to NHC ligands is a highly possible origin. Third, sharp peaks correspond to $[(C)(Au^I\text{-}\mathbf{BIPc})_6]^{2+}$ and $[(C)(Au^I\text{-}\mathbf{BIPc^2})_6]^{2+}$ etc. were observed in the mass

spectra. The experimental and simulated pattern fit well with each other. Last but not the least, both **5** and **6** show green luminescence in the solid state, which is similar to $[(C)(Au^I\text{-BI}i\text{Pr})_6](BF_4)_2$ but very different from $[(C)(Au^I$-**IPy**$)_6](BF_4)_2$ and $[(C)(Au^I$-**BIPy**$)_6](BF_4)_2$ (Figure S1).[1,6] Previously, we found that the phenyl part of the **BI*i*Pr** in $[(C)(Au^I$-**BI*i*Pr**$)_6](BF_4)_2$ is significantly involved in the lowest unoccupied molecular orbital (LUMO). However, the installation of aromatic wingtip group (pyridyl) will dramatically change the electronic structure of cluster. The pyridyl groups, instead of the phenyl parts in $[(C)(Au^I$-**BIPy**$)_6](BF_4)_2$, primarily participate in the LUMOs of cluster. As a result, the luminescence shows obvious blue-shift. In the cases of **5** and **6**, the emissions were restored to green. This suggests that the introduction of methylene linker between benzimidazolyl and pyridyl groups may efficiently decouple the electron resonance.

To try to explain the UV-vis absorption and luminescence of **5** and **6**, time-dependent density functional theory (TD-DFT) calculations were carried out. The absorption spectra of **5** and **6** were theoretically simulated as shown in Figures S19 and S20, respectively, which well reproduced the experimental ones in CH_2Cl_2. It is confirmed that the lowest peaks around 350 nm are mainly attributed to the MLCT transition from the Au kernel to ligands, while the strong peaks around 260 nm mainly come from the $\pi\pi^*$ transitions of the ligands. (Tables S2 and S3) In addition, the involved molecular orbitals of **5** and **6** are illustrated in Figures S21 and S22. Indeed, the HOMOs (highest occupied molecular orbitals) are mainly located in the CAu^I_6 cores of clusters, and more importantly, the HOMOs are located in the benzimidazolylidene moieties of NHC ligands, with almost no contribution from picolyl groups. These results indicate that electronic structures of the whole cluster can be tuned by employing alkyl or aromatic wingtip groups.

7. Additional results and discussion on the complexation experiments of 5 and 6 with $AgBF_4$ in CH_2Cl_2/CH_3OH (9:1, v:v)

In our first trials, the complexations of **5** and **6** with $AgBF_4$ were conducted in CH_2Cl_2/CH_3OH (9:1, v:v), and the complexation processes were monitored by using ESI MS. As shown in Figure S31, after adding 1 equiv. of $AgBF_4$ into **5** in a mixed solution of CH_2Cl_2/CH_3OH (v:v = 9:1), the peaks correspond to **5** almost immediately disappeared. One set of heterometallic peaks including $[(C)(Au^I$-**BIPc**$)_6Ag^I](BF_4)^{2+}$, $[(C)(Au^I$-**BIPc**$)_6Ag^I_2](BF_4)_2^{2+}$ and $[(C)(Au^I$-**BIPc**$)_6Ag^I_3](BF_4)_3^{2+}$ emerged, as well as a peak around m/z = 1094.5. This peak became dominant when the amount of $AgBF_4$ was further increased. According to the isotope pattern, it can be concluded that this mono cationic peak contains no Ag^+ or BF_4^-. We assign this peak to $[HAu_2$(**BIPc**)(picolylbenzimidazolylidene)$_2(CH_3OH)]^+$, which may contain protic NHCs and is rather abnormal. Although ionization conditions of ESI MS may lead to dissociation of cluster, such fragmentation of NHC ligands indicates potential decomposition of heterometallic cluster with the presence of both CH_3OH and silver ions.

The complexation of **6** and $AgBF_4$ was also conducted in CH_2Cl_2/CH_3OH (Figure S32). Similar to that of **5**, bright luminescence was observed when the secondary ions were added. In addition, a strong peak containing a decomposed ligand was also detected in the ESI MS spectra. Note that the difference between this monocationic peak (m/z 1143.5) and the signal observed in the complexation of **5** and $AgBF_4$ (m/z 1094.5) is ~49, which is exactly the difference between ligands **BIPc** and **BIPc²**. Considering the monocationic nature and large mass-to-charge ratio of these two peaks, more than one organic ligand or motif should be included. This further backed up our presumption that protic ligands were formed during the ionization process with the presence of CH_3OH and silver ions. Similarly, no heterometallic product was isolated under this condition.

8. Additional results and discussion on the theoretical calculation of phosphorescence lifetimes and radiative rate constants of 3 and $[(C)(Au^I\text{-BIPc}^2)_6Ag^I_4](BF_4)_6$ (4*)

Except for the absorption and phosphorescence energy, we have also evaluated the phosphorescence lifetimes and the radiative rate constants of **3** and **4*** using the ZORA method with spin-orbit interaction in a perturbative way implemented in ADF program package. The results were compared with that of **2**. The calculated k_r is obtained as $k_r = 1/\tau$.

As collected in Table S9, the three lowest-lying spin-orbit states of **2**, **3** and **4*** are degenerate with rather close energies but different oscillator strength values (f) and phosphorescence lifetimes (τ), which mainly contributes to the phosphorescence. The lifetime of the spin-orbit state with the largest f value is 27.97 μs for **3**, which is smaller than the corresponding values of **2** (35.25 μs) and **4*** (63.10 μs). Thus, the trend of lifetime is partly consistent with that observed in experiments. The calculated k_r values show a weak correlation with the

experimental ones. The combination of several states may contribute to the observed phosphorescence, resulting in the disagreement in the experiment and theory.

The wavefunction and spin-orbit coupling of the low-lying spin-orbit states were also analyzed (Table S10). These states are mainly composed of T_1 or S_1, with small contributions from other excited states. It can be seen that the Ag atoms coordinated to the Au kernel significantly changed the main component of each state and the coupling of the spin-orbit states, leading to the different k_r values of these compounds.

9. Supplementary schemes, figures, and tables

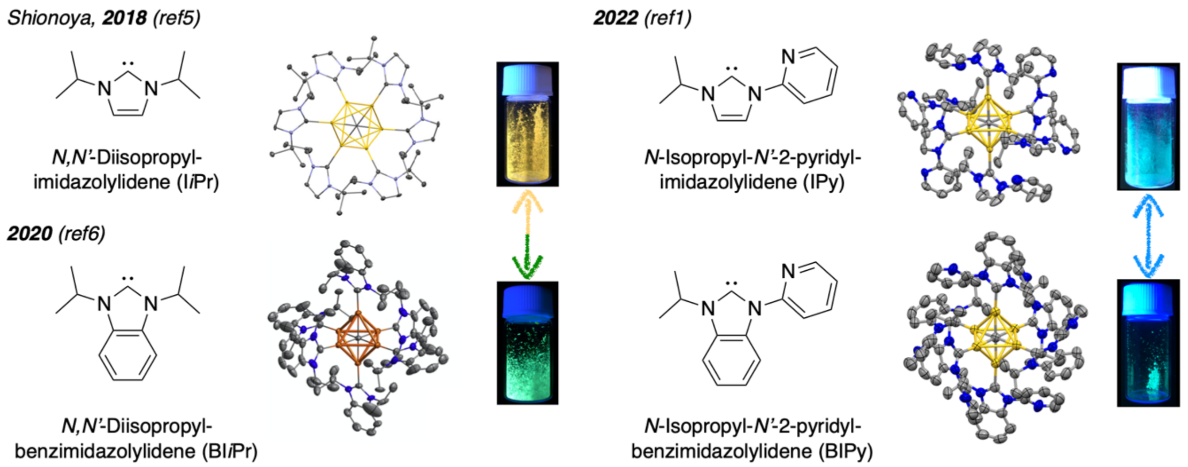

Scheme S1. Aromatic wingtip groups of (benz)imidazolylidene may significantly contribute to the photophysical properties of the corresponding metal clusters

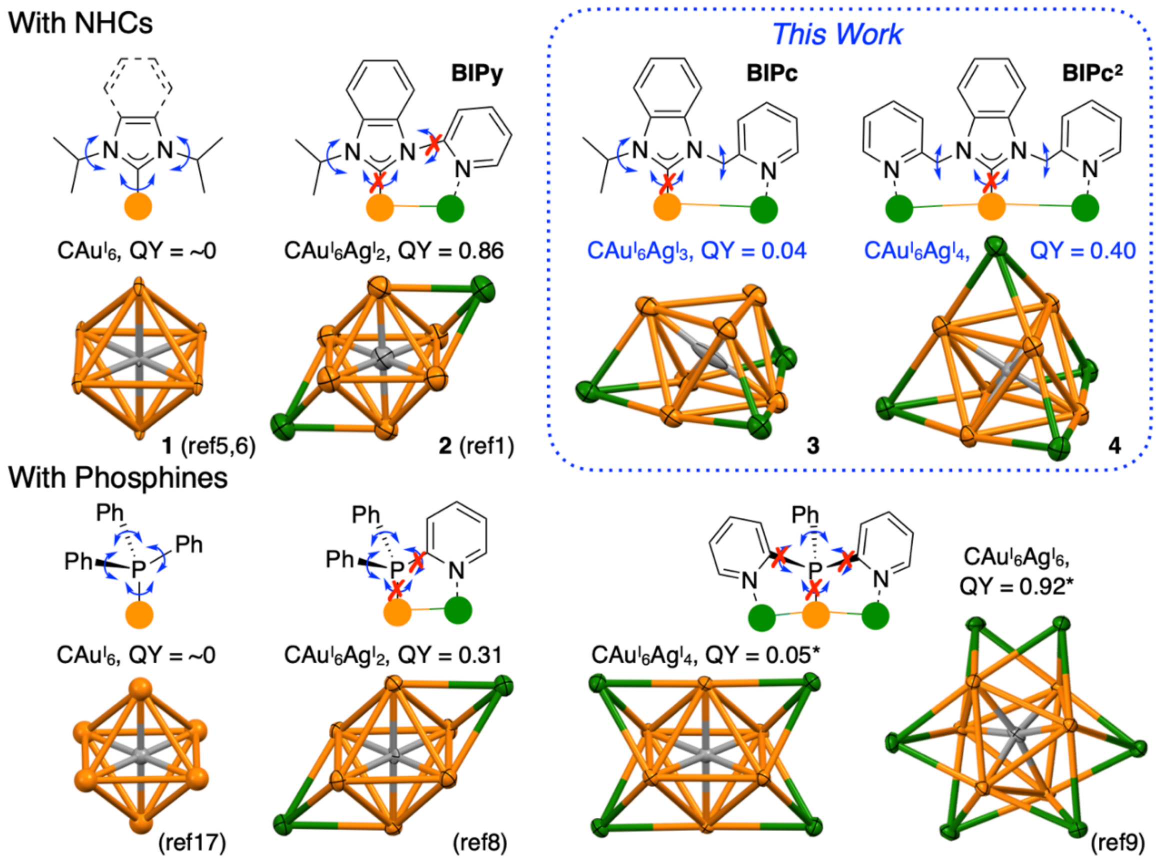

Scheme S2. Coordination modes of ligands, structures of metal kernels, and QYs (in CH_2Cl_2) of CAu^I_6-based clusters protected by NHC and phosphine ligands

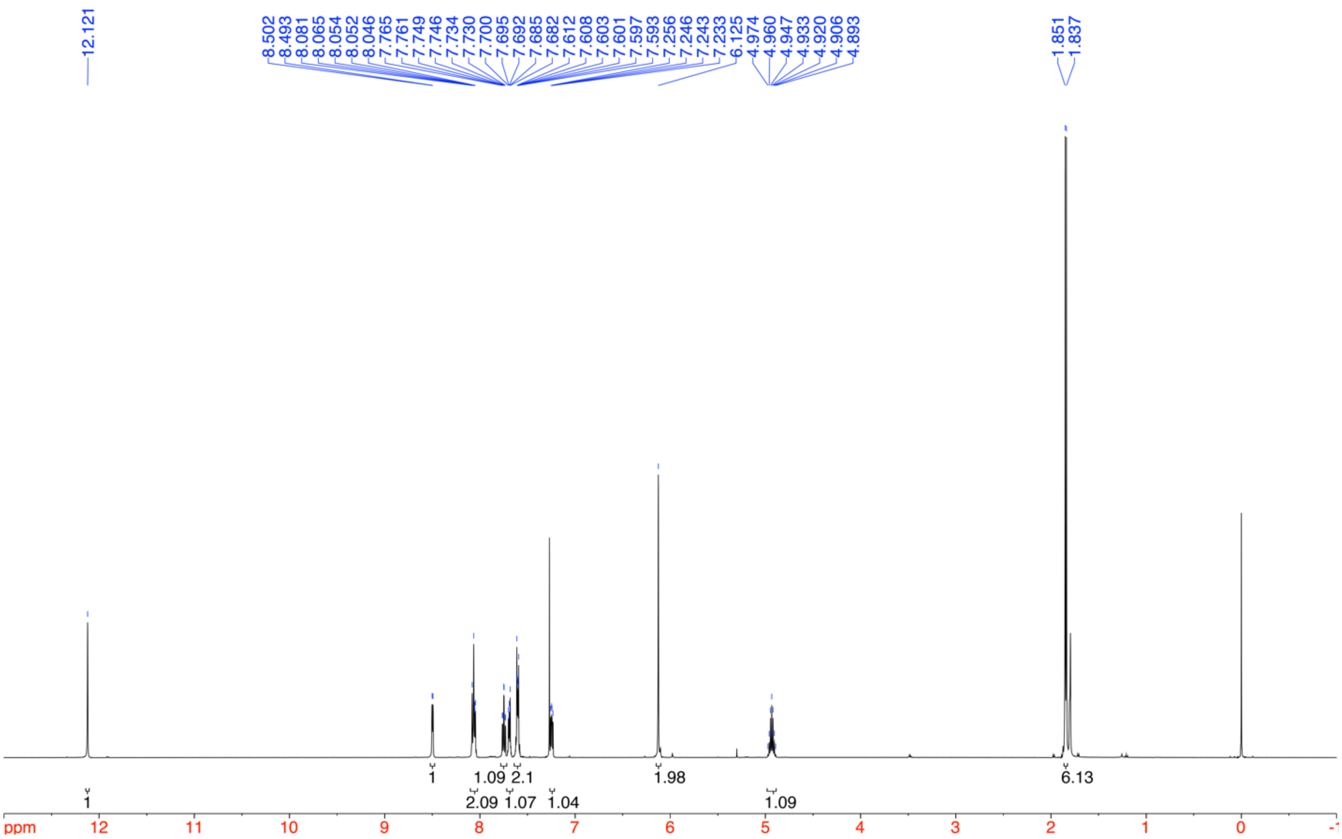

Figure S1. ^{1}H NMR spectrum of **BIPc**·HCl (500 MHz, $CDCl_3$, 300 K)

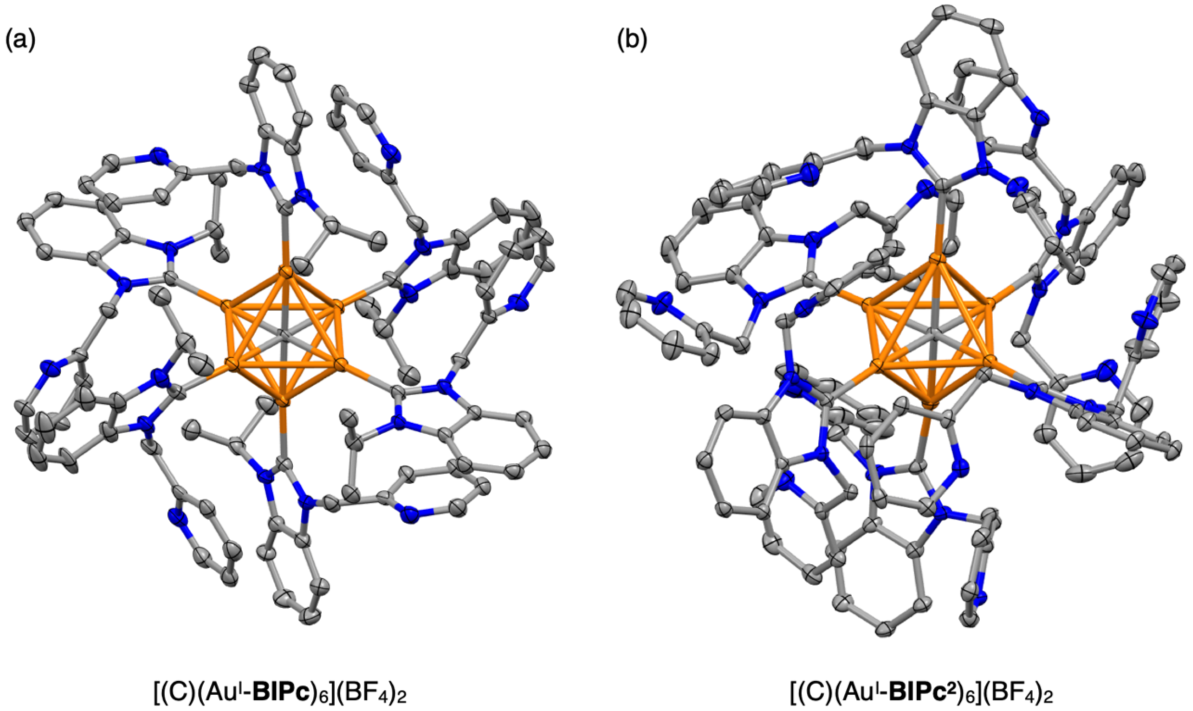

Figure S2. Molecular structures of **5** and **6**

Table S1. Key structural parameters of **5**, **6** and some reported CAu^{I}_{6} clusters (*Corresponds to Au–P distances for $[(C)(Au\text{-}PPh_3)_6](BF_4)_2$)

	**Au–C (central C^{4-}, Å)**	**Au–Au (Å)**	**Au–C (NHC ligands, Å)***
[(C)(Au-**BIPy**)$_6$](BF$_4$)$_2$[1]	2.1164(4)	2.9807(7)–3.0055(8)	2.046(11)
[(C)(Au^I-**BIPc**)$_6$](BF$_4$)$_2$ (**5**)	2.1086(4)–2.1107(3)	2.9350(5)–3.0339(5)	2.024(9)–2.039(9)
[(C)(Au^I-**BIPc^2**)$_6$](BF$_4$)$_2$ (**6**)	2.097(6)–2.138(6)	2.8585(3)–3.1911(3)	2.008(6)–2.030(6)
[(C)(Au-**I*i*Pr**)$_6$](BF$_4$)$_2$[5]	2.1158(3)	2.9282(3)–3.0548(3)	2.020(5)
[(C)(Au-**BI*i*Pr**)$_6$](BF$_4$)$_2$ (**1**)[6]	2.1142(2)–2.11580(18)	2.9529(3)–3.0280(3)	2.026(5)–2.038(5)
[(C)(Au-PPh$_3$)$_6$](BF$_4$)$_2$[18]	2.09(1)–2.15(1)	2.887(1)–3.226(1)	2.254(4)–2.277(4)

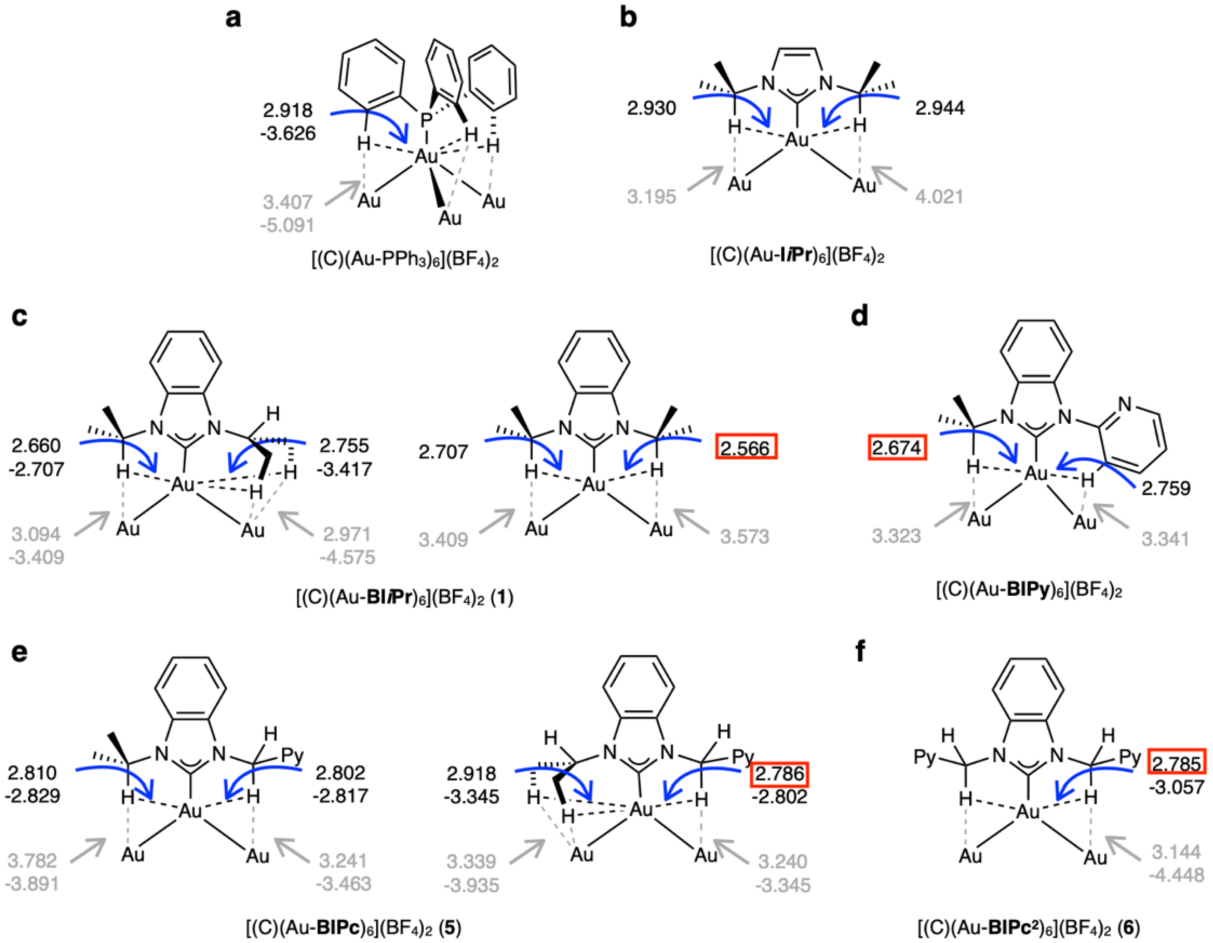

Figure S3. Schematic illustration of intramolecular Au···H–C bonds in **5**, **6**, and some reported CAu^{I}_{6} clusters

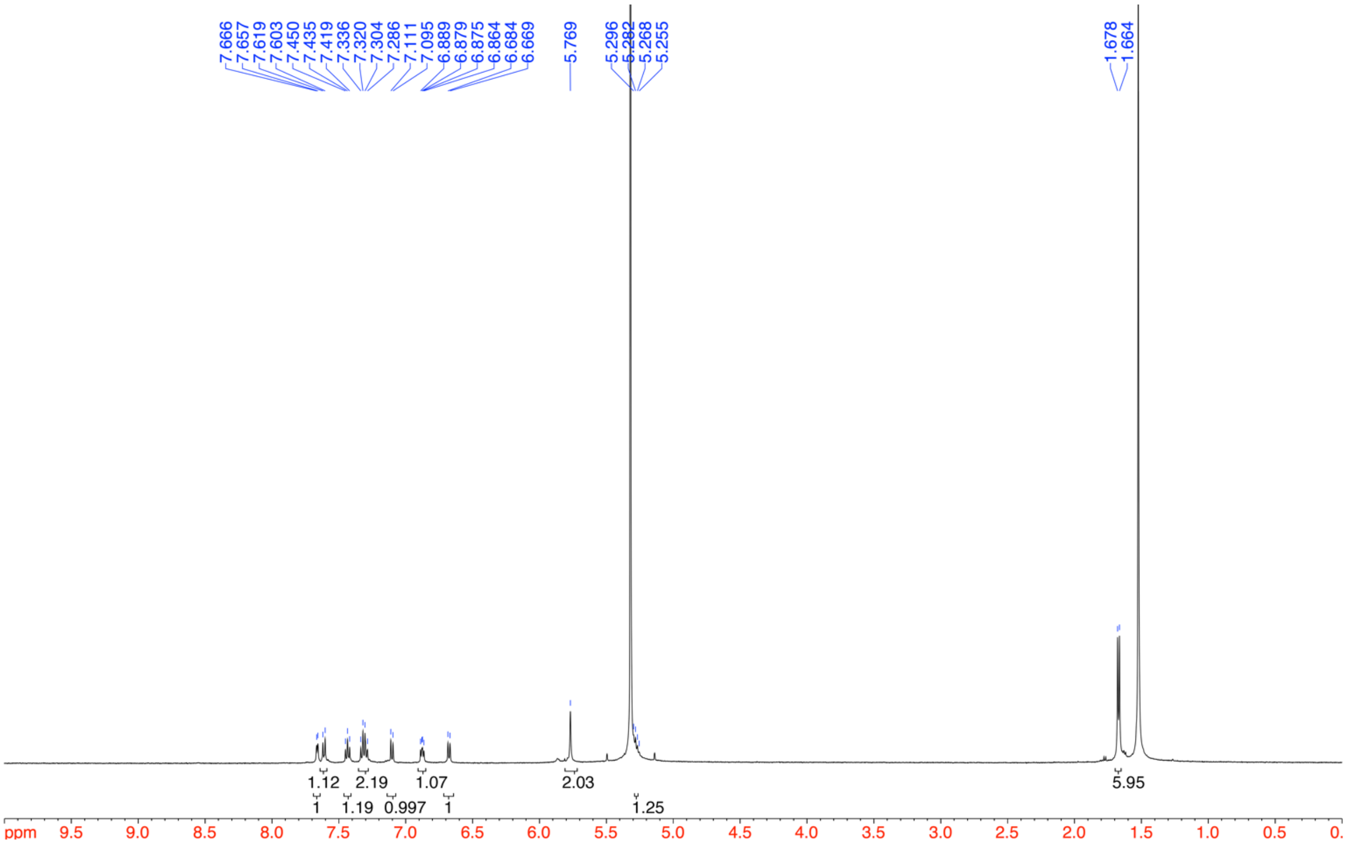

Figure S4. ^{1}H NMR spectrum of **5** (500 MHz, CD_2Cl_2, 300 K)

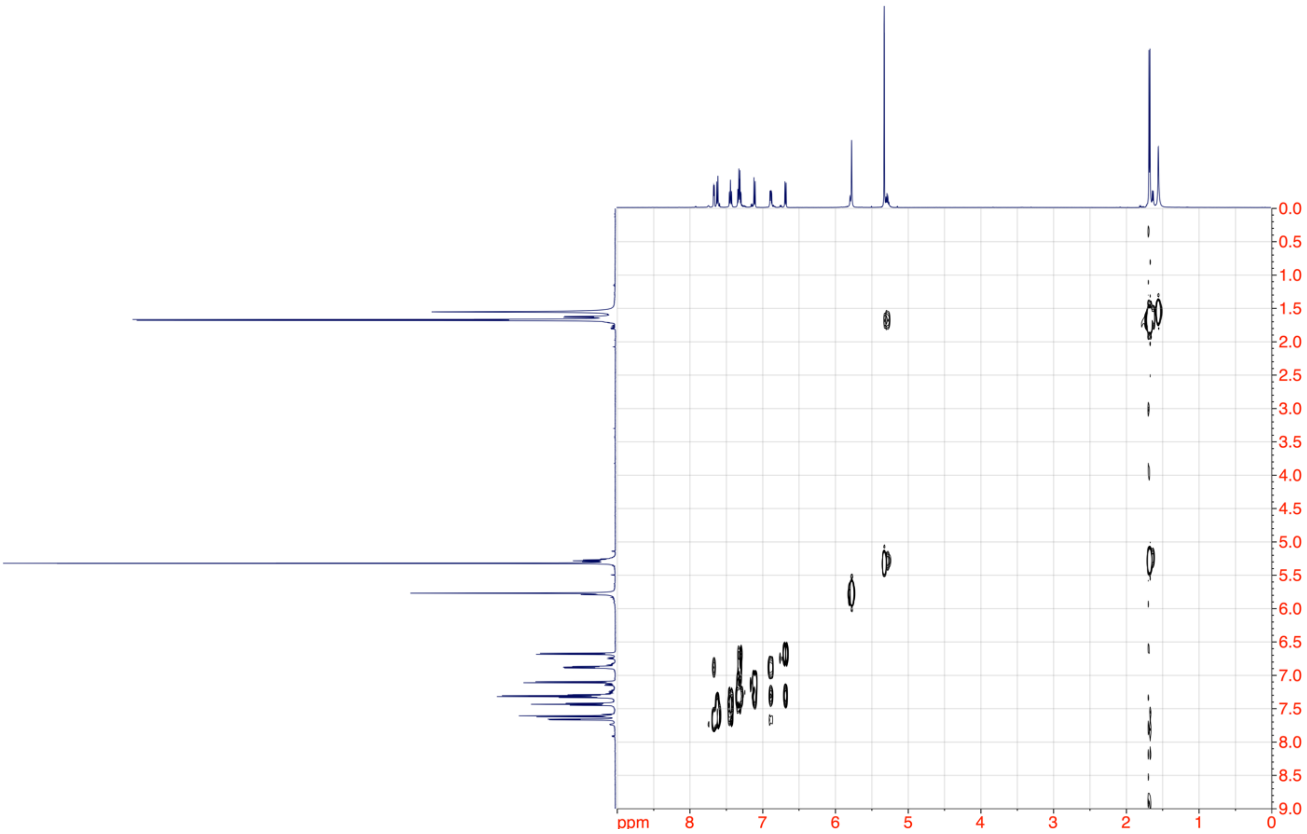

Figure S5. ^{1}H-^{1}H COSY NMR spectrum of **5** (500 MHz, CD_2Cl_2, 300 K)

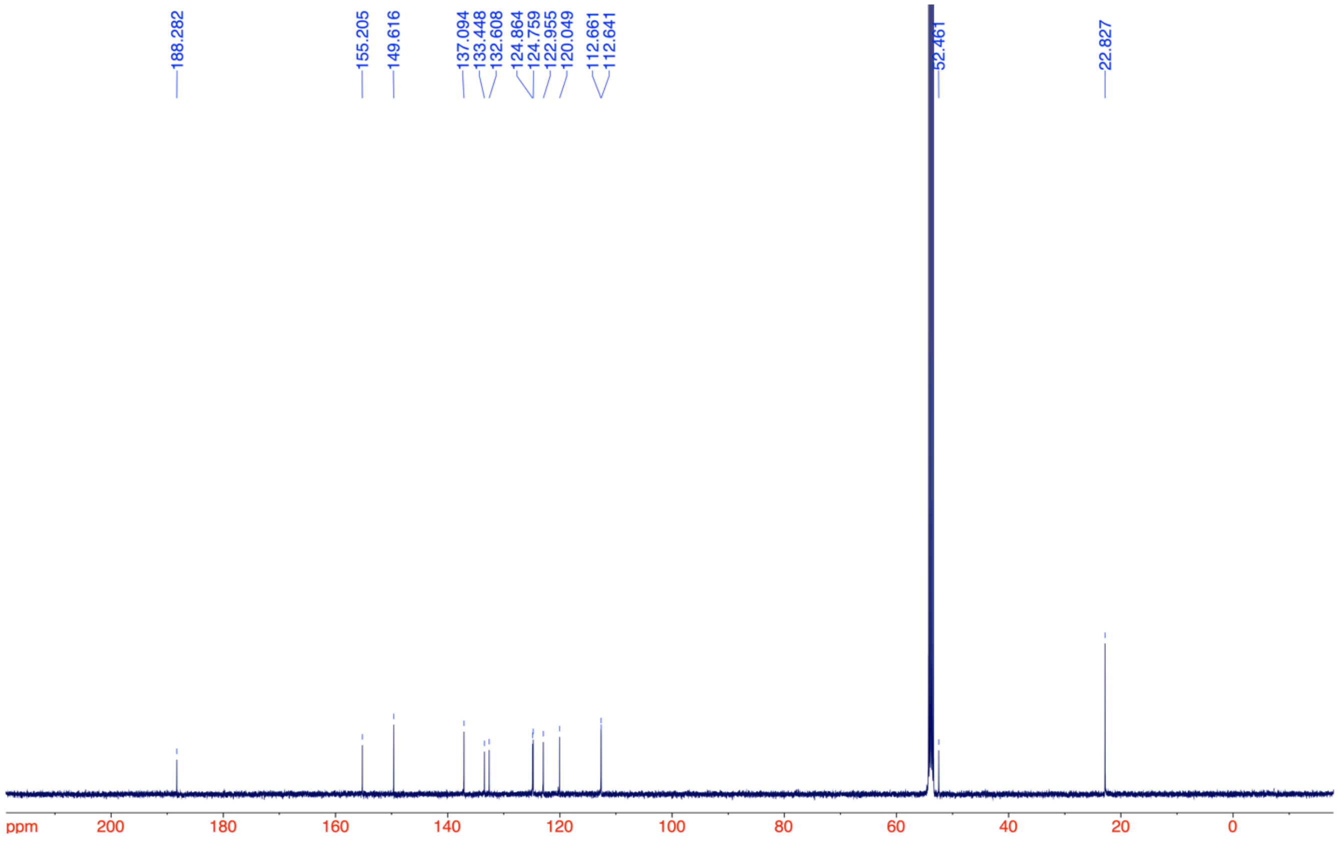

Figure S6. ^{13}C NMR spectrum of **5** (126 MHz, CD_2Cl_2, 300 K)

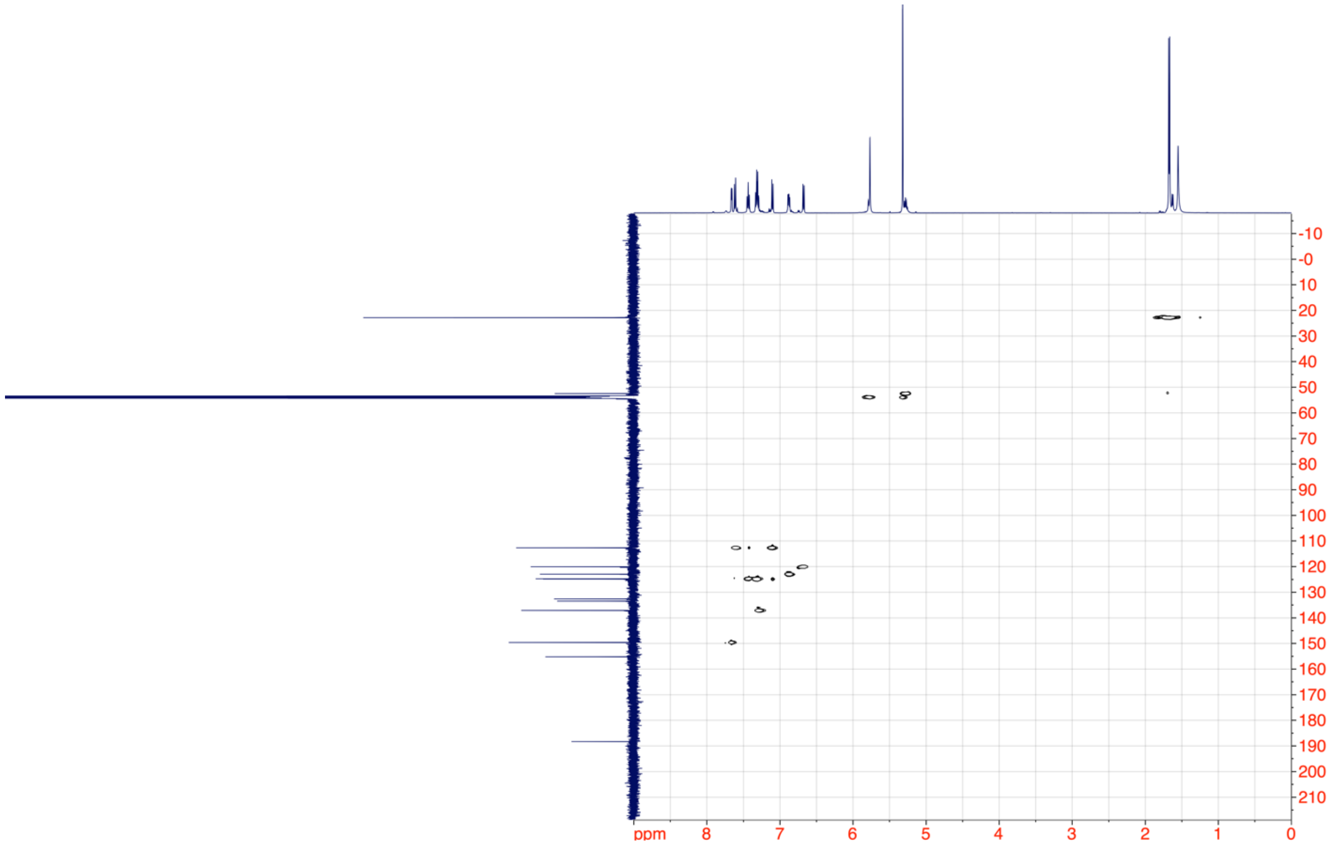

Figure S7. ^{1}H-^{13}C HSQC spectrum of **5** (500 MHz, CD_2Cl_2, 300 K)

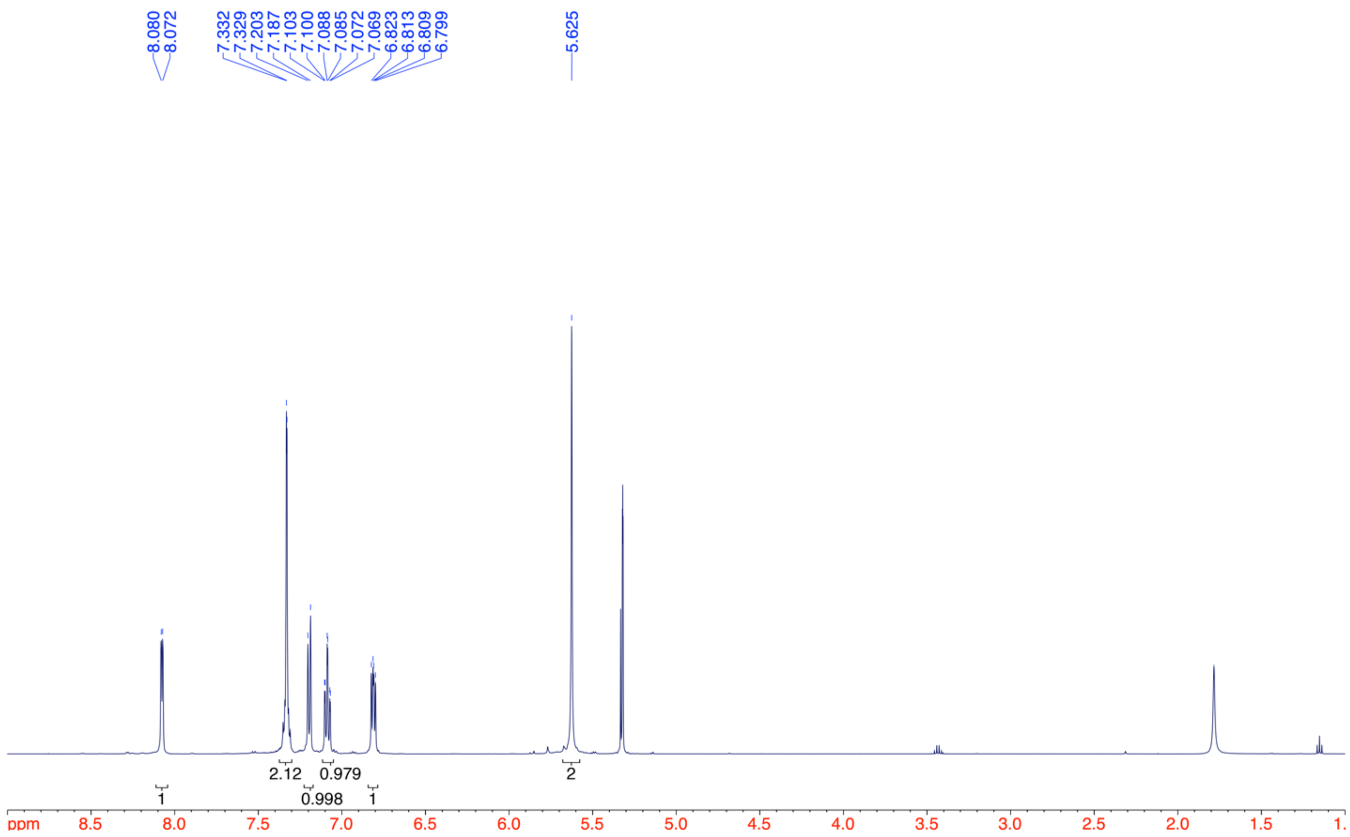

Figure S8. ^{1}H NMR spectrum of **6** (500 MHz, CD_2Cl_2, 300 K)

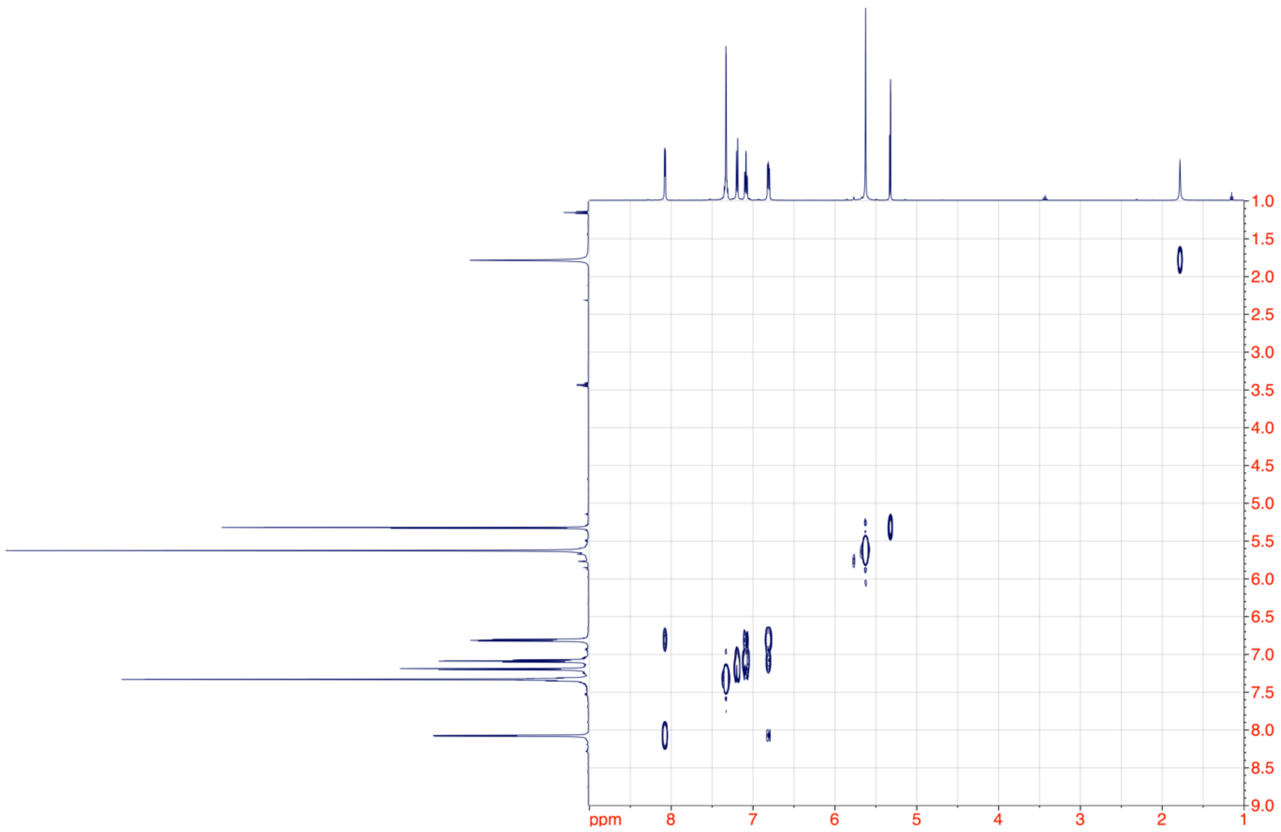

Figure S9. ^{1}H-^{1}H COSY NMR spectrum of **6** (500 MHz, CD_2Cl_2, 300 K)

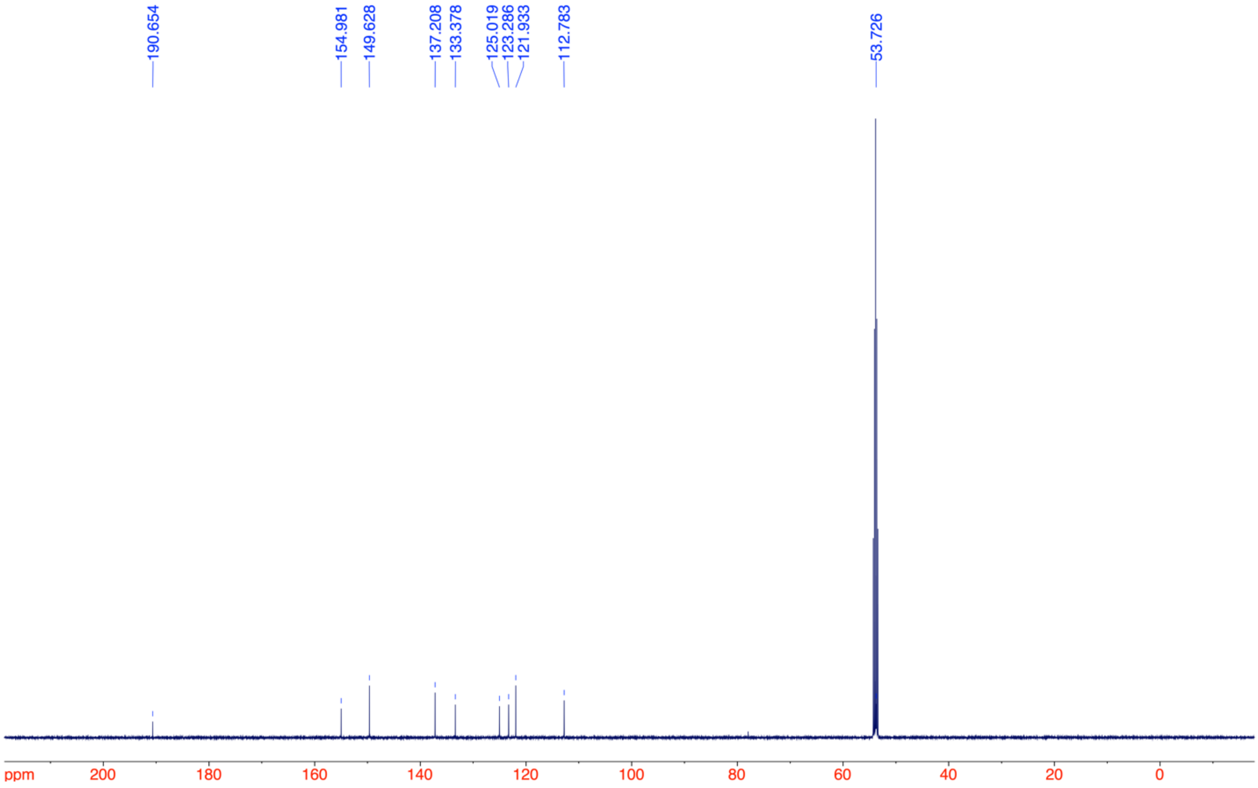

Figure S10. ^{13}C NMR spectrum of **6** (126 MHz, CD_2Cl_2, 300 K)

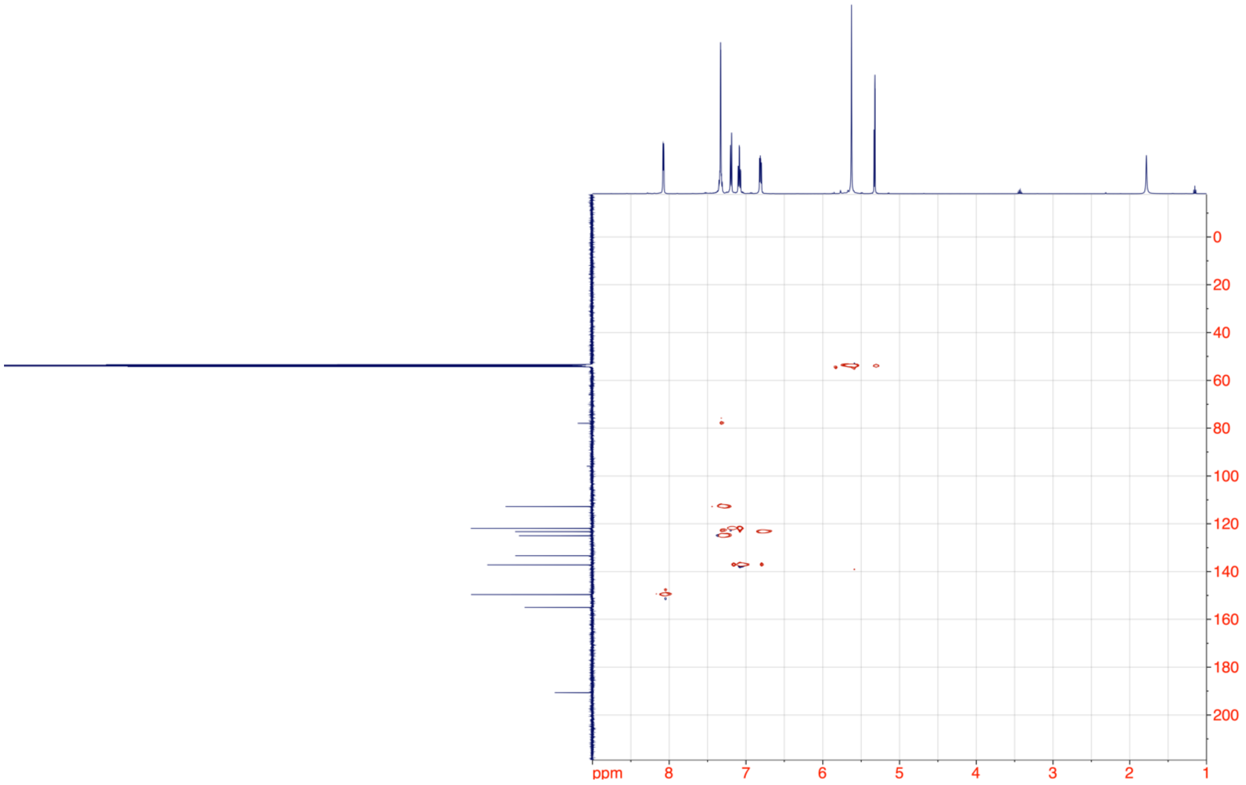

Figure S11. ^{1}H-^{13}C HSQC spectrum of **6** (500 MHz, CD_2Cl_2, 300 K)

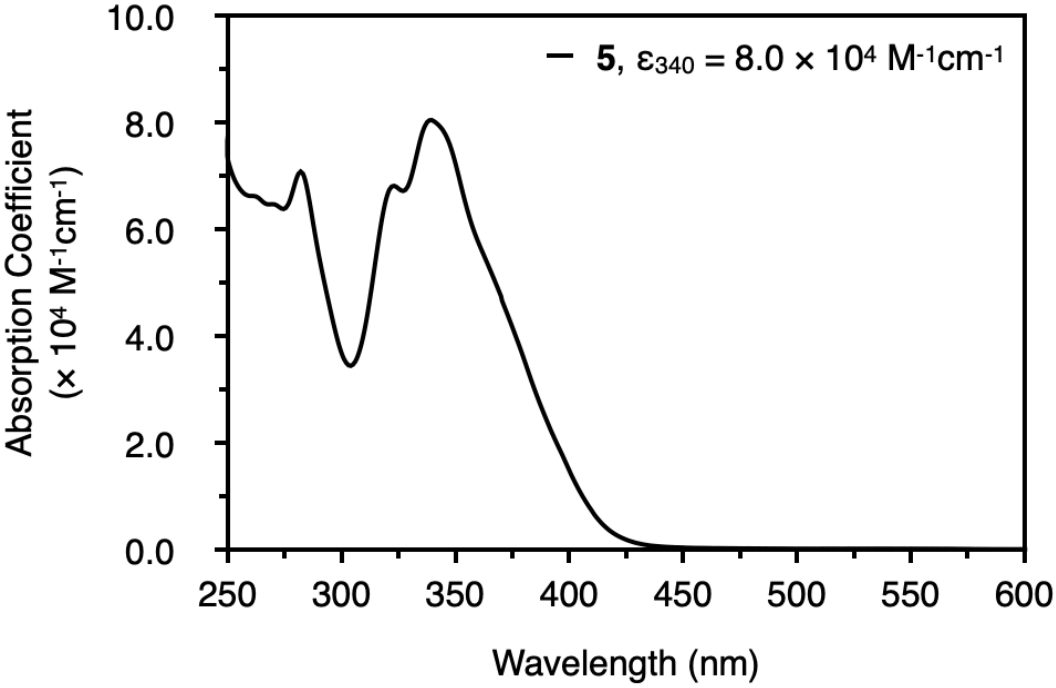

Figure S12. UV-vis absorption spectrum of **5** in CH_2Cl_2

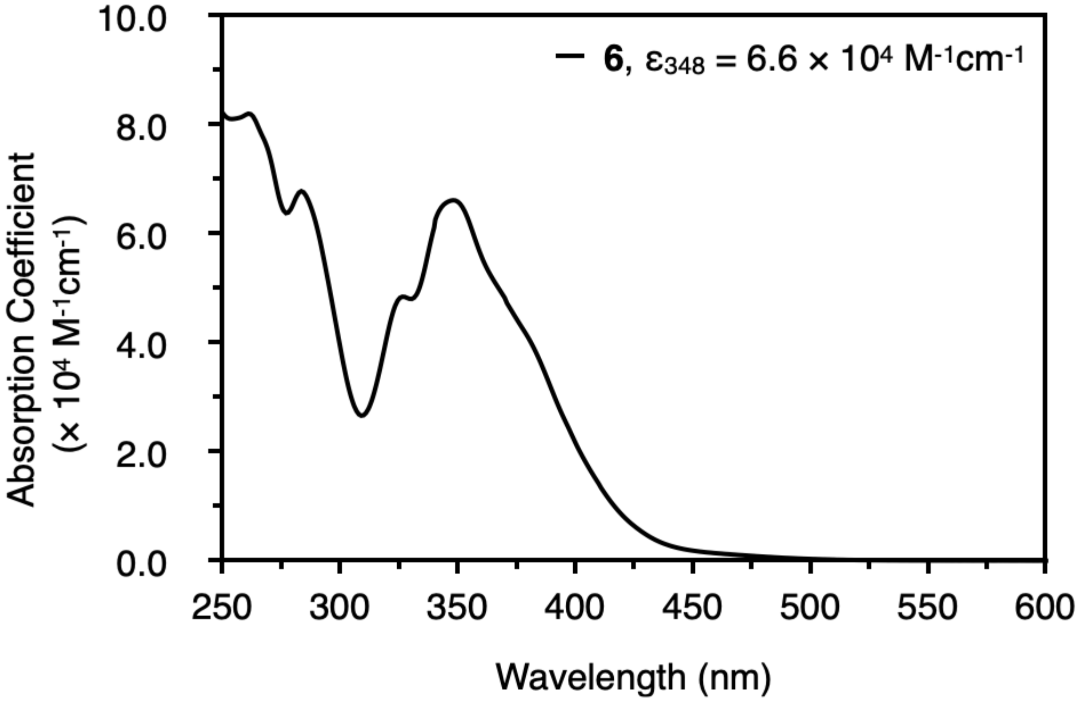

Figure S13. UV-vis absorption spectrum of **6** in CH_2Cl_2

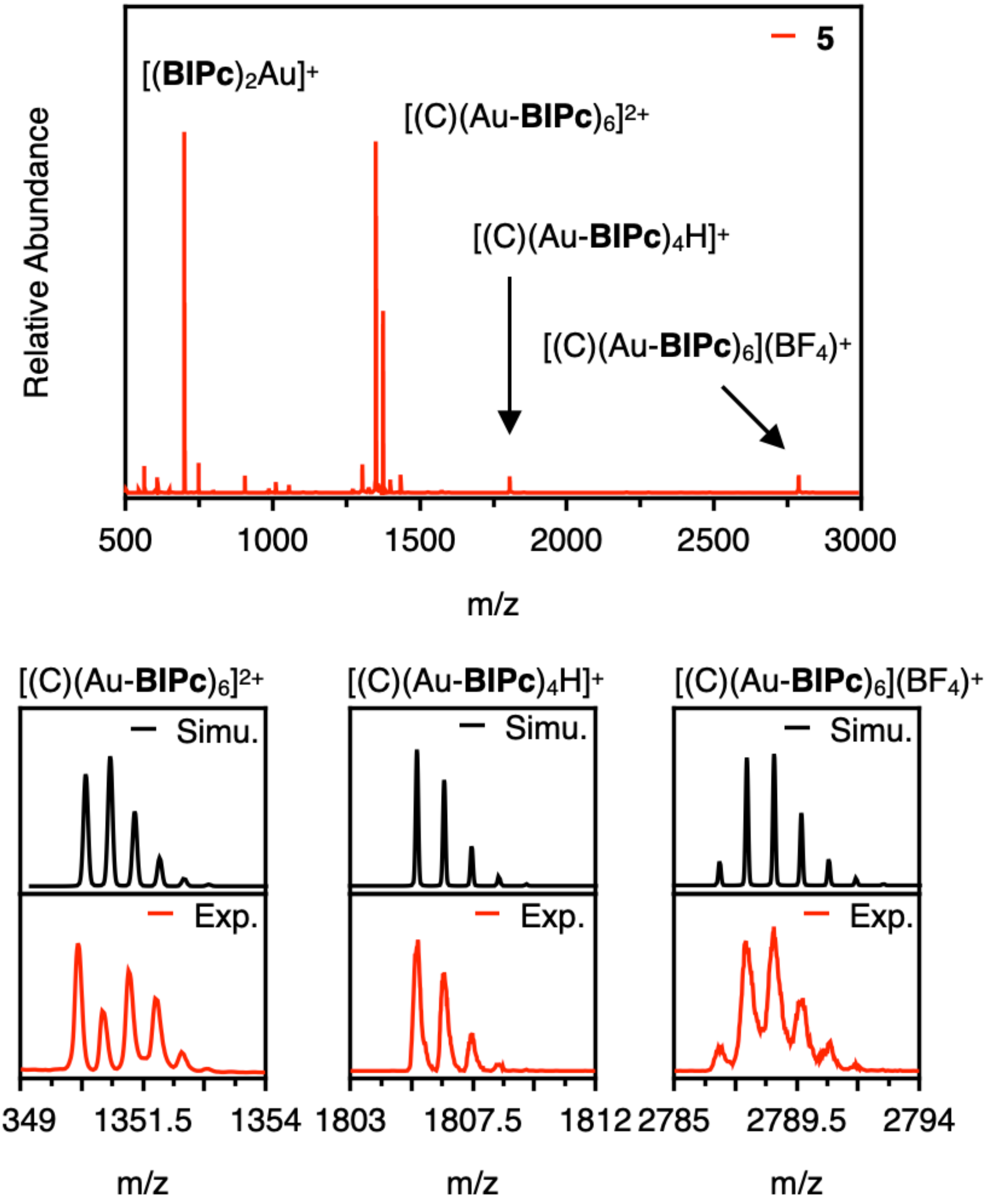

Figure S14. ESI MS spectra of **5** (CH_2Cl_2)

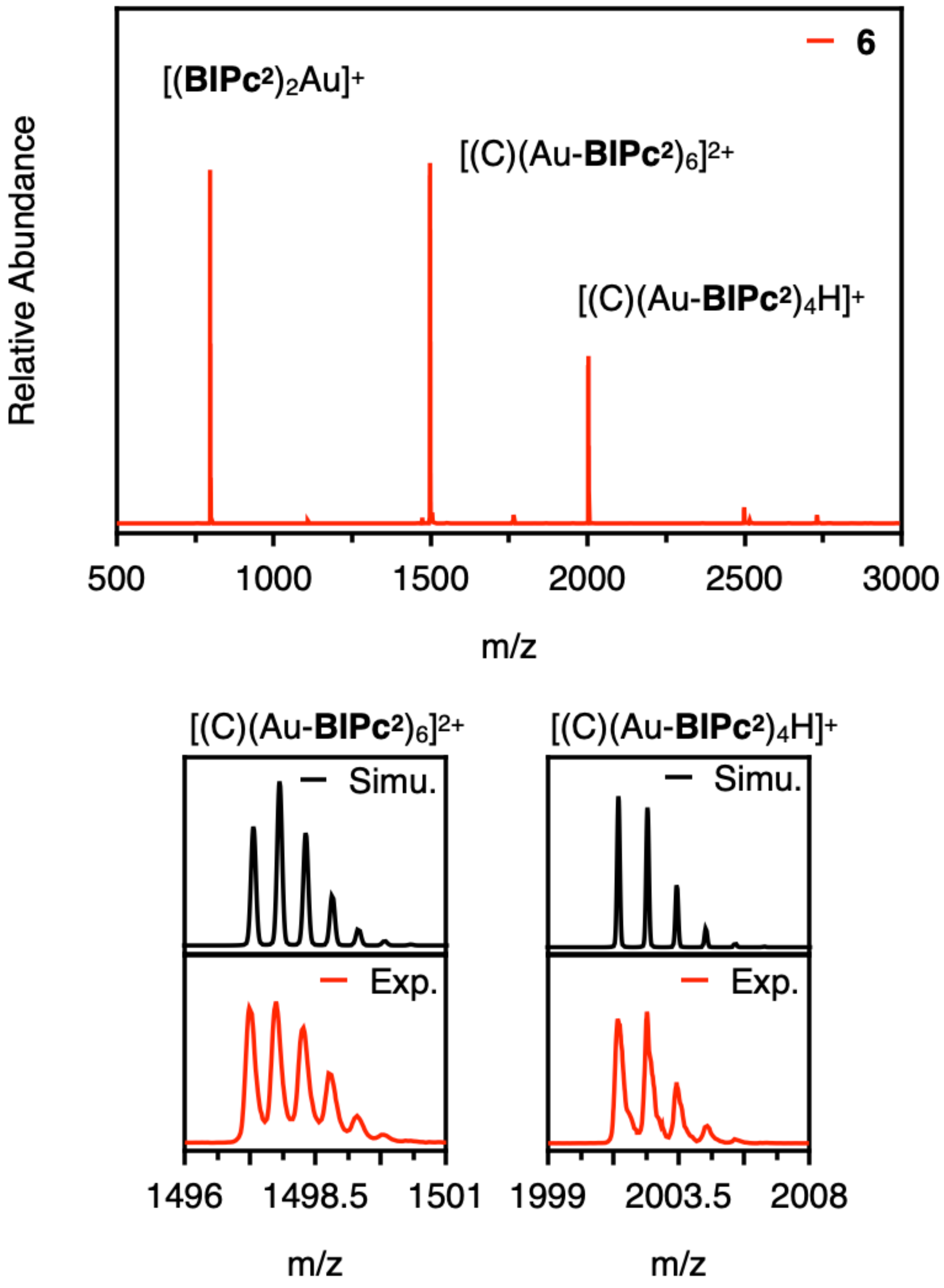

Figure S15. ESI MS spectra of **6** (CH_2Cl_2)

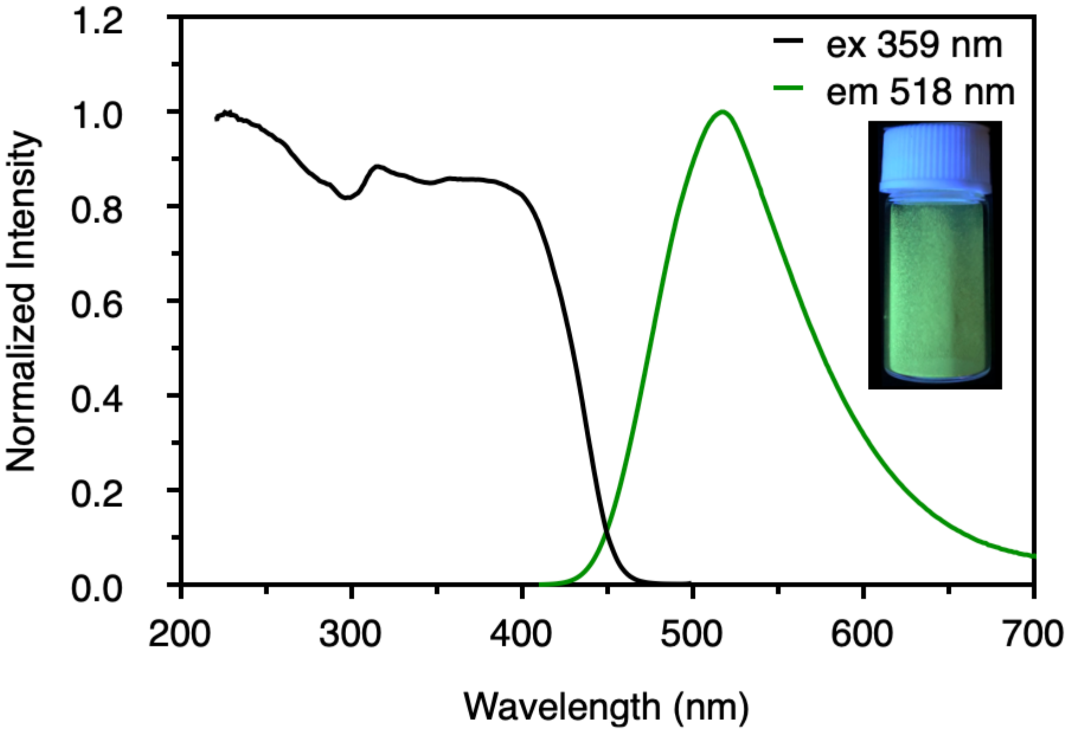

Figure S16. Excitation and emission spectra of **5** in the solid state

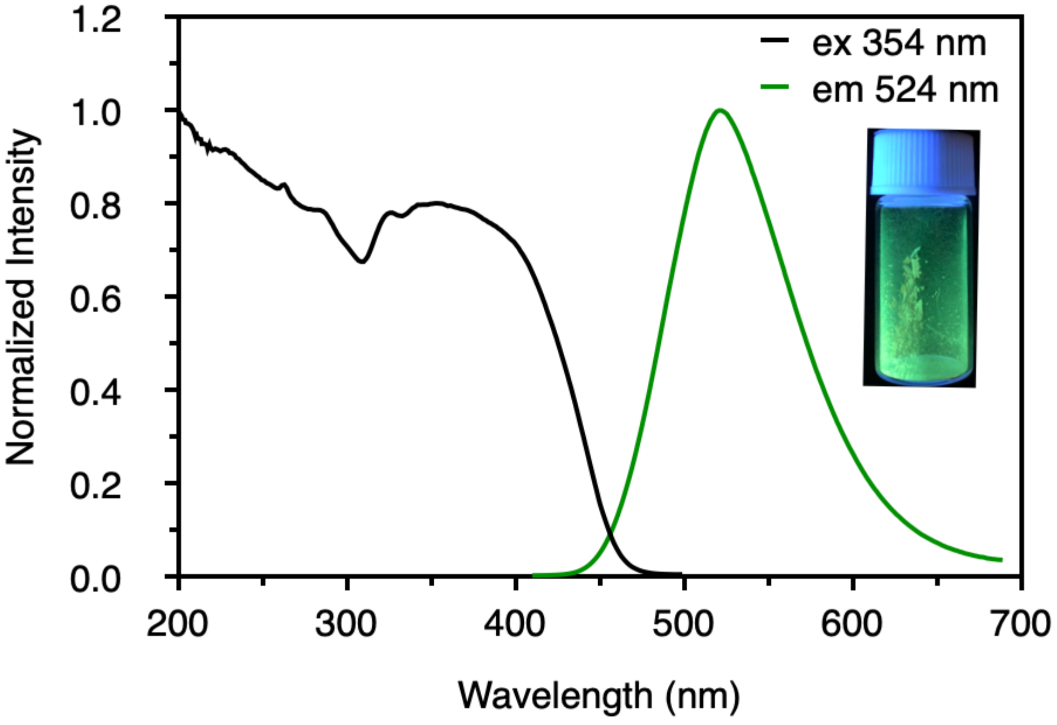

Figure S17. Excitation and emission spectra of **6** in the solid state

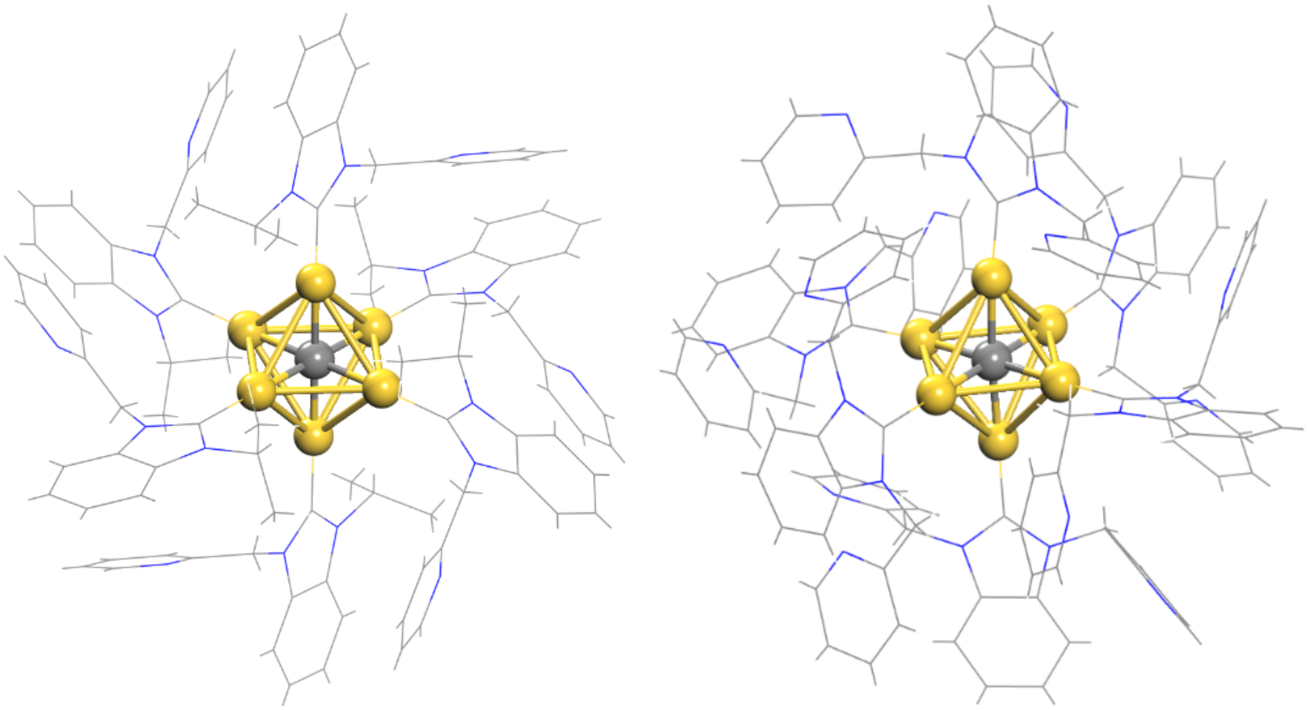

Figure S18. Optimized structures of **5** (left) and **6** (right)

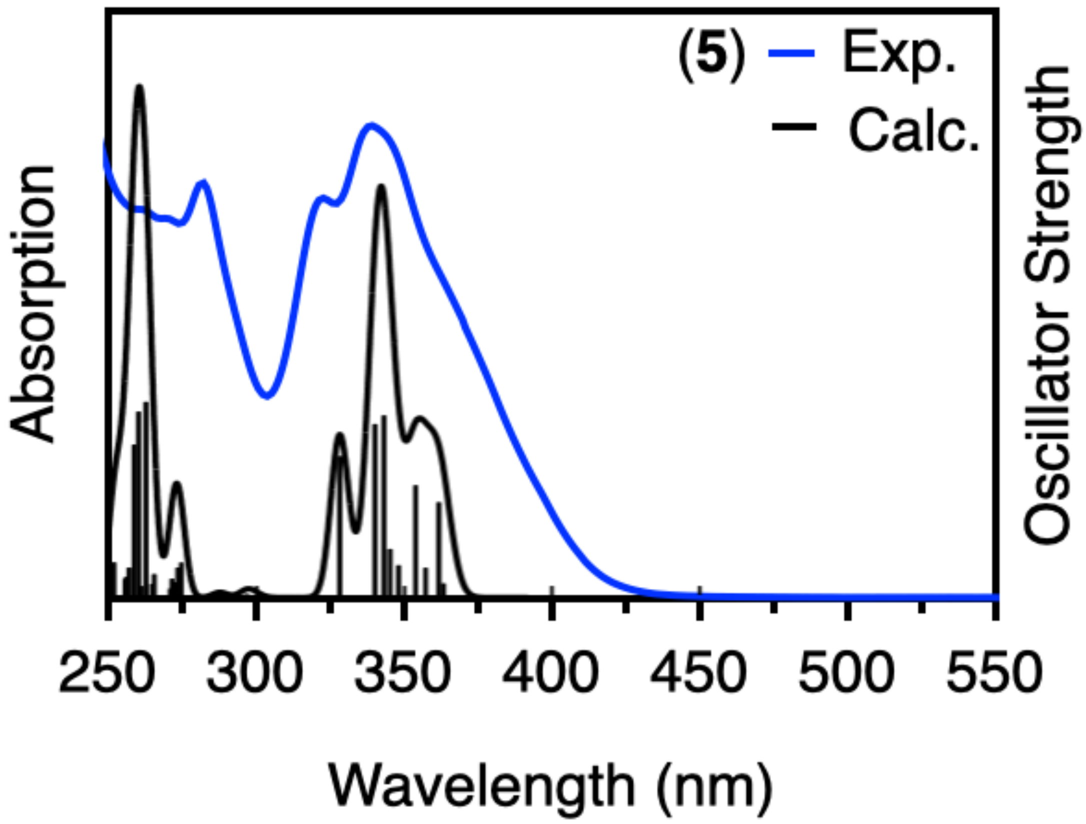

Figure S19. Simulated and experimental UV-vis absorption spectra of **5** in CH_2Cl_2

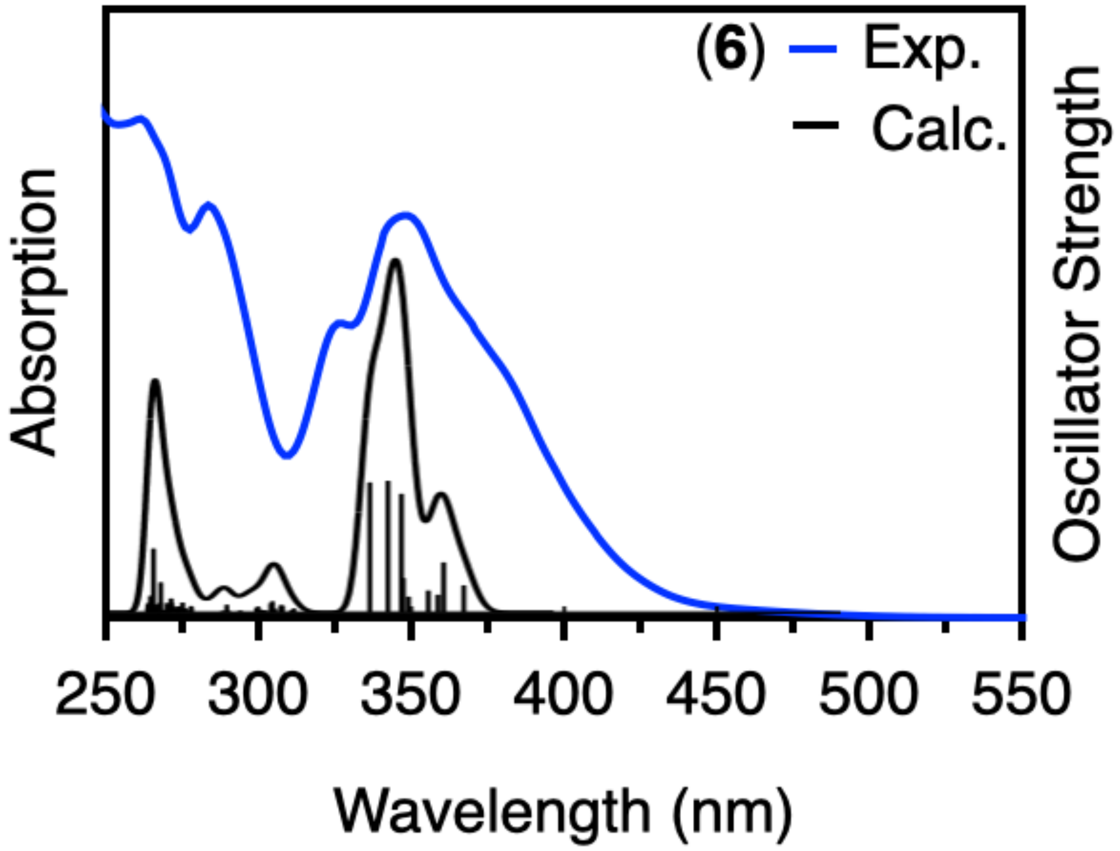

Figure S20. Simulated and experimental UV-vis absorption spectra of **6** in CH_2Cl_2

Table S2. Excited states of **5** with oscillator strength (*f*) larger than 0.02

State number	λ (nm)	ΔE (eV)	*f*	Transition character
1	363	3.413	0.0260	H–1 → L
2	362	3.428	0.1849	H → L
3	357	3.471	0.0565	H → L+1, H–1 → L+1
4	354	3.504	0.218	H–1 → L+1, H → L+1
5	348	3.562	0.0616	H–2 → L
6	345	3.592	0.0933	H → L+2
7	343	3.614	0.3547	H–2 → L+1
8	340	3.646	0.3376	H–1 → L+2, H–2 → L
9	328	3.778	0.2748	H–2 → L+2
41	275	4.516	0.0669	H → L+12, H → L+13
42	273	4.537	0.0569	H–1 → L+14
43	273	4.549	0.0275	H–1 → L+12, H → L+12, H → L+14
44	272	4.561	0.0264	H–1 → L+12, H–1 → L+14
46	271	4.569	0.0352	H → L+13
57	265	4.673	0.0442	H → L+14, H → L+18
58	265	4.686	0.0249	H–2 → L+14
61	264	4.705	0.0431	H–2 → L+13

62	263	4.723	0.3811	H–8 → L
63	262	4.736	0.0510	H–1 → L+19, H → L+19
65	261	4.750	0.0206	H–1 → L+19, H → L+19
68	260	4.768	0.3624	H–8 → L+1
71	259	4.796	0.2974	H–15 → L, H–15 → L
78	257	4.826	0.0294	H → L+12
79	257	4.828	0.0569	H–13 → L
81	256	4.839	0.039	H–15 → L
82	256	4.849	0.0375	H–1 → L+20
83	255	4.854	0.0338	H–12 → L
87	254	4.876	0.0376	H–16 → L
88	254	4.887	0.0319	H–4 → L+4
89	253	4.895	0.0320	H–12 → L+1
93	253	4.910	0.0213	H–9 → L+1
97	252	4.962	0.0671	H–16 → L+1
99	251	4.941	0.0277	H–8 → L+2
100	249	4.972	0.0285	H–2 → L+20

Table S3. Excited states of **6** with oscillator strength (*f*) larger than 0.02

State number	λ (nm)	ΔE (eV)	*f*	Transition character
1	367	3.377	0.0750	H → L
2	361	3.439	0.1403	H–2 → L, H–1 → L
3	359	3.456	0.0487	H–2 → L, H → L+1
4	356	3.487	0.0603	H–2 → L, H → L+1
5	349	0.552	0.0429	H–1 → L+1, H–1 → L+2
6	347	3.570	0.0957	H–1 → L+2
7	347	3.569	0.3346	H–1 → L+1, H → L+2
8	342	3.622	0.3714	H–2 → L+1
9	336	3.686	0.3670	H–2 → L+2
15	305	4.072	0.0298	H–1 → L+8
16	304	4.078	0.0242	H –2→ L+3, H –2→ L+7
63	275	4.506	0.0260	H–1 → L+18
71	272	4.564	0.0250	H–5 → L, H–2 → L+18
72	271	4.568	0.0381	H–2 → L+18
74	271	4.581	0.0203	H–7 → L, H–2 → L+19
77	270	4.590	0.0243	H–1 → L+23
82	268	4.626	0.0833	H–8 → L, H–2 →L+22
83	268	4.628	0.0261	H–2 → L+22, H–2 → L+23
85	268	4.634	0.0233	H → L+27
91	266	4.663	0.0213	H–1 → L+26
92	266	4.668	0.1793	H–10 → L
94	265	4.680	0.0291	H–2 → L+24, H–1→ L+28
96	265	4.685	0.0450	H–6 → L+1
98	264	4.699	0.0239	H–9 → L

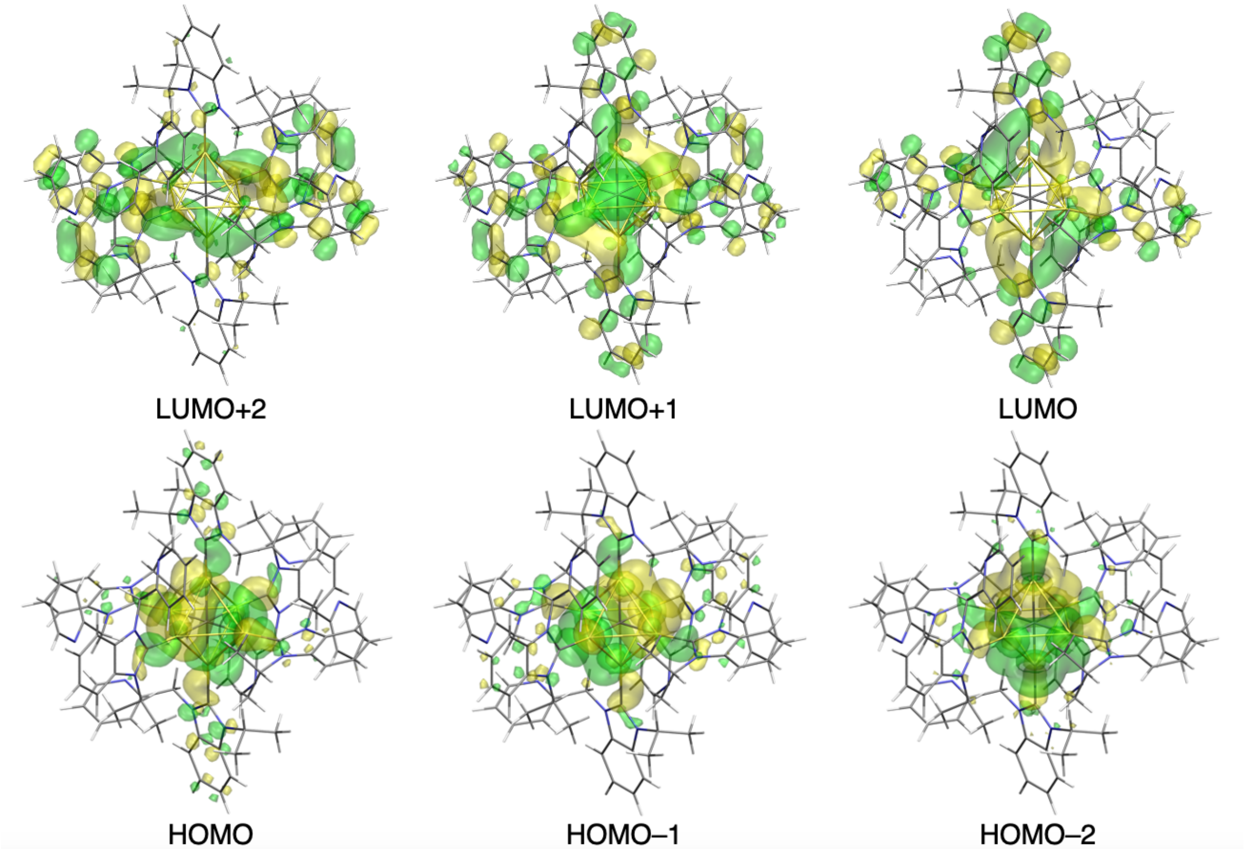

Figure S21. Involved molecular orbitals of **5**

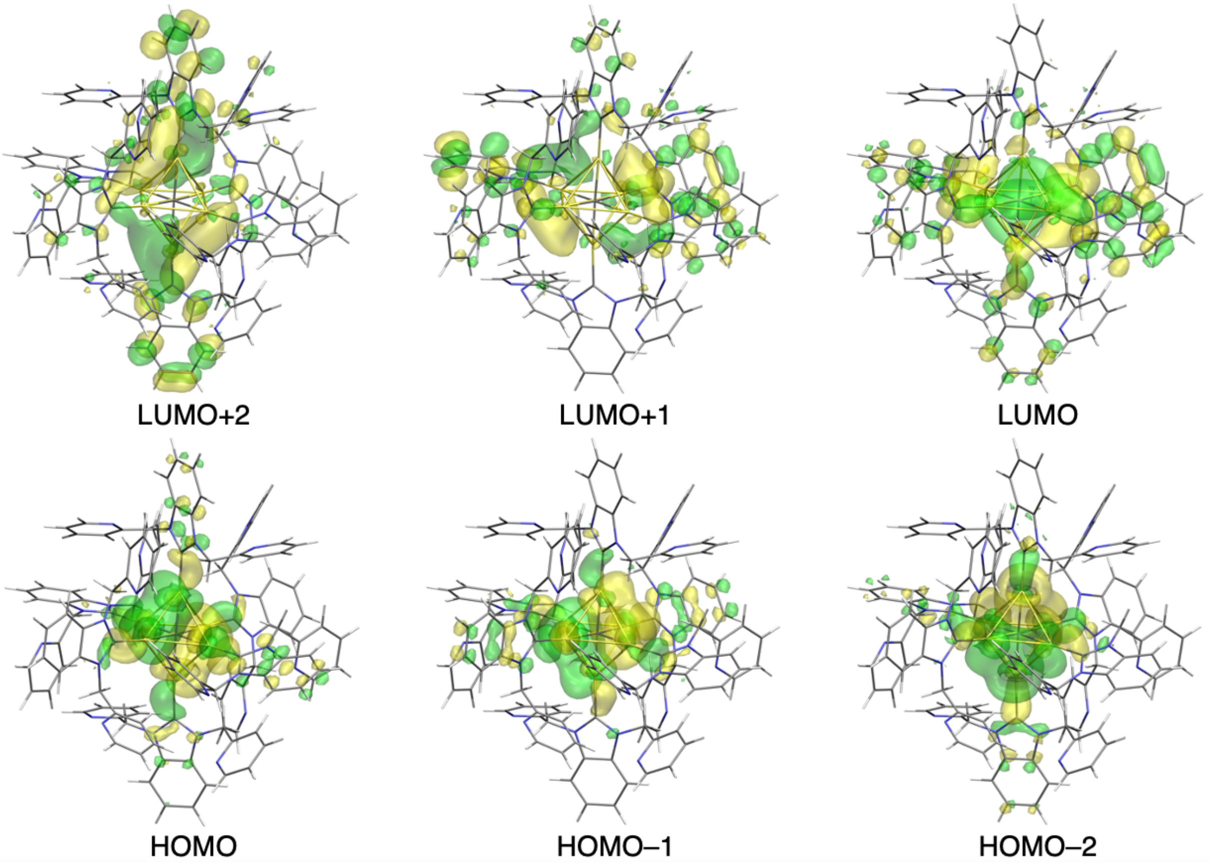

Figure S22. Involved molecular orbitals of **6**

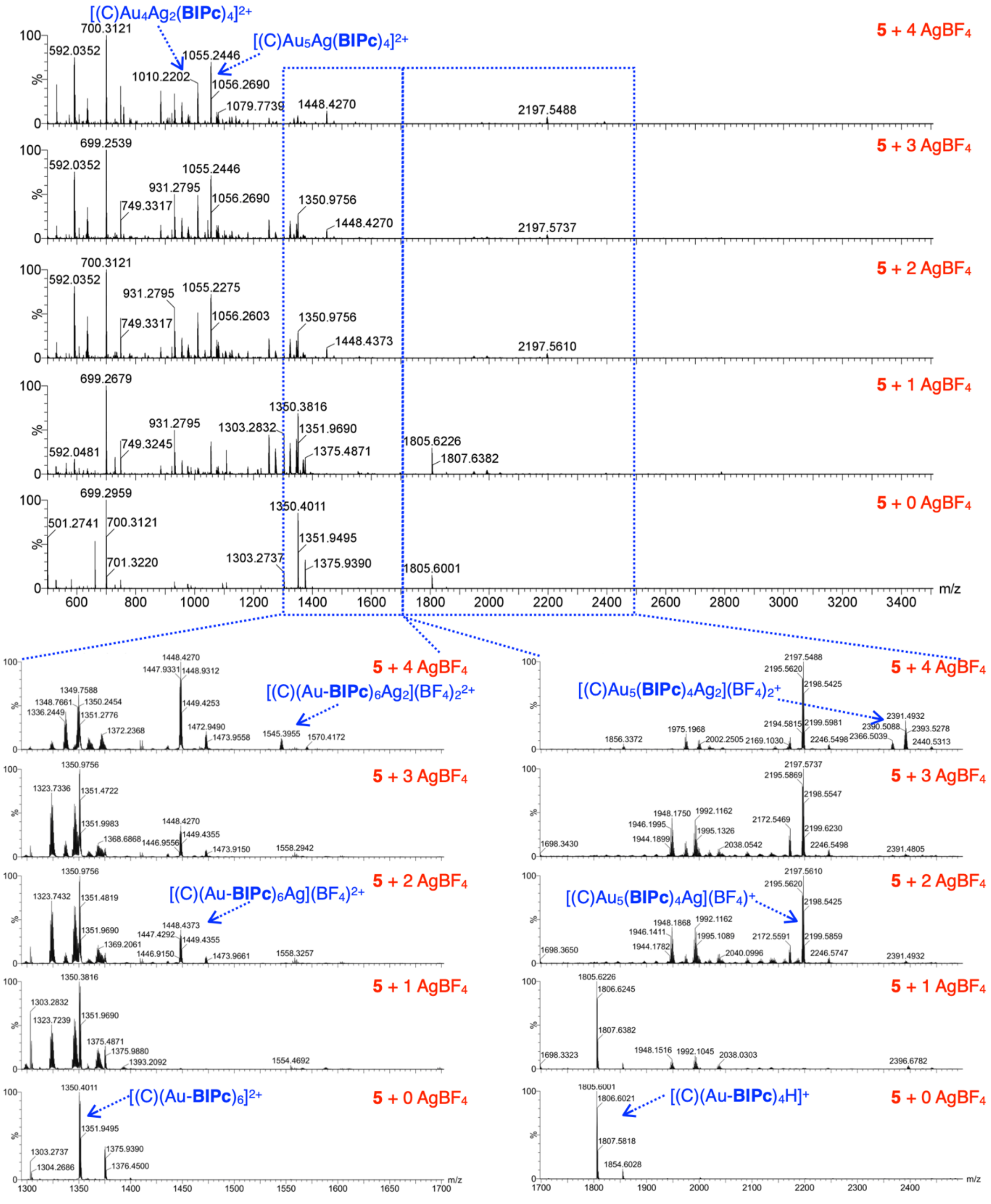

Figure S23. MS spectra for complexation of **5** with $AgBF_4$ in CH_2Cl_2/CH_3CN (9:1, v:v)

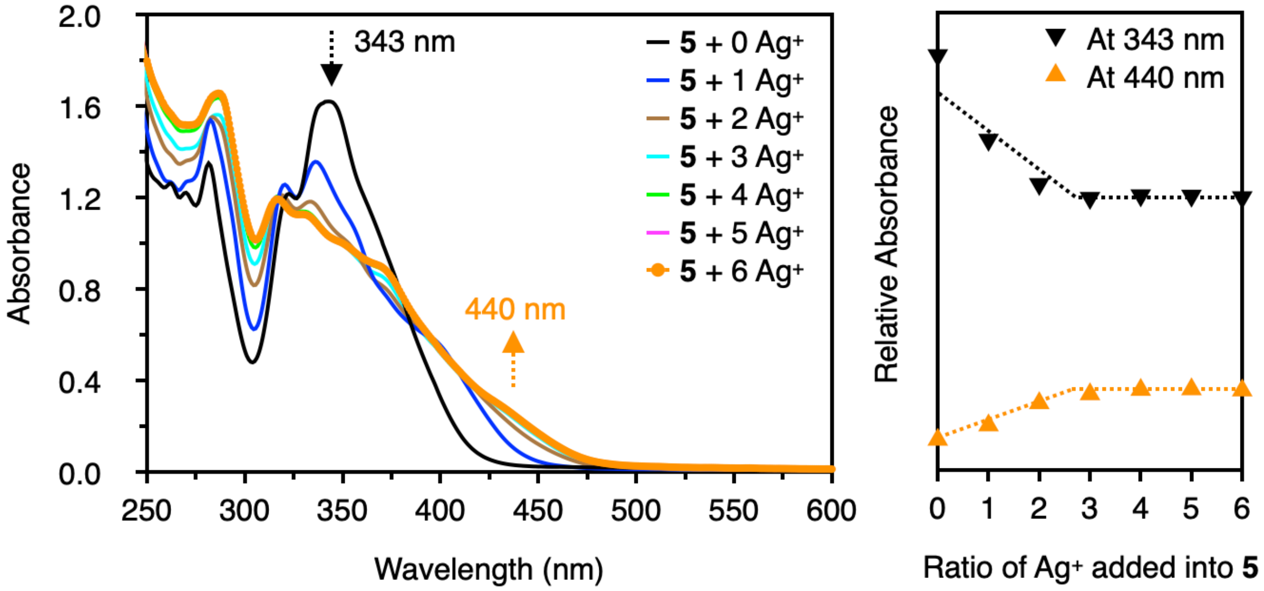

Figure S24. UV-vis absorption spectra for complexation of **5** with $AgBF_4$ in CH_2Cl_2/CH_3CN (9:1, v:v)

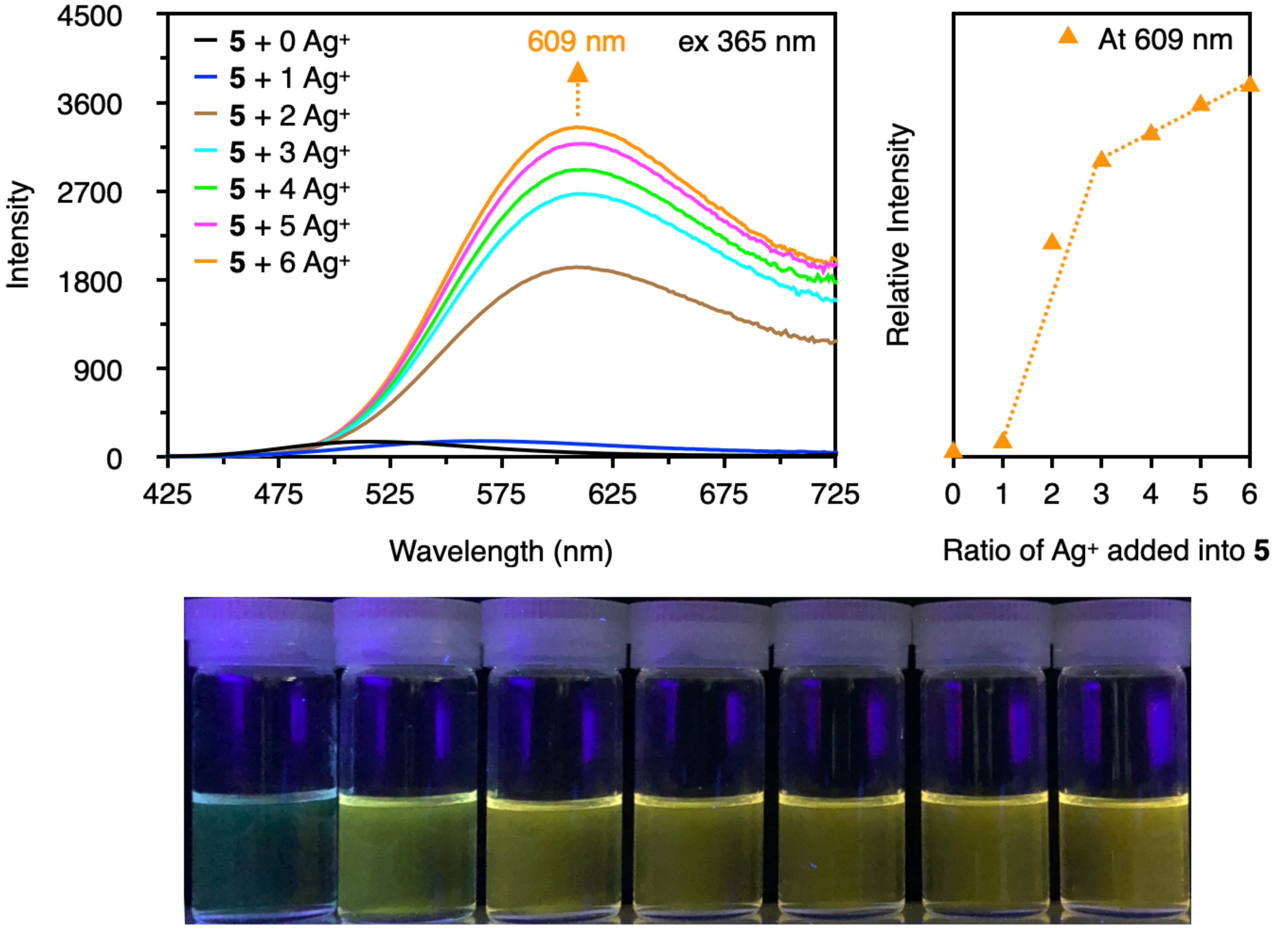

Figure S25. Emission spectra for complexation of **5** with $AgBF_4$ in CH_2Cl_2/CH_3CN (9:1, v:v)

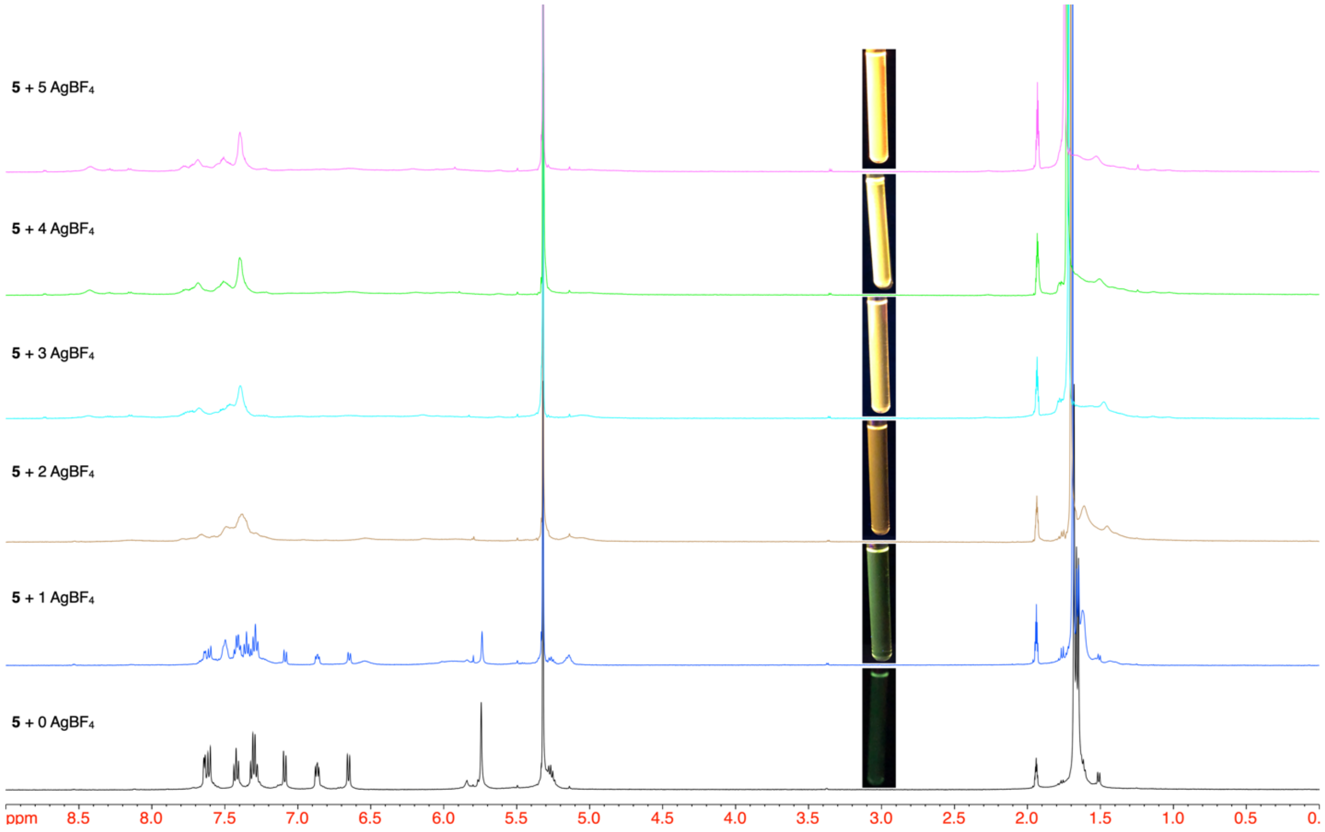

Figure S26. ^{1}H NMR spectra for complexation of **5** with $AgBF_4$ in CD_2Cl_2/CD_3CN (9:1, v:v)

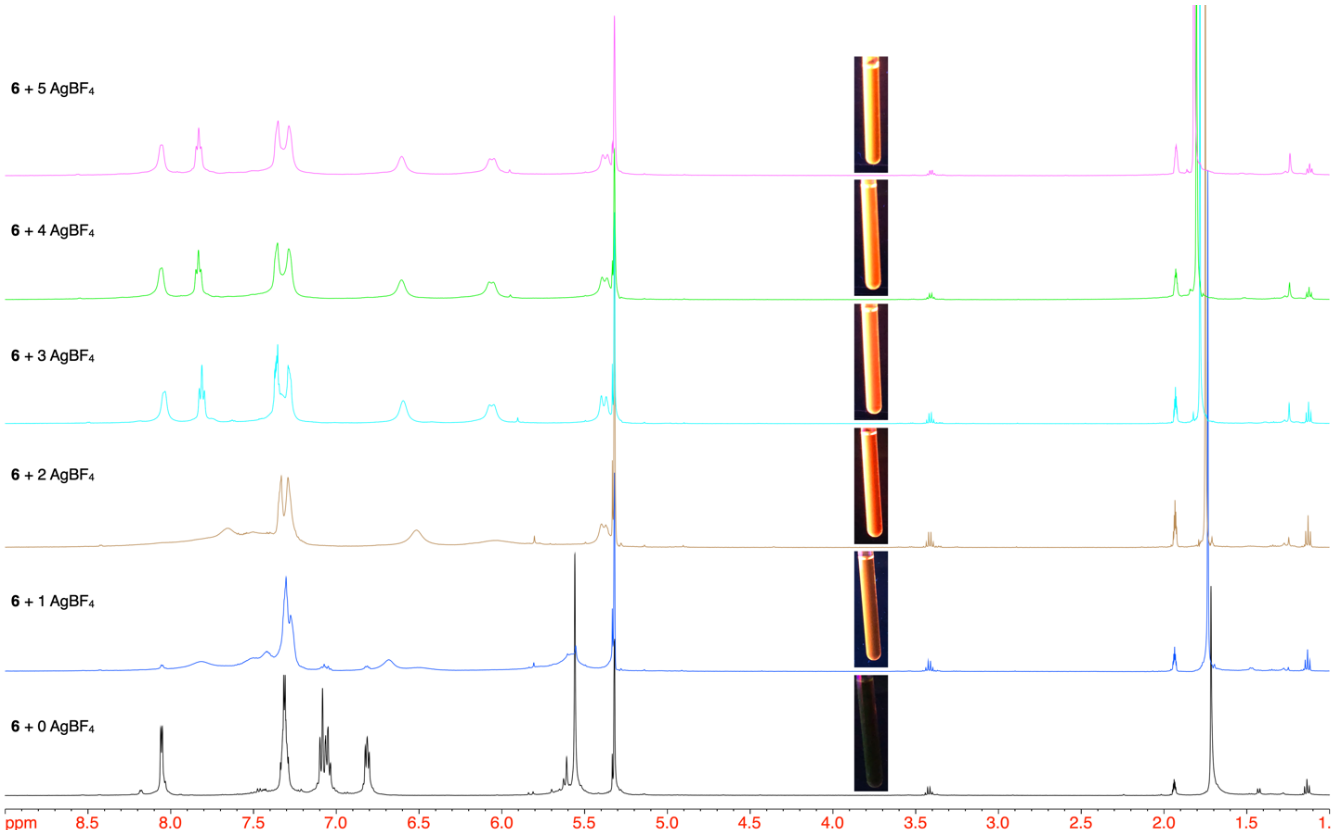

Figure S27. ^{1}H NMR spectra for complexation of **6** with $AgBF_4$ in CD_2Cl_2/CD_3CN (9:1, v:v)

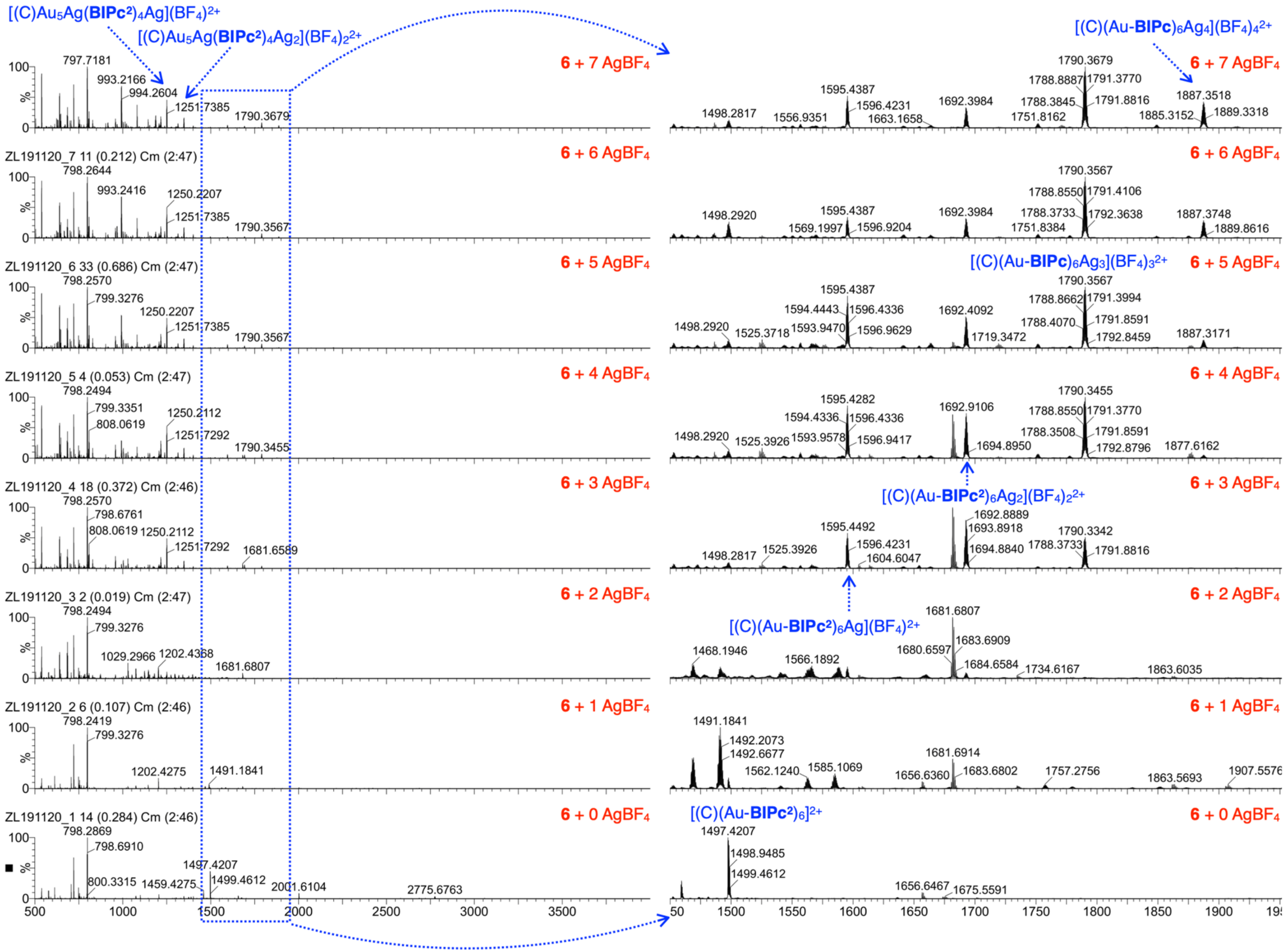

Figure S28. MS spectra for complexation of **6** with $AgBF_4$ in CH_2Cl_2/CH_3CN (9:1, v:v)

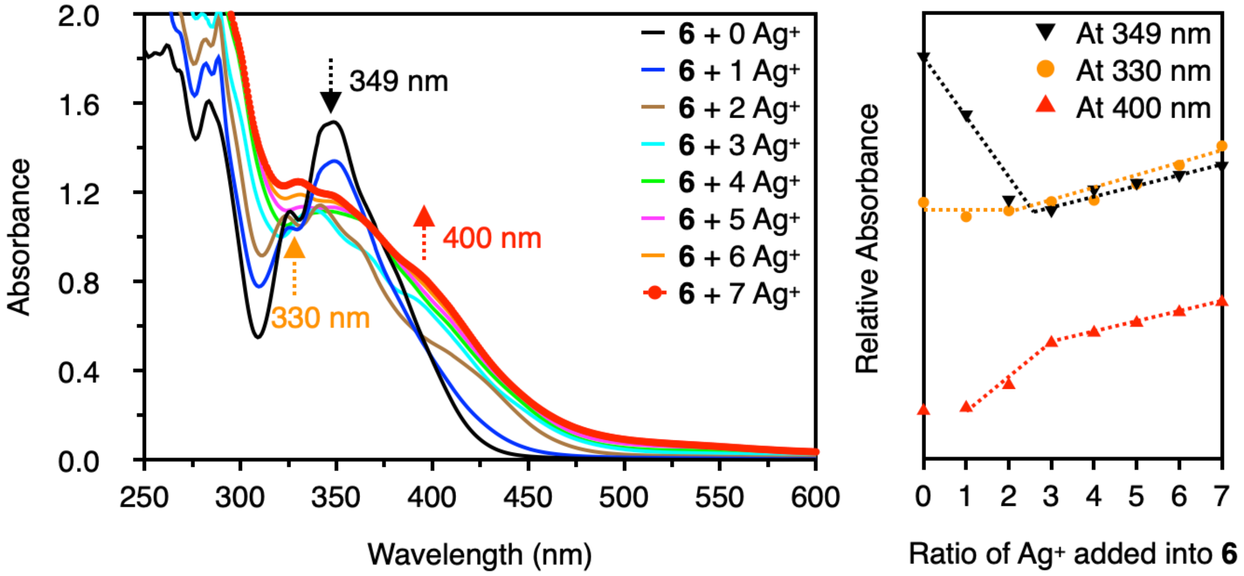

Figure S29. UV-vis absorption spectra for complexation of **6** with $AgBF_4$ in CH_2Cl_2/CH_3CN (9:1, v:v)

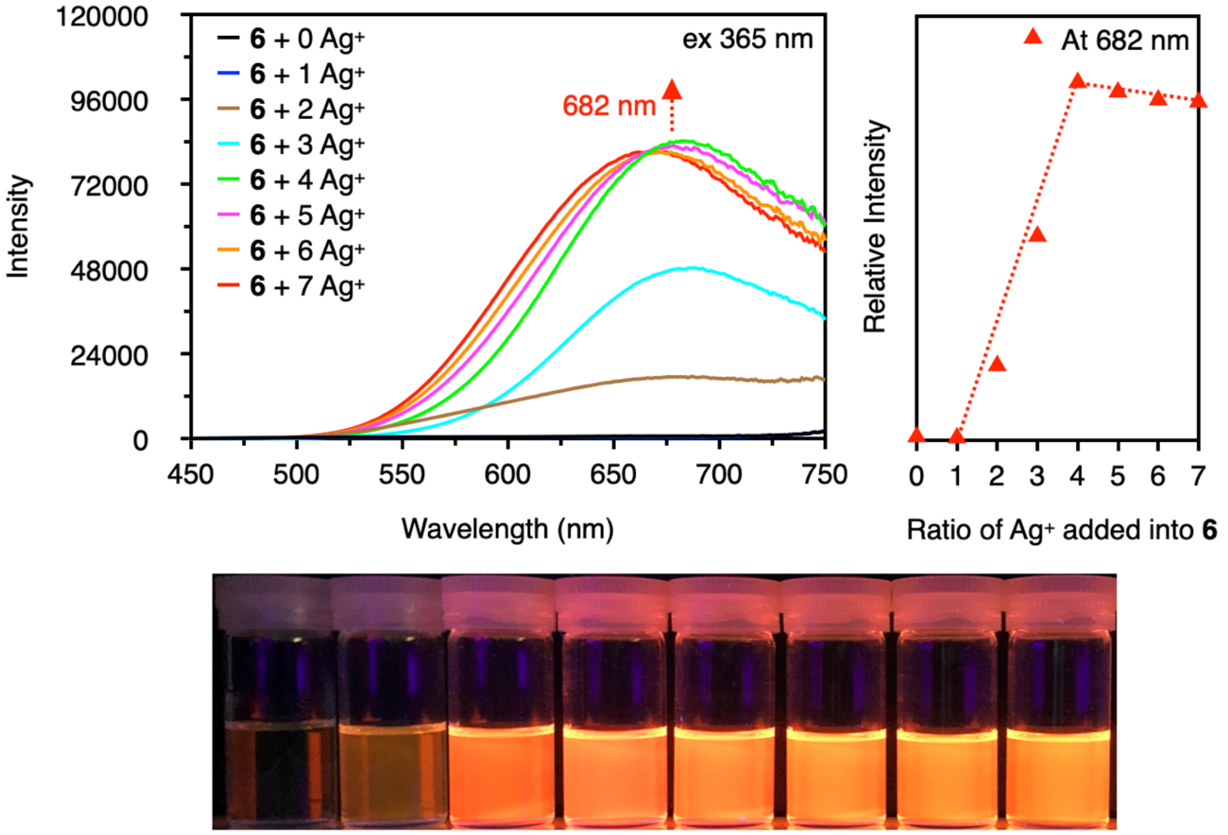

Figure S30. Emission spectra for complexation of **6** with $AgBF_4$ in CH_2Cl_2/CH_3CN (9:1, v:v)

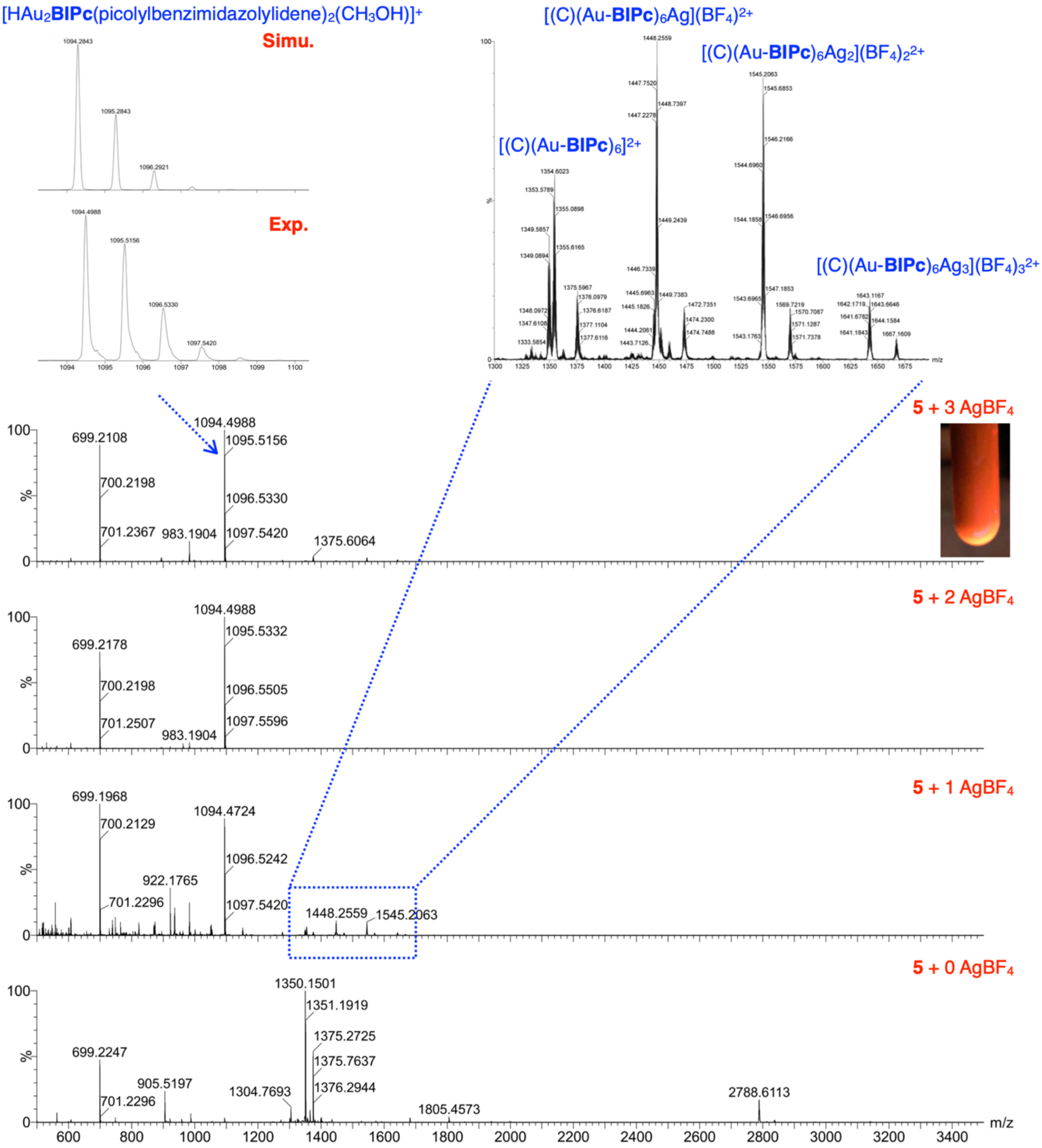

Figure S31. MS spectra for complexation of **5** with $AgBF_4$ in CH_2Cl_2/CH_3OH (9:1, v:v)

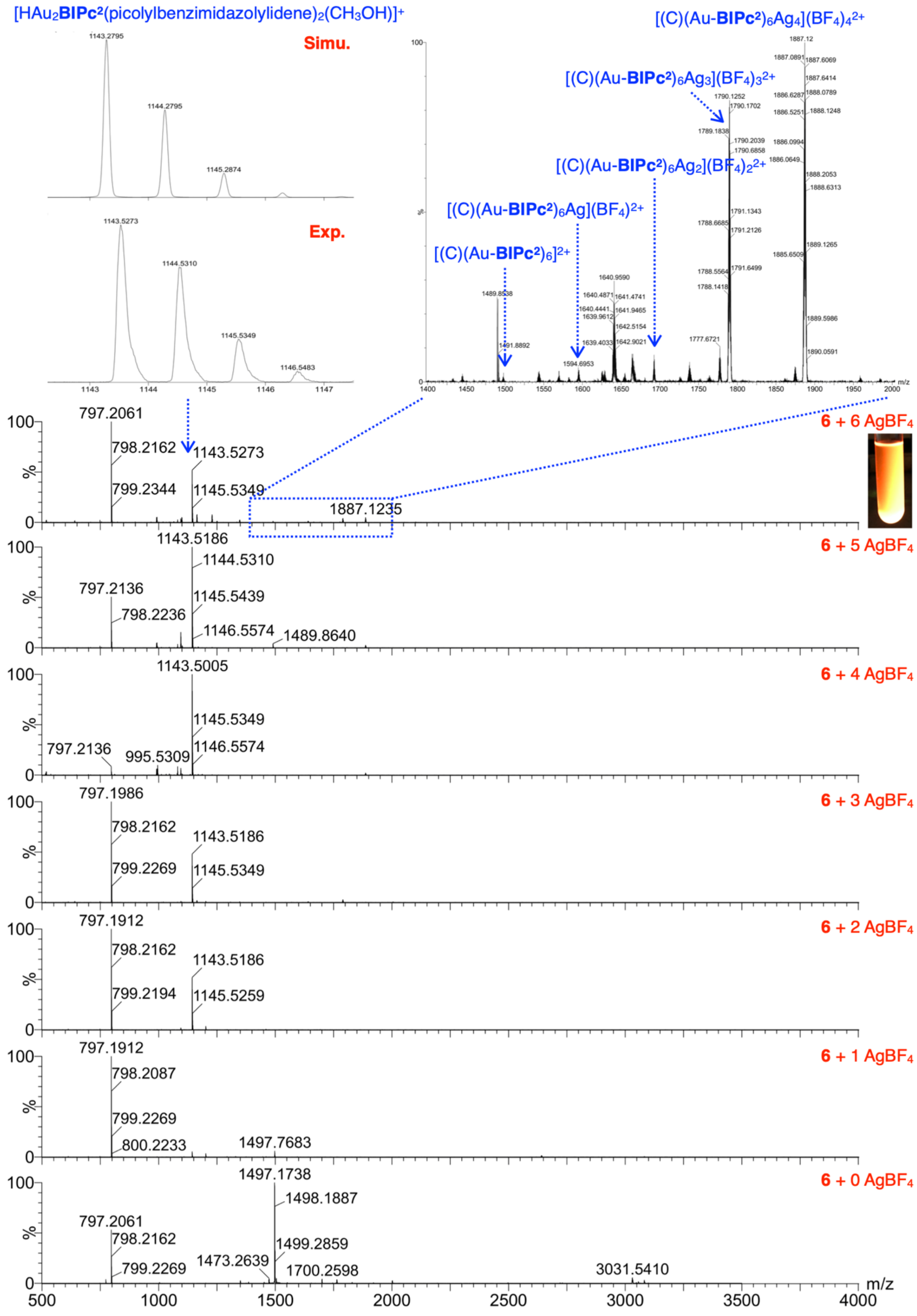

Figure S32. MS spectra for complexation of **6** with $AgBF_4$ in CH_2Cl_2/CH_3OH (9:1, v:v)

Table S4. Key structural parameters of **2**-**4** and some reported $CAu^{I}_{6}Ag^{I}_{n}$ clusters (*Corresponds to Au–P distances for phosphine-protected clusters)

	**Au–C (central C^{4-}, Å)**	**Au–Au (Å)**	**Au–C (NHC ligands, Å)***	**Au–Ag (Å)**	**Ag–N (Å)**
[(C)(Au-**BIPy**)$_6$Ag$_2$](BF$_4$)$_4$ (**2**)[1]	2.1100(6)	2.9474(10)–3.0200(11)	2.00(2)	2.8467(17)	2.337(10)–2.34(2)
[(C)(Au-**BIMPy**)$_6$Ag$_2$](BF$_4$)$_4$[1]	2.1102(4)–2.1185(4)	2.9471(5)–3.0252(5)	2.025(10)–2.027(10)	2.8372(8)–2.8729(8)	2.393(8)–2.402(9)
**3**	2.087(13)–2.16(3)	2.9149(10)–3.1684(9)	2.01(2)–2.083(14)	2.8268(14)–2.9754(14)	2.346(8)–2.34(2)
**4**	2.073(8)–2.162(7)	2.9328(5)–3.0942(4)	2.006(9)–2.043(8)	2.7425(7)–3.0507(7)	2.393(8)–2.445(12)
[(C)(Au^{I}-dppy)$_6$Ag$^{I}_2$](BF$_4$)$_4$[8]	2.0980(4)–2.1144(4)	2.9406(5)–3.0164(5)	2.250(2)–2.266 (2)	2.9134(8)–2.9316 (8)	2.232(8)–2.499(7)
[(C)(Au^{I}-PPhPy$_2$)$_6$Ag$^{I}_4$](BF$_4$)$_6$[9]	2.1129(5)–2.1183(5)	2.9370(7)–3.0521(7)	2.265(4)–2.276(3)	2.8509(11)–2.9337(11)	2.312(12)–2.415(12)
[(C)(Au^{I}-PPhPy$_2$)$_6$Ag$^{I}_6$(tfa)$_3$](BF$_4$)$_5$[9]	2.135(12)–2.159(12)	2.9136(7)–3.3291(7)	2.293(3)–2.303(3)	2.8000(12)–2.8907(11)	2.318(10)–2.408(10)

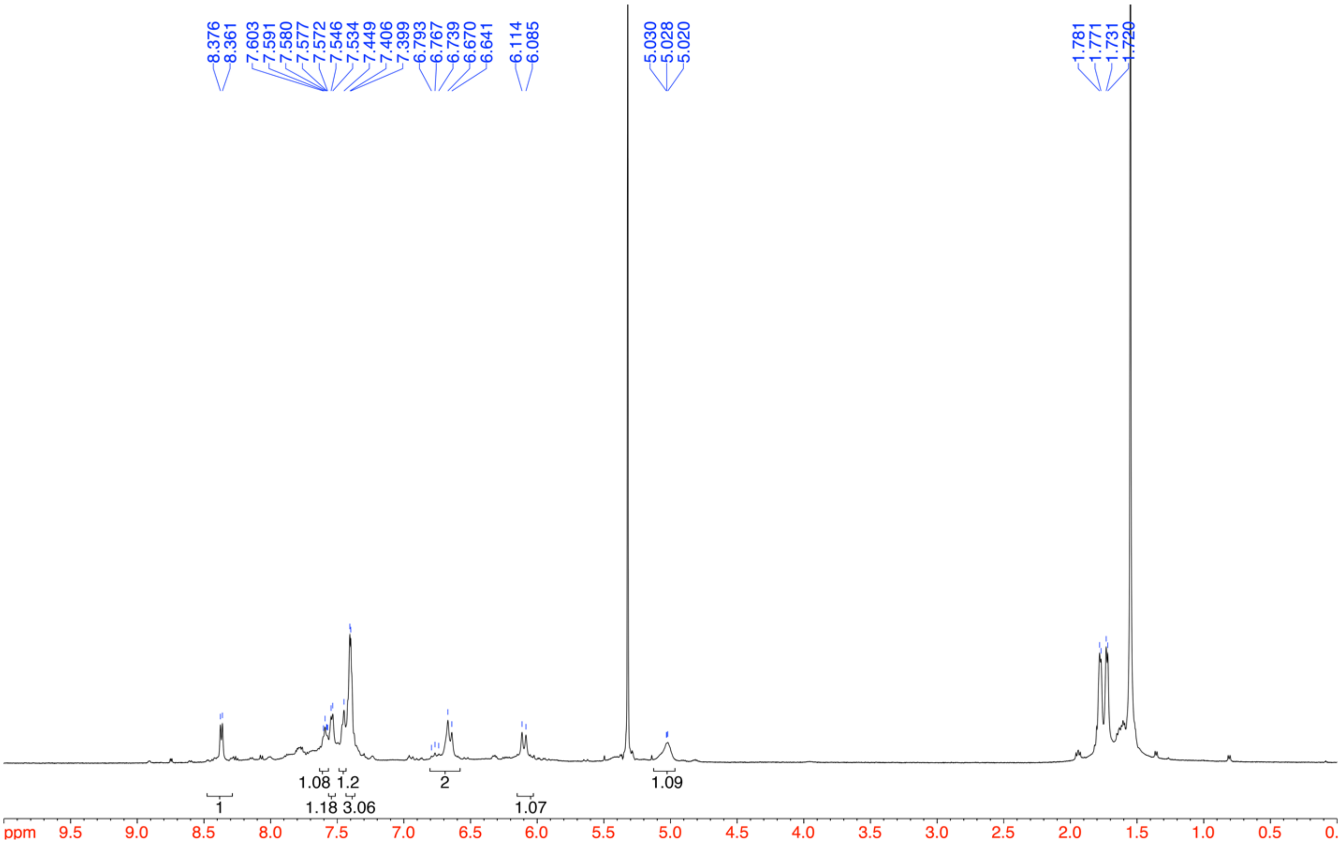

Figure S33. ^{1}H NMR spectrum of **3** (500 MHz, CD_2Cl_2, 300 K)

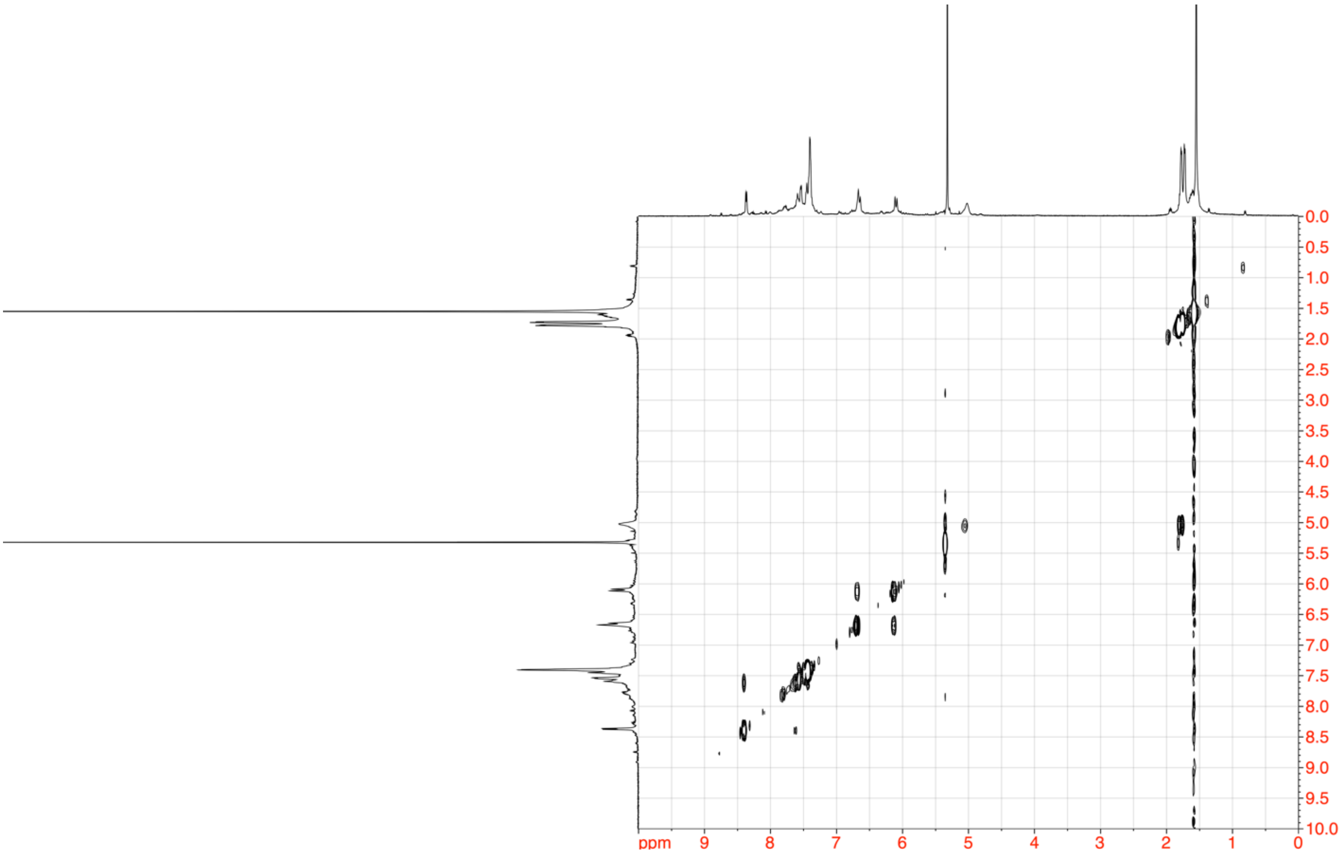

Figure S34. ^{1}H-^{1}H COSY NMR spectrum of **3** (500 MHz, CD_2Cl_2, 300 K)

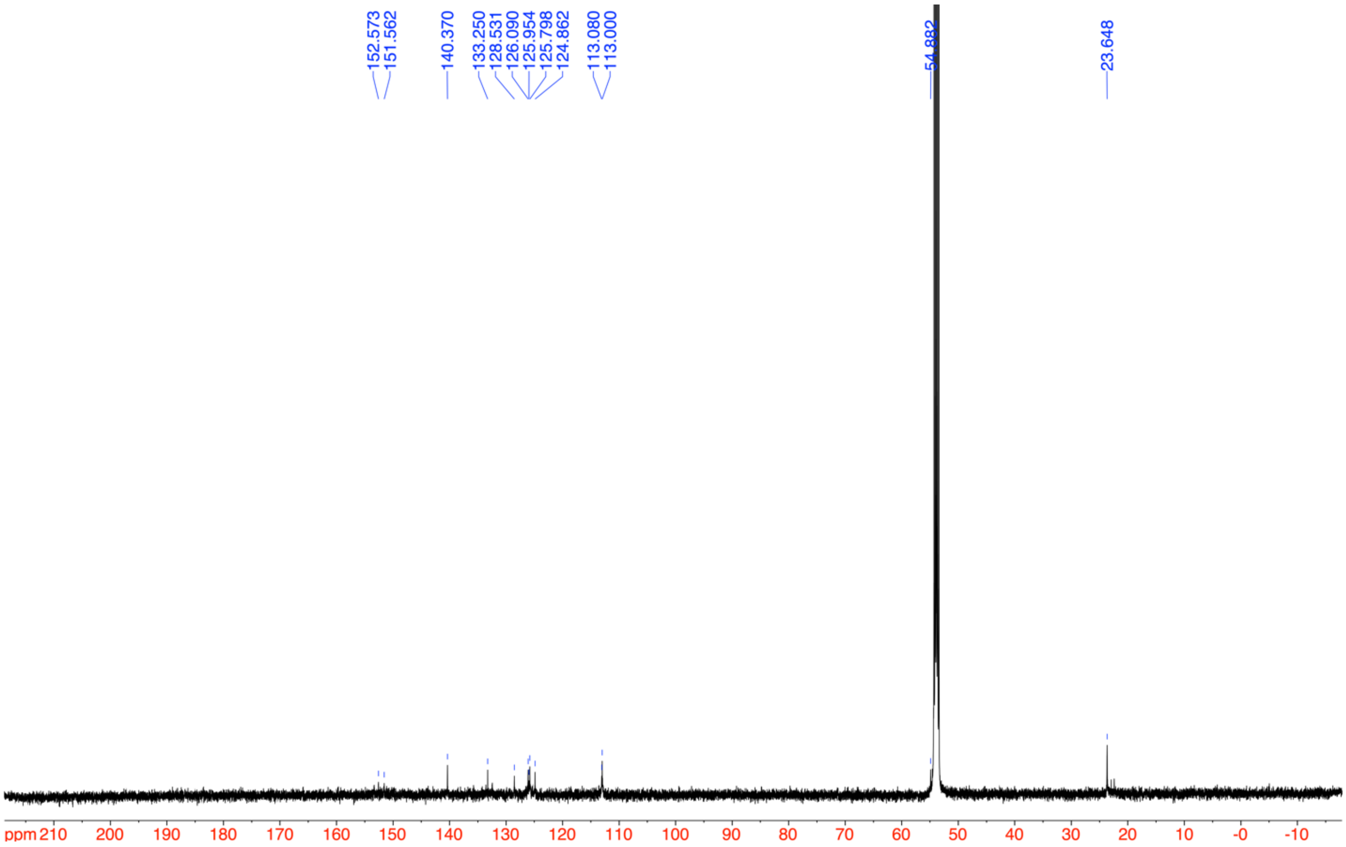

Figure S35. ^{13}C NMR spectrum of **3** (126 MHz, CD_2Cl_2, 300 K)

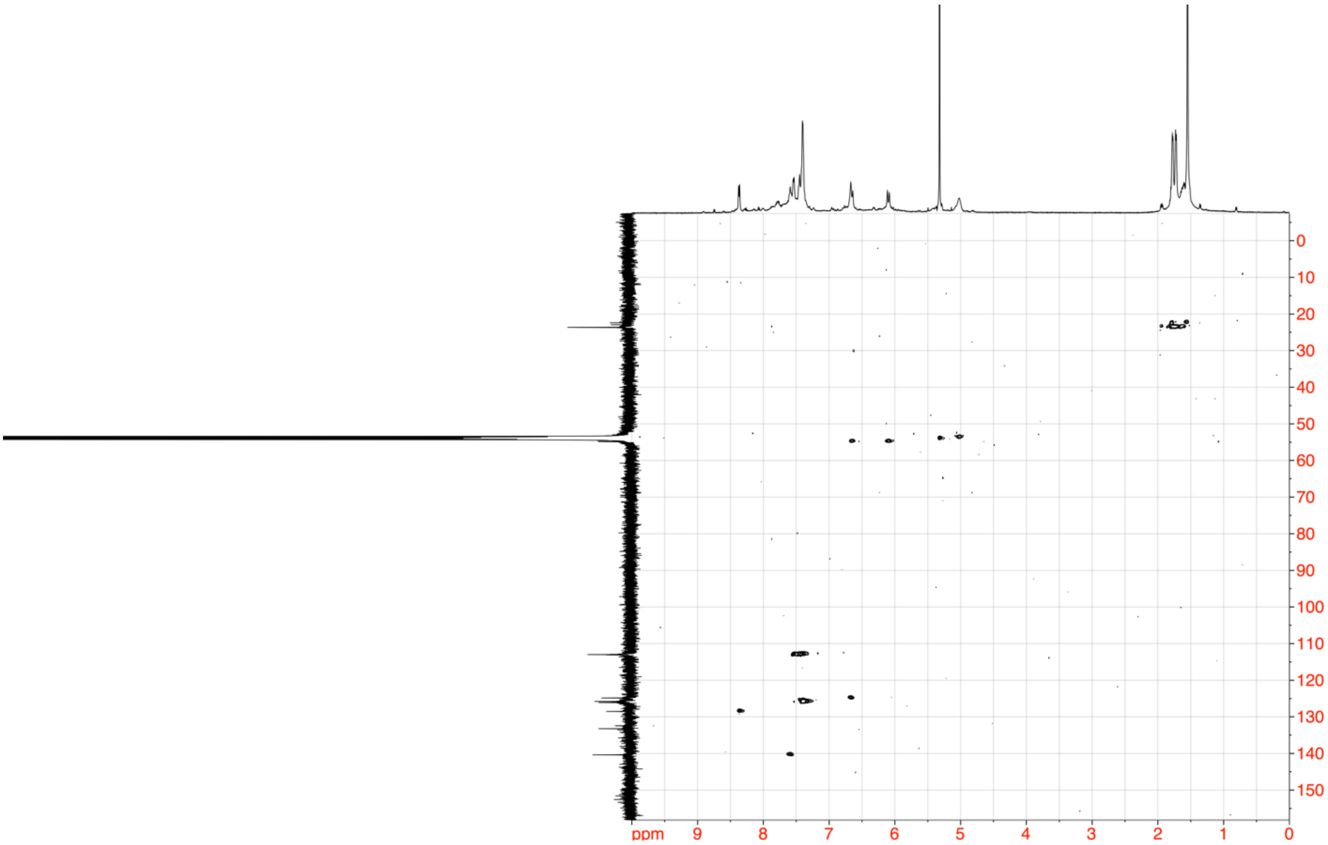

Figure S36. ^1H-^{13}C HSQC spectrum of **3** (500 MHz, CD_2Cl_2, 300 K)

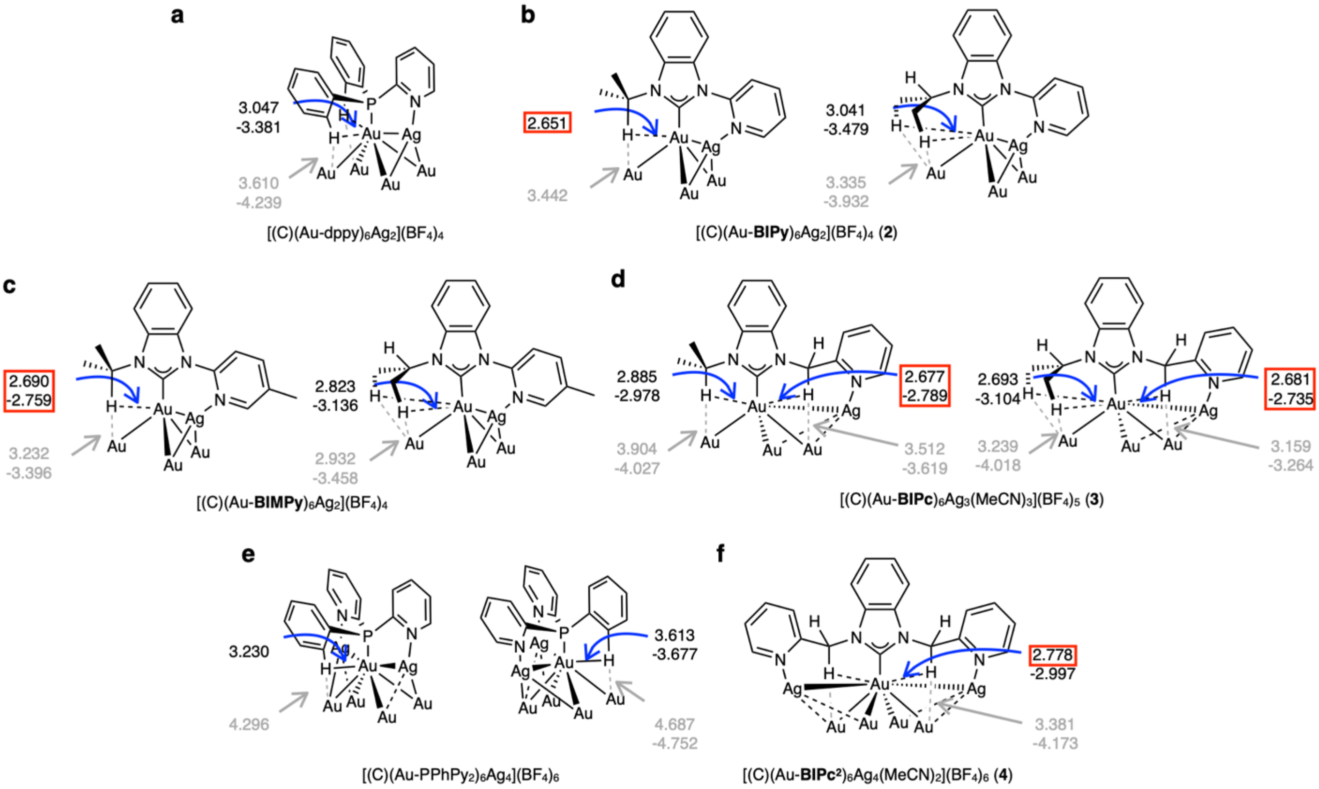

Figure S37. Schematic illustration of intramolecular Au···H−C bonds in **2**–**4** and some reported $CAu^I_6Ag^I_n$ clusters

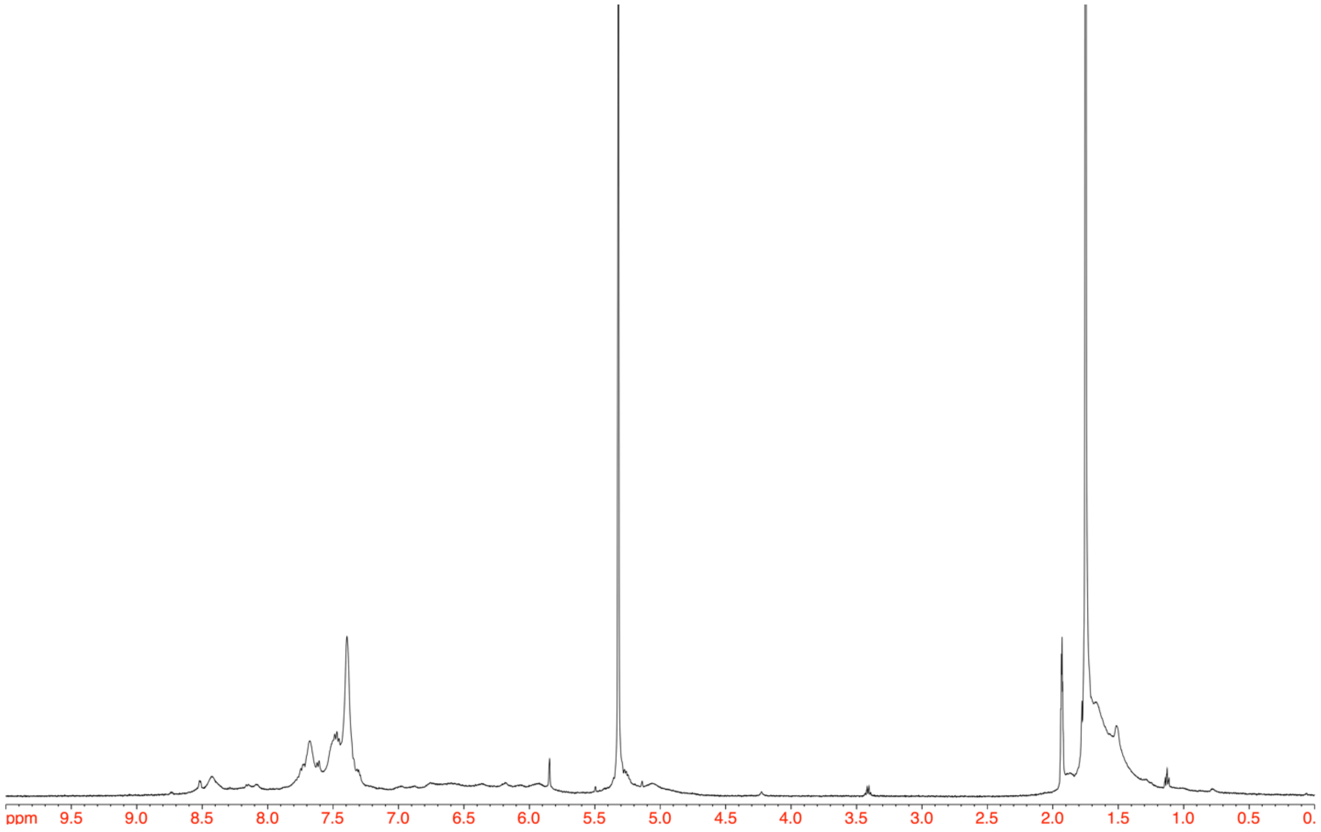

Figure S38. ^1H NMR spectrum of **3** (500 MHz, CD_2Cl_2/CD_3CN (9:1, v:v), 300 K)

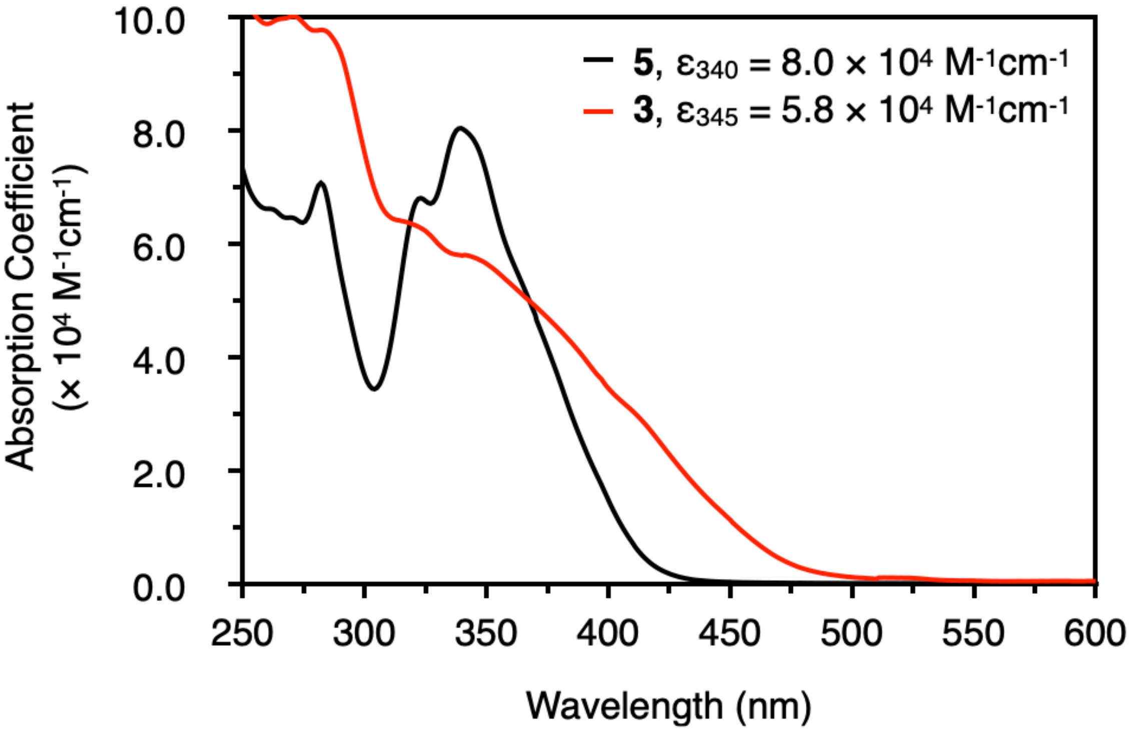

Figure S39. UV-vis absorption spectrum of **3** in CH_2Cl_2 (with **5** as a reference)

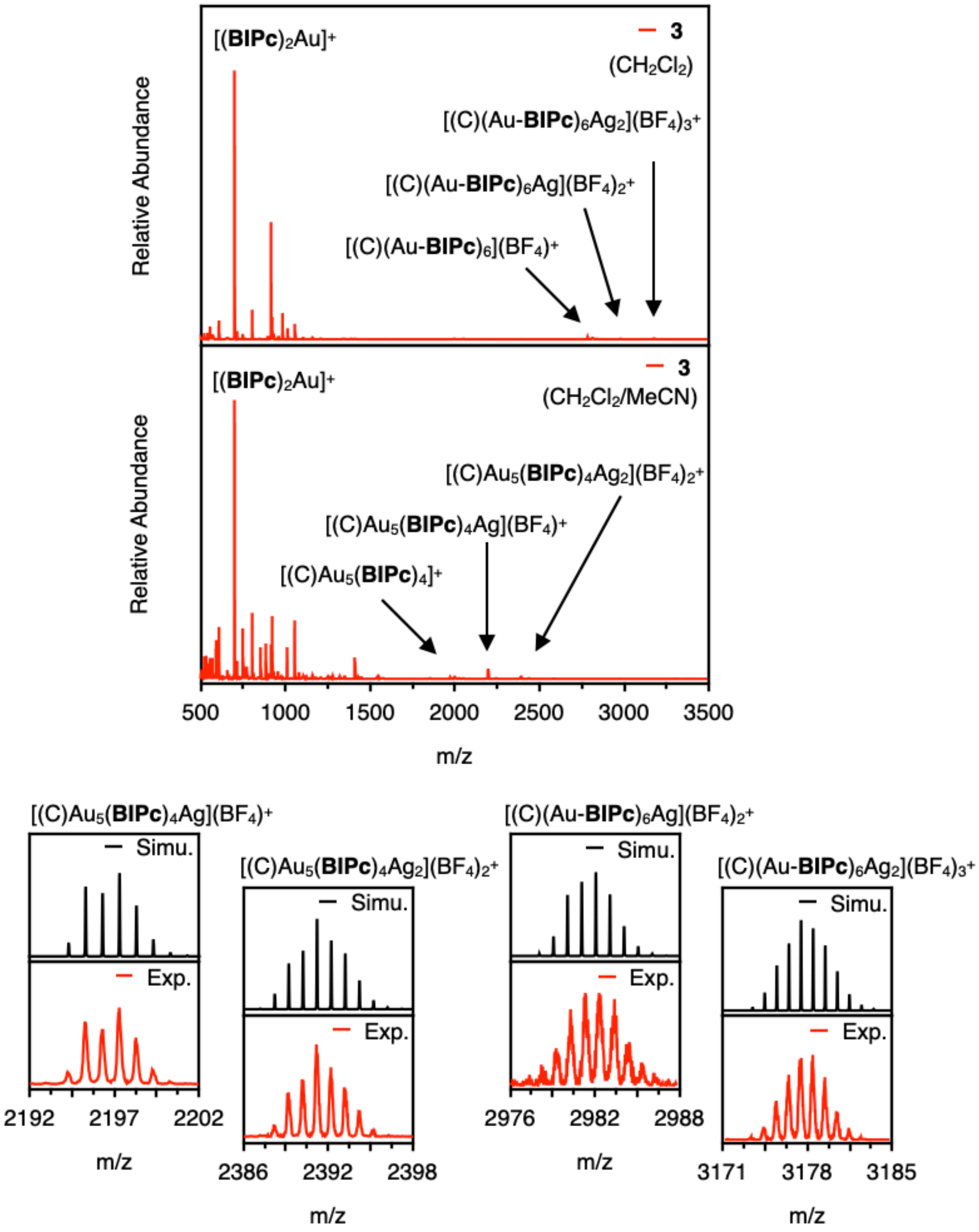

Figure S40. ESI MS spectra of **3**

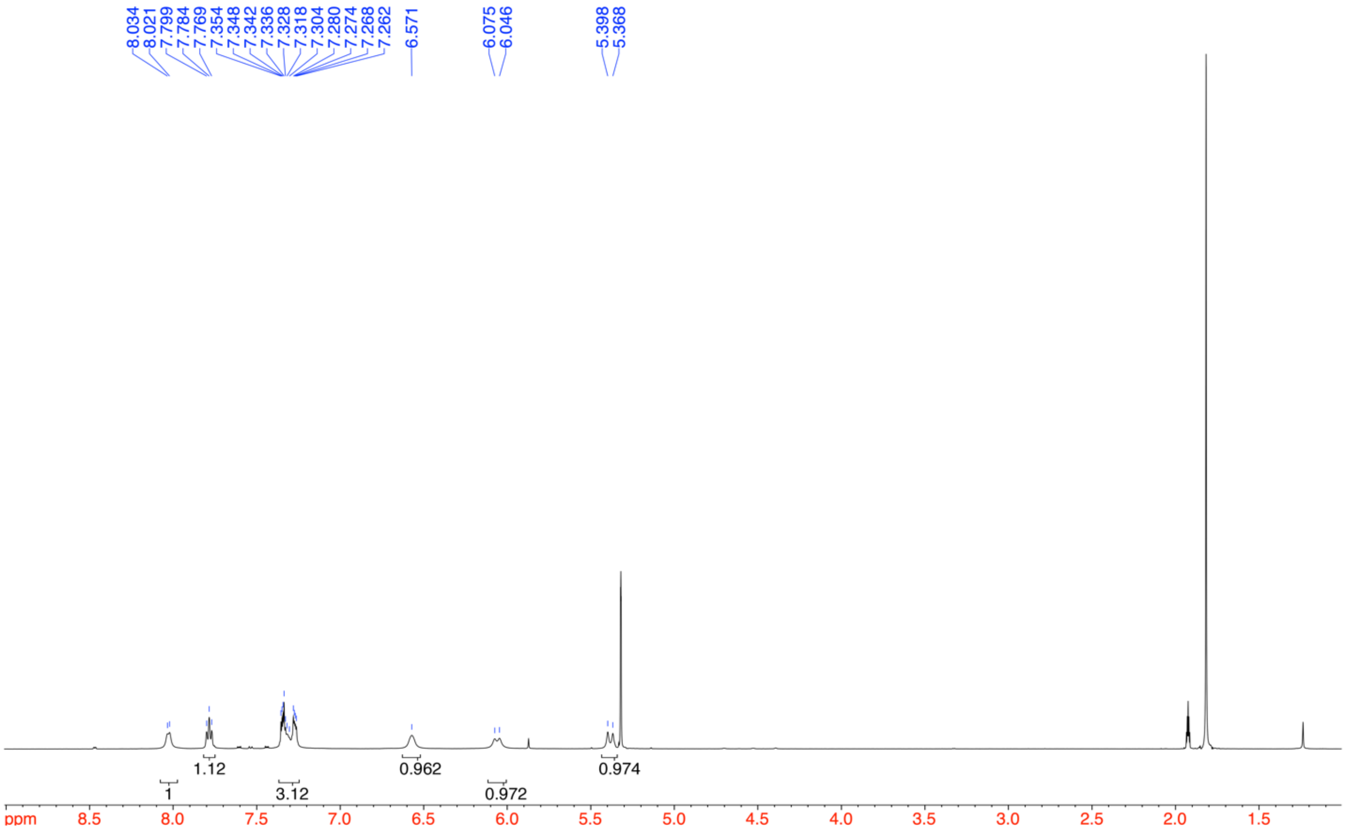

Figure S41. ^{1}H NMR spectrum of **4** (500 MHz, CD_2Cl_2/CD_3CN (9:1, v:v), 300 K)

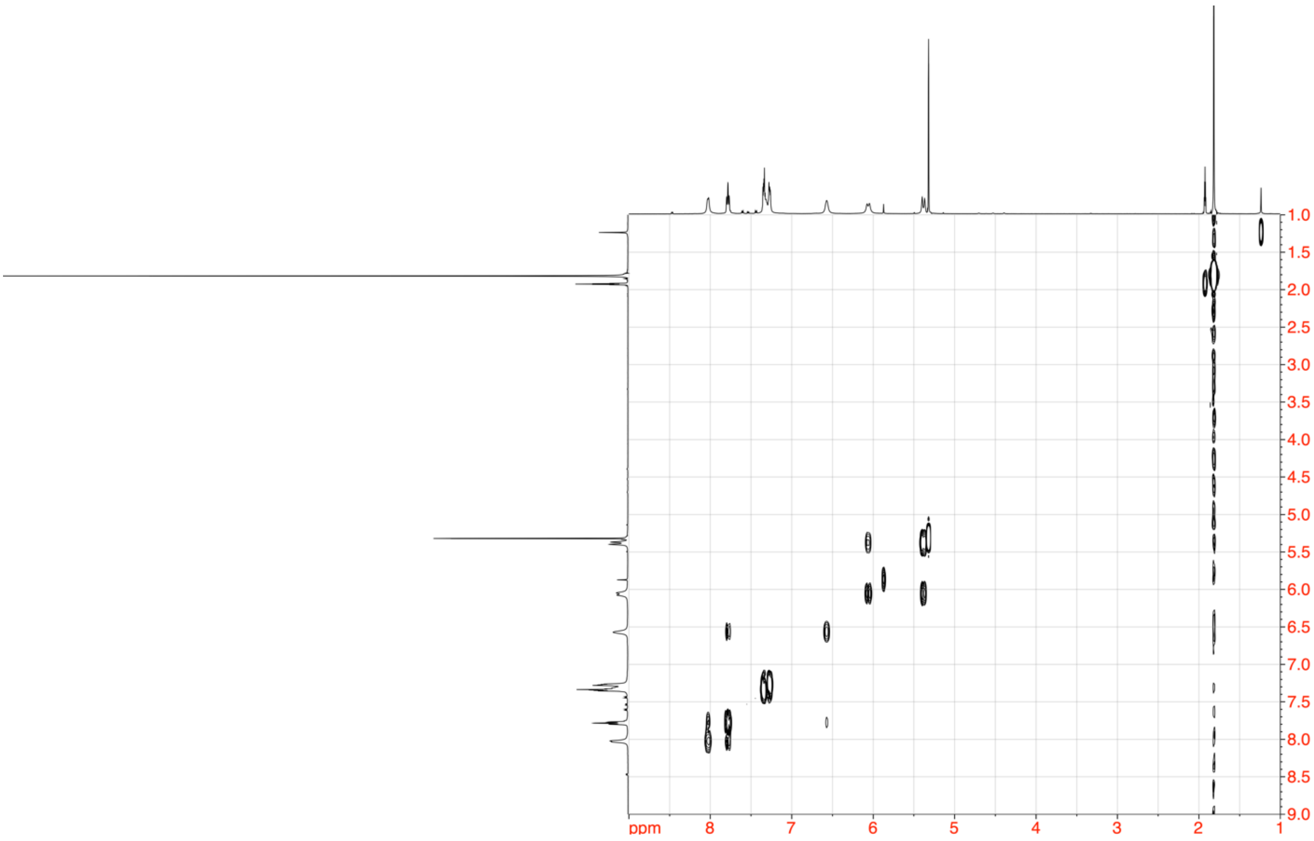

Figure S42. ^{1}H-^{1}H COSY NMR spectrum of **4** (500 MHz, CD_2Cl_2/CD_3CN (9:1, v:v), 300 K)

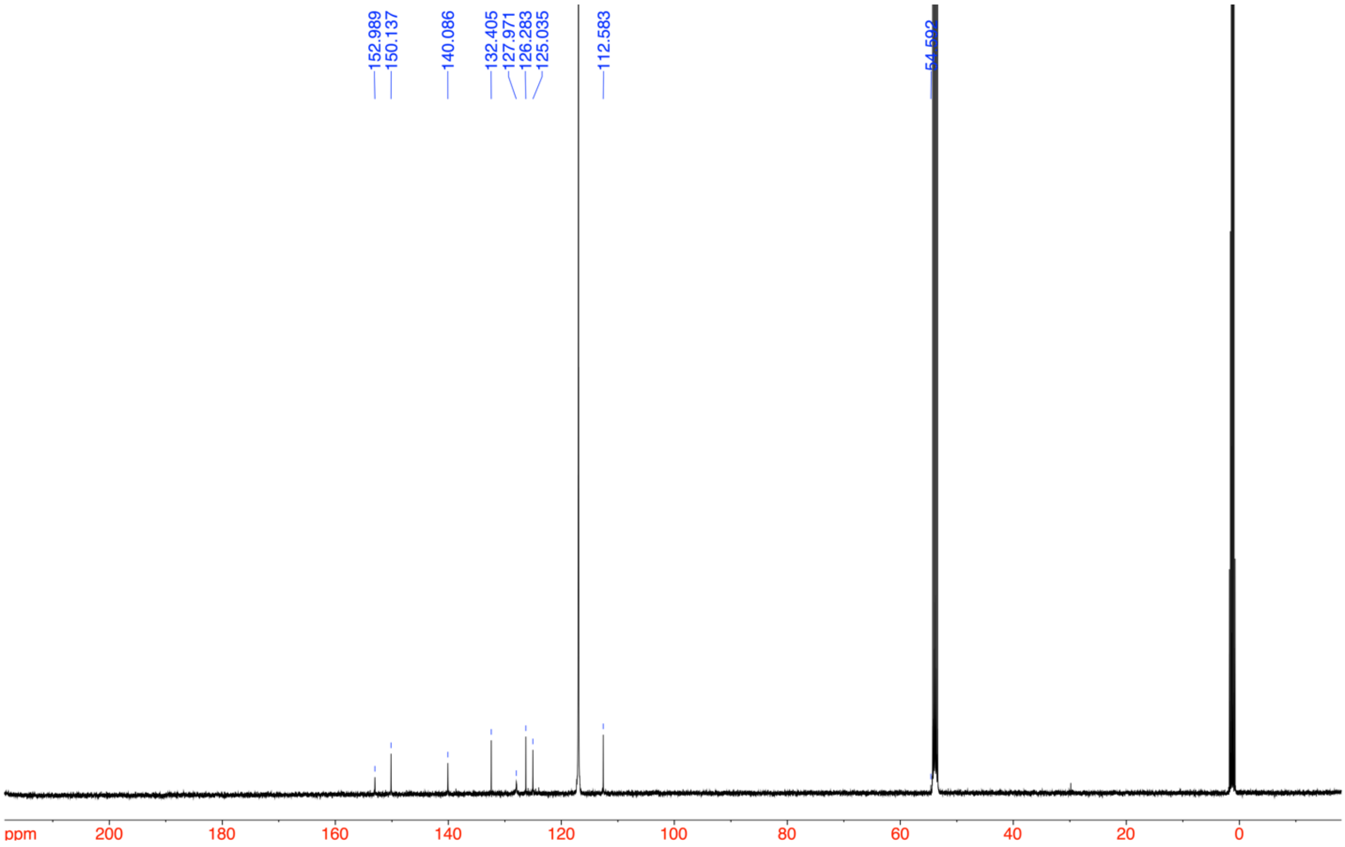

Figure S43. ^{13}C NMR spectrum of **4** (126 MHz, CD_2Cl_2/CD_3CN (9:1, v:v), 300 K)

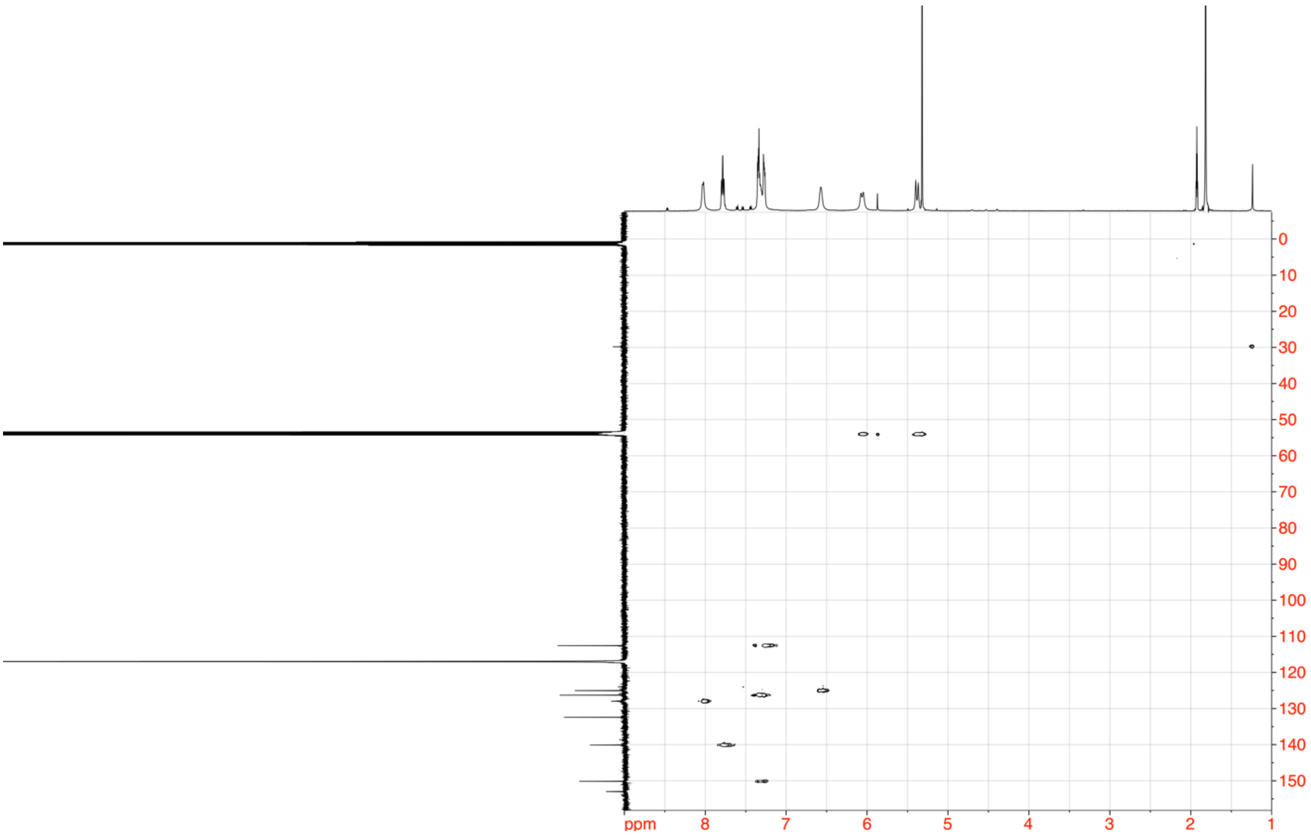

Figure S44. ^{1}H-^{13}C HSQC spectrum of **4** (500 MHz, CD_2Cl_2/CD_3CN (9:1, v:v), 300 K)

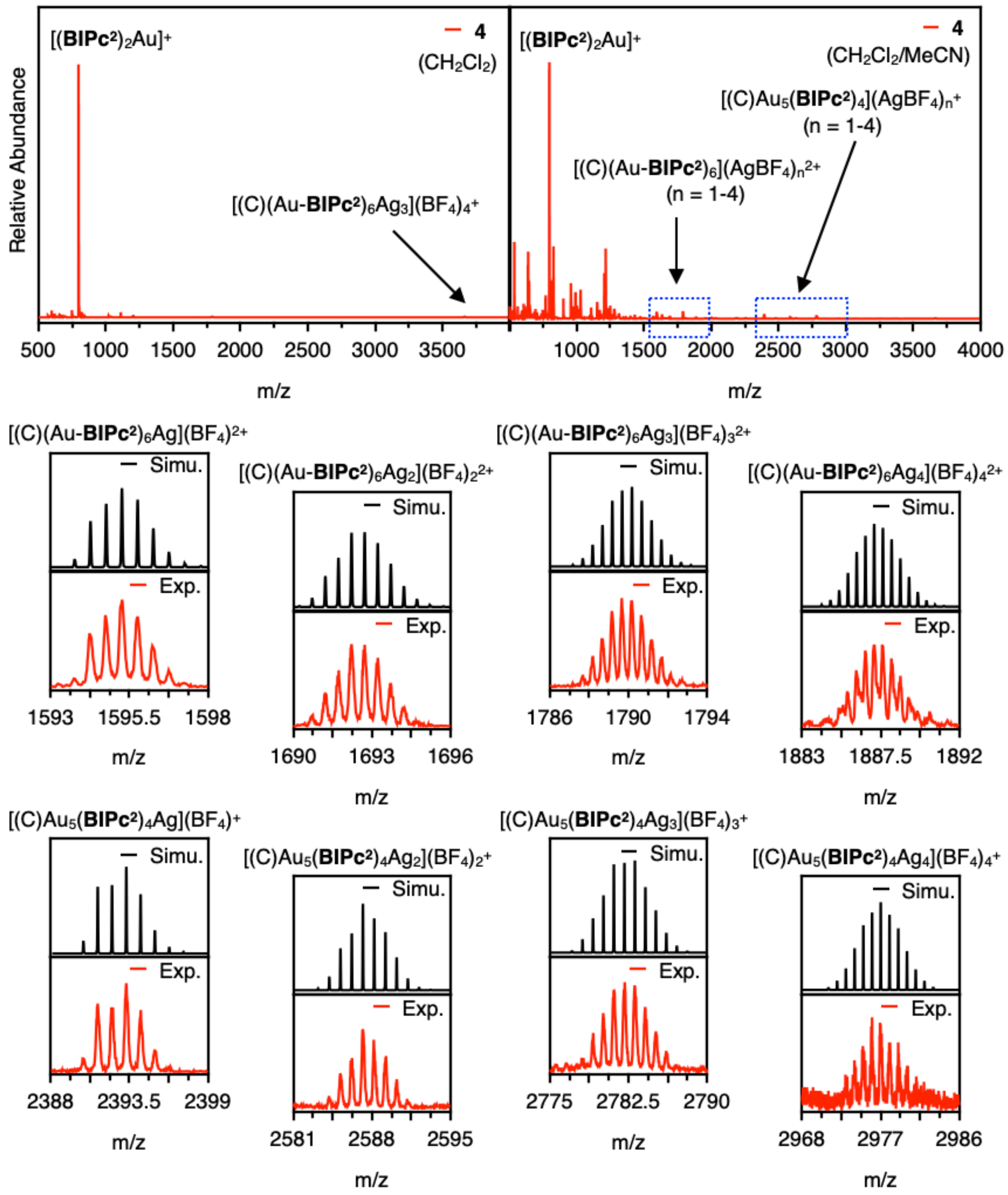

Figure S45. ESI MS spectra of **4**

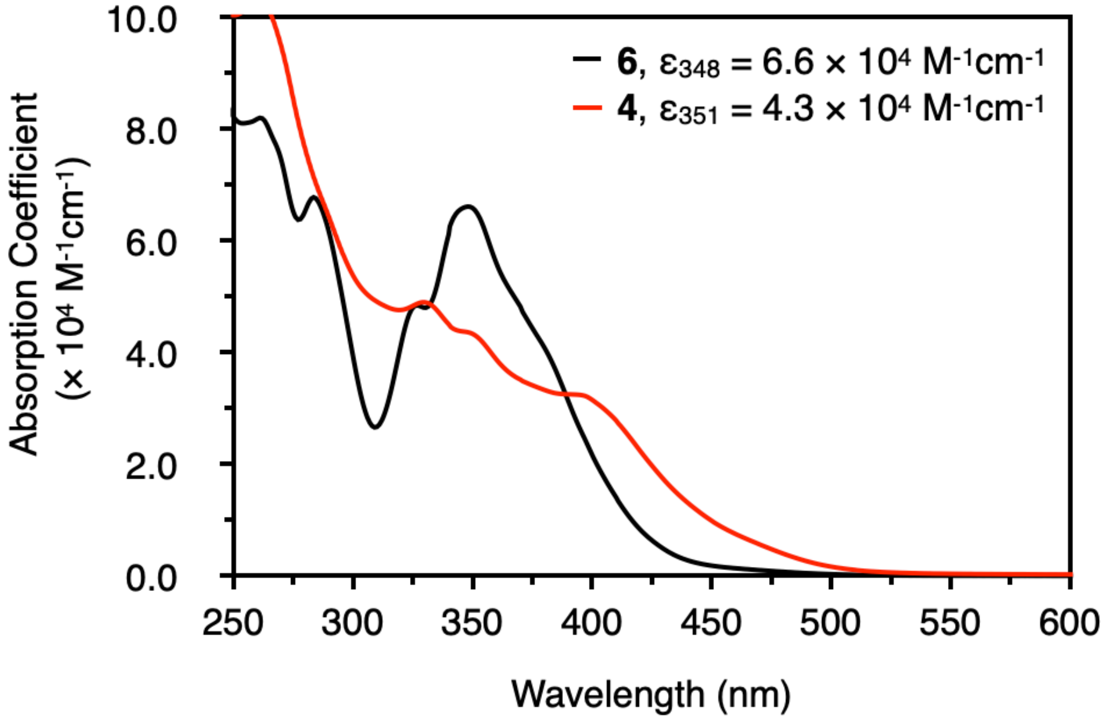

Figure S46. UV-vis absorption spectrum of **4** in CH_2Cl_2 (with **6** as a reference)

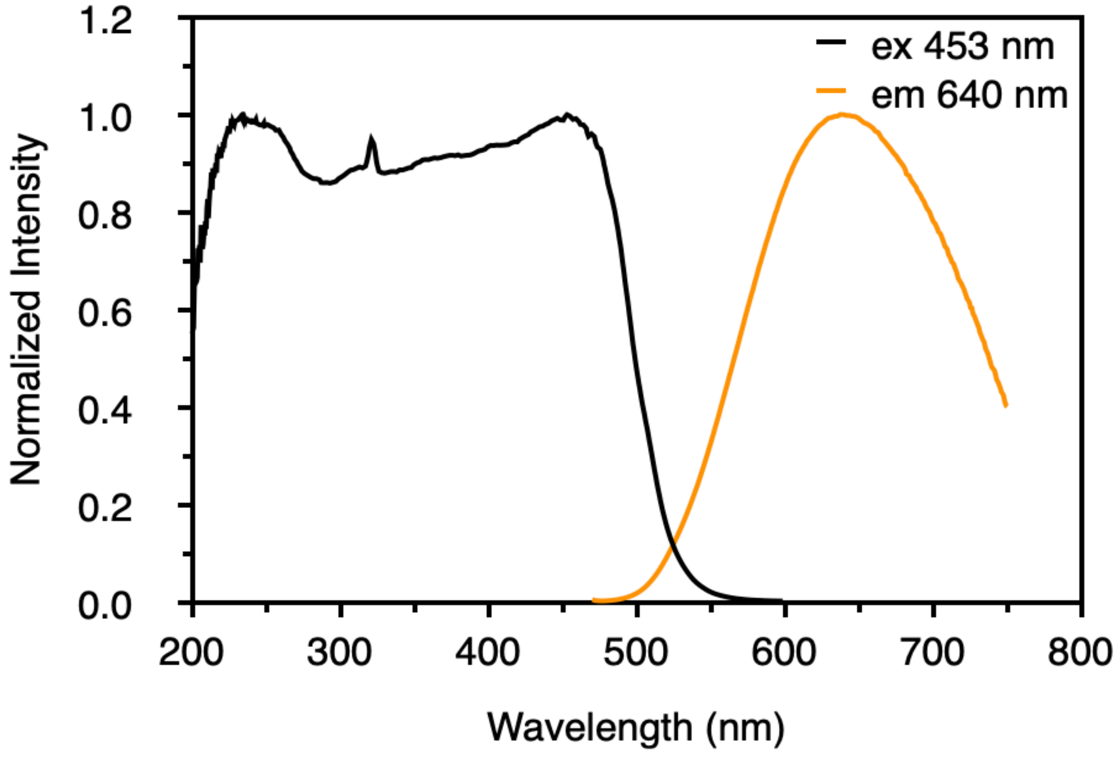

Figure S47. Excitation and emission spectra of **3** in the solid state

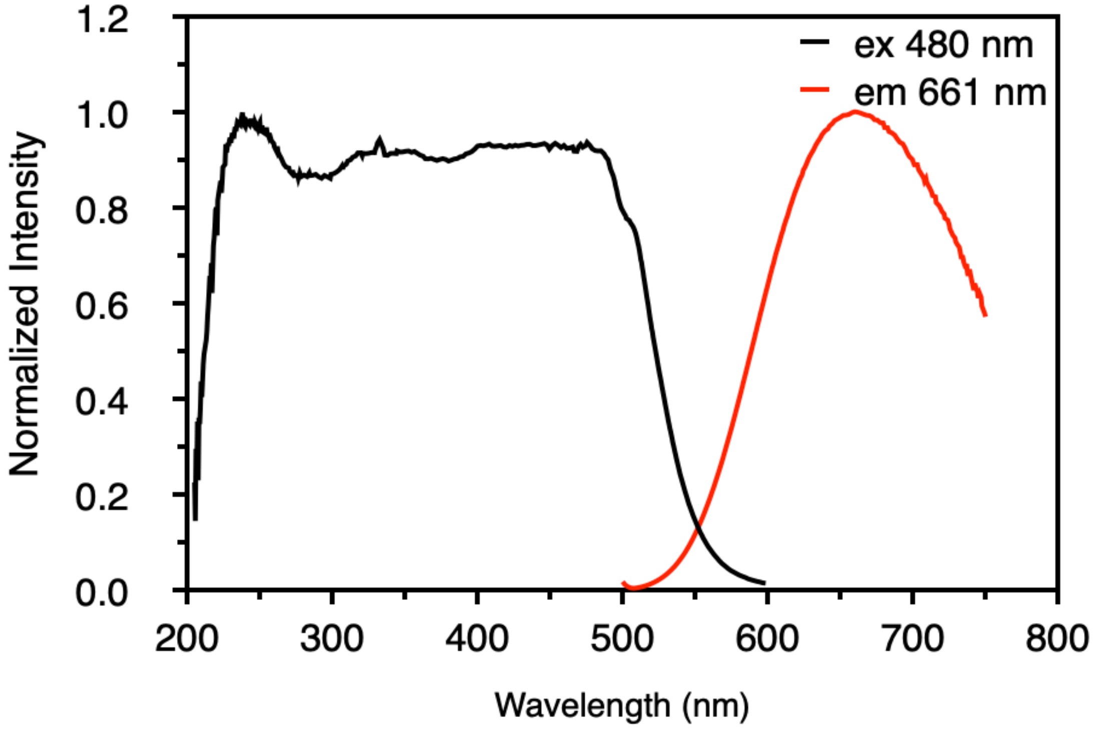

Figure S48. Excitation and emission spectra of **4** in the solid state

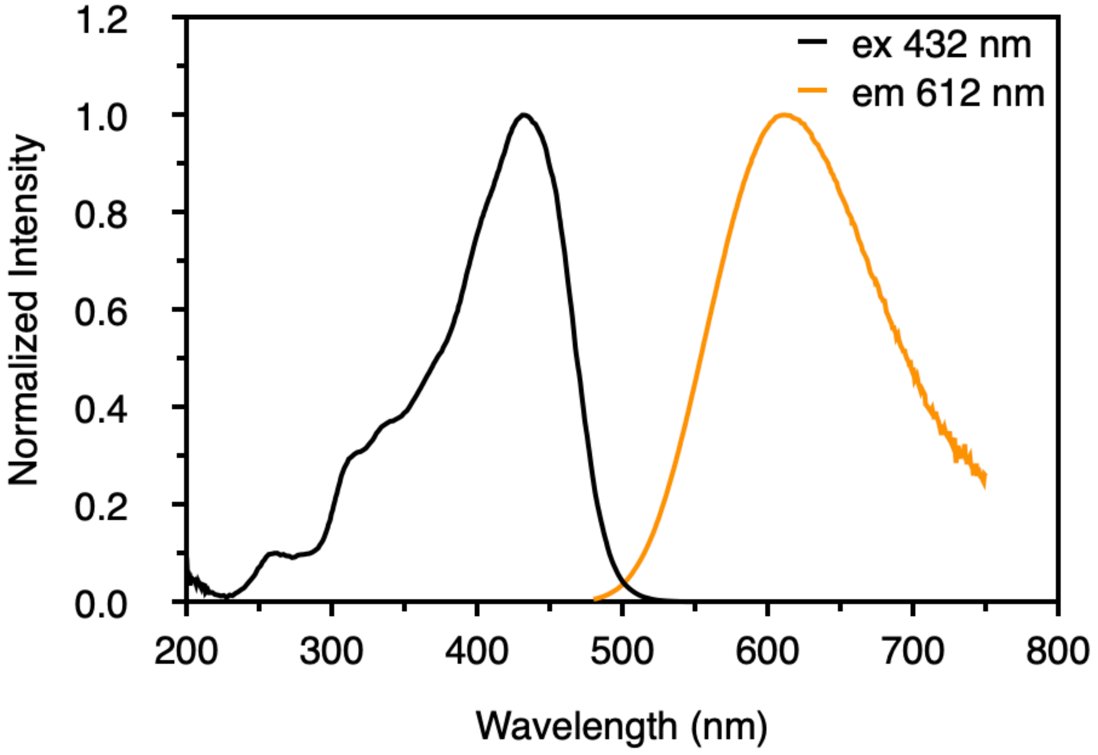

Figure S49. Excitation and emission spectra of **3** in CH_2Cl_2

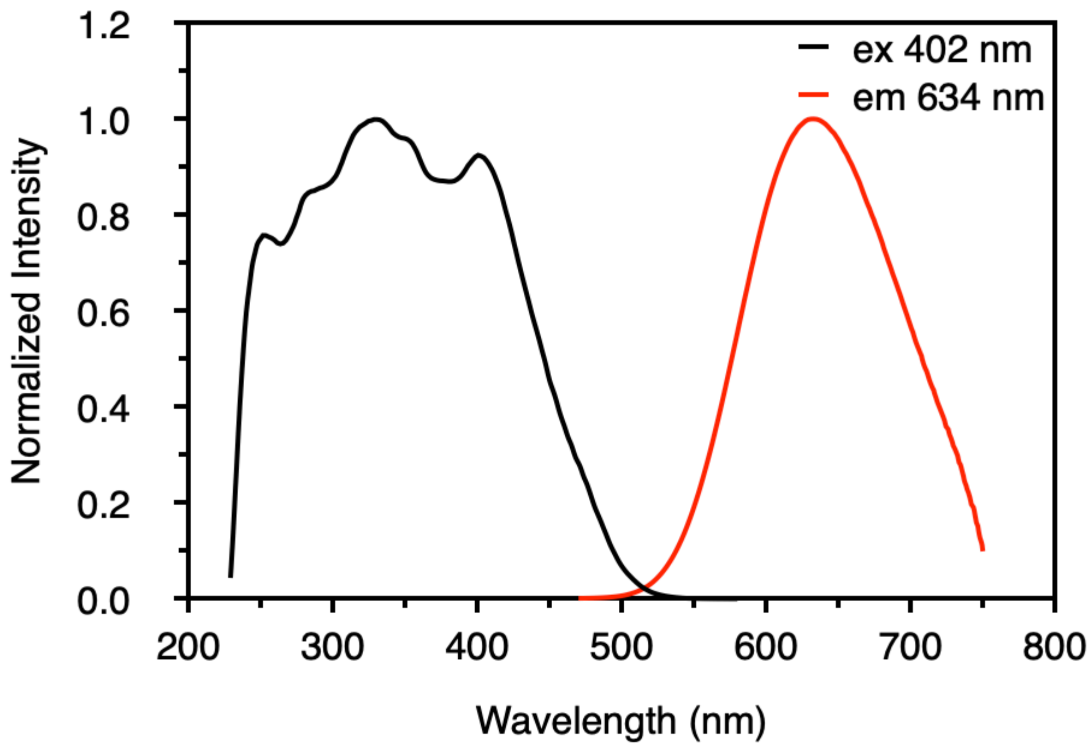

Figure S50. Excitation and emission spectra of **4** in CH_2Cl_2

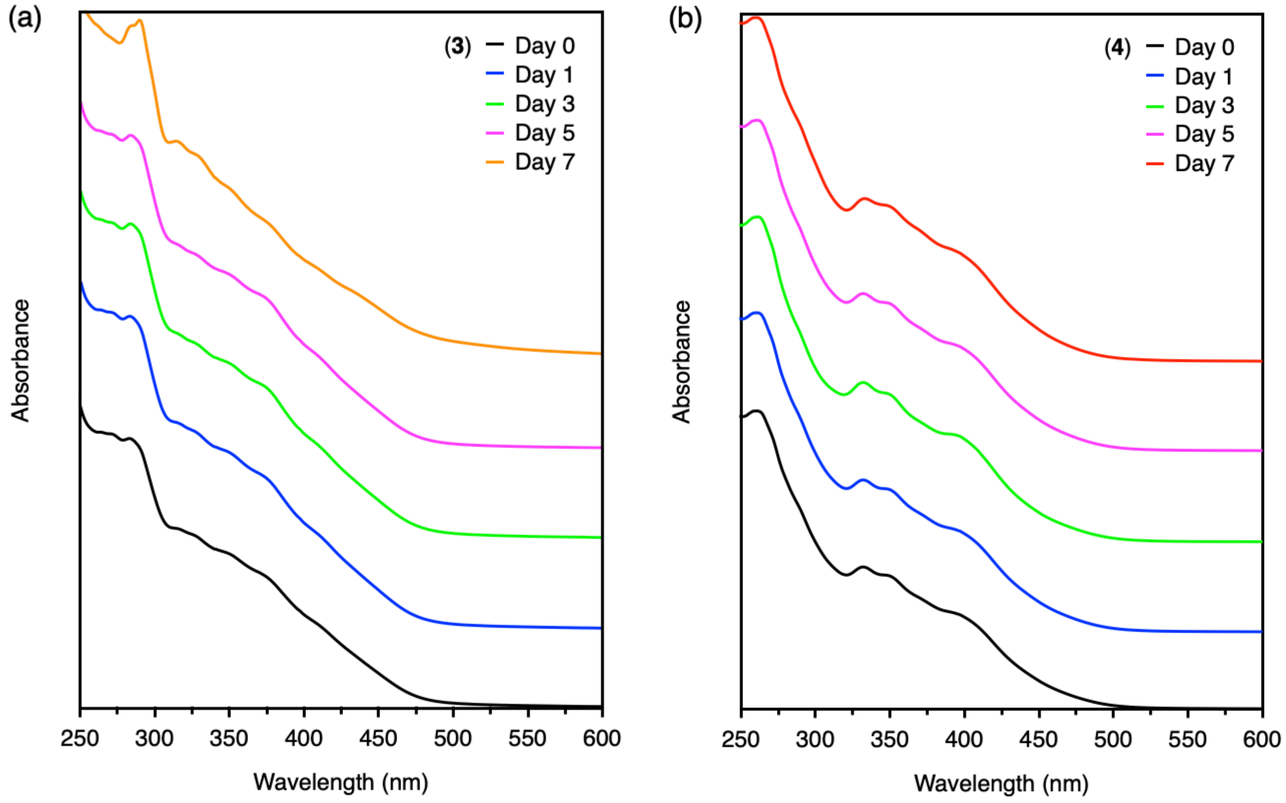

Figure S51. UV-vis absorption spectra of **3** (a) and **4** (b) in CH_2Cl_2 that are kept under daylight at room temperature for one week to check their stability. ($c = 3.3 \times 10^{-5}$ mol/L)

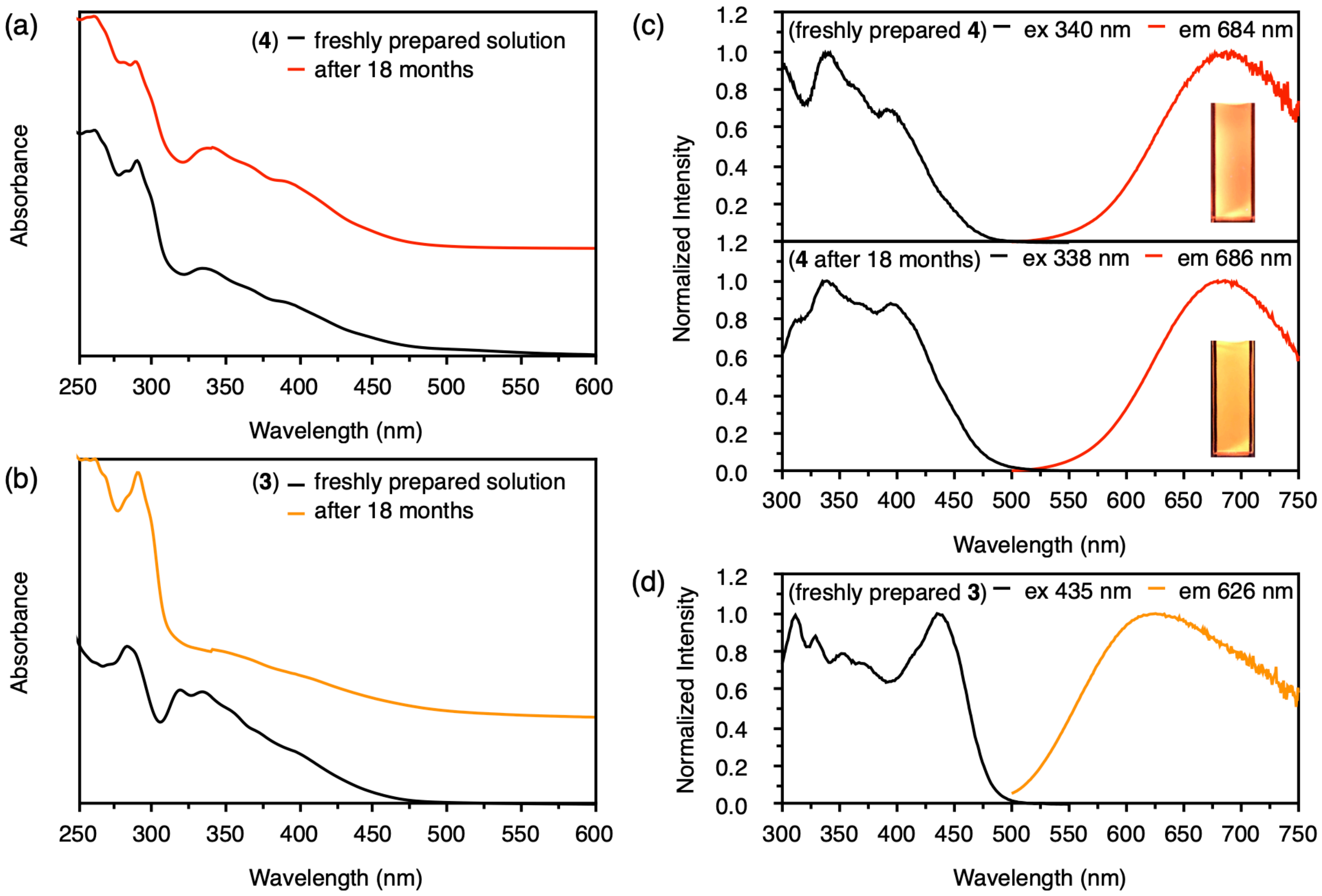

Figure S52. (a, b) UV-vis absorption spectra of **4** and **3** in CH_2Cl_2/CH_3CN (9:1, v:v, $c = 3.3 \times 10^{-5}$ mol/L) before and after stored at 4°C for 18 months. (c) Excitation and emission spectra of **4** in CH_2Cl_2/CH_3CN (9:1, v:v, $c = 3.3 \times 10^{-5}$ mol/L) before and after stored at 4 °C for 18 months. Insets: photos of luminescence under 365 nm excitation. (d) Excitation and emission spectra of freshly prepared **3** in CH_2Cl_2/CH_3CN (9:1, v:v, $c = 3.3 \times 10^{-5}$ mol/L). QYs of freshly prepared **4** and **3** in CH_2Cl_2/CH_3CN (9:1, v:v) are 0.09 and 0.01, respectively.

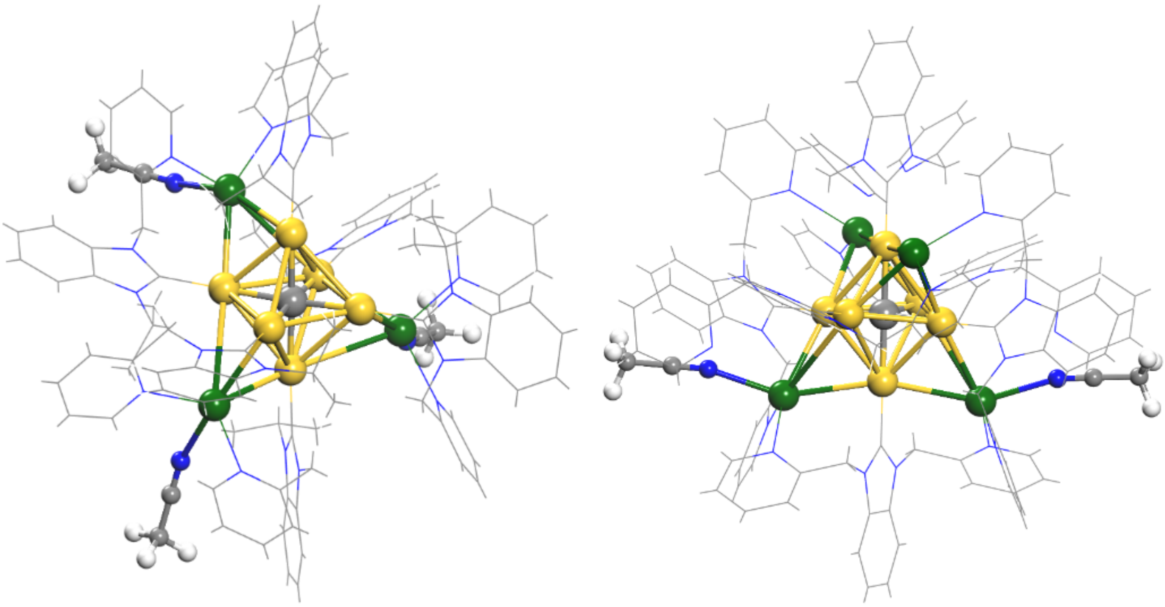

Figure S53. Optimized structures of **3** and **4**

Table S5. Bader charges and natural population analysis (NPA) charges of the central C, Au and Ag atoms in **2**-**6**

	Bader charge	NPA charge
	[(C)(Au^{I}-BIPy)$_6$Ag$^{I}_2$](BF$_4$)$_4$ (**2**)	
C	**–0.992**	**–1.673**
Au	0.198/0.206/0.202<br>0.201/0.198/0.203 **(avg.: 0.201)**	0.246/0.232/0.229<br>0.231/0.239/0.227 **(avg.: 0.234)**
Ag	0.575/0.574 **(avg.: 0.575)**	0.567/0.569 **(avg.: 0.568)**
	[(C)(Au^{I}-BIPc)$_6$Ag$^{I}_3$(CH$_3$CN)$_3$](BF$_4$)$_5$ (**3**)	
C	**–0.993**	**–1.640**
Au	0.177/0.186/0.181<br>0.180/0.176/0.184 **(avg.: 0.181)**	0.169/0.183/0.183<br>0.170/0.170/0.183 **(avg.: 0.176)**
Ag	0.596/0.594/0.595 **(avg.: 0.595)**	0.657/0.658/0.657 **(avg.: 0.657)**
	[(C)(Au^{I}-BIPc^{2})$_6$Ag$^{I}_4$(CH$_3$CN)$_2$](BF$_4$)$_6$ (**4**)	
C	**–0.946**	**–1.614**
Au	0.163/0.170/0.185<br>0.170/0.188/0.178 **(avg.: 0.176)**	0.129/0.104/0.236<br>0.104/0.236/0.075 **(avg.: 0.147)**
Ag	0.609/0.609/0.580/0.581 **(avg.: 0.595)**	0.697/0.697/0.645/0.645 **(avg.: 0.671)**
	[(C)(Au^{I}-BIPc)$_6$](BF$_4$)$_2$ (**5**)	
C	**–1.019**	**–1.721**
Au	0.188/0.190/0.187<br>0.188/0.190/0.187 **(avg.: 0.188)**	0.256/0.235/0.251<br>0.256/0.235/0.251 **(avg.: 0.247)**
	[(C)(Au^{I}-BIPc^{2})$_6$](BF$_4$)$_2$ (**6**)	
C	**–1.028**	**–1.728**
Au	0.189/0.190/0.188<br>0.193/0.194/0.185 **(avg.: 0.190)**	0.225/0.255/0.244<br>0.227/0.261/0.260 **(avg.: 0.245)**

Table S6. Excited states of **3** with oscillator strength (*f*) larger than 0.02

State number	λ (nm)	ΔE (eV)	*f*	Transition character
1	403	3.075	0.1244	H–2 → L
4	387	3.205	0.0311	H–2 → L+1, H–1 → L
5	377	3.287	0.3554	H–2 → L+1, H–1 → L, H → L+1
6	377	3.288	0.3199	H–1 → L+1
7	358	3.460	0.1465	H–2 → L
8	346	3.588	0.2278	H–1 → L+2
9	345	3.589	0.2127	H–2 → L+2, H → L+2
10	339	3.654	0.0477	H–4 → L+2
11	318	3.894	0.0559	H–4 → L+1, H–3 → L
12	315	3.933	0.0400	H–4 → L, H–3 → L+1
13	315	3.934	0.0395	H–4 → L+1, H–3 → L
24	300	4.138	0.0322	H–1 → L+6
25	299	4.142	0.0358	H–2 → L+6
26	299	4.144	0.0266	H–1 → L+6
31	298	4.162	0.0429	H–5 → L+1
33	296	4.190	0.0209	H–7 → L, H–6 → L+1
34	296	4.190	0.0251	H–7 → L+1
35	295	4.205	0.0240	H–1 → L+8
36	295	4.206	0.0253	H–2 → L+8
41	292	4.245	0.0826	H → L+9
43	292	4.252	0.0500	H–1 → L+9
44	291	4.254	0.0351	H–2 → L+9
45	291	4.254	0.0208	H–9 → L
49	291	4.264	0.0210	H–10 → L+1
53	289	4.286	0.0833	H–14 → L
54	289	4.291	0.0808	H–14 → L+1
58	285	4.349	0.0914	H–2 → L+10
59	285	4.353	0.0307	H → L+10
60	285	4.355	0.0319	H → L+11
67	282	4.401	0.0435	H–16 → L, H–15→ L+1
70	278	4.460	0.0288	H–18 → L
71	278	4.465	0.0286	H–17 → L
72	277	4.470	0.0253	H–19 → L+1
73	276	4.494	0.0687	H–5 → L+2, H–3 → L+2
74	276	4.497	0.0654	H–6 → L+2, H–4 → L+2
76	273	4.541	0.0248	H–8 → L+2
78	272	4.555	0.0305	H–9 → L+2
79	272	4.557	0.0362	H–10 → L+2
80	272	4.566	0.0293	H–2 → L+12
81	271	4.567	0.0339	H–1 → L+12
83	270	4.585	0.0380	H–1 → L+12
84	270	4.587	0.0342	H–2 → L+12
93	268	4.619	0.0295	H–1 → L+13, H → L+14
94	268	4.624	0.0278	H–2 → L+14, H–1 → L+13
96	268	4.626	0.0244	H–20 → L+1
100	276	4.956	0.0285	H–15 → L+2

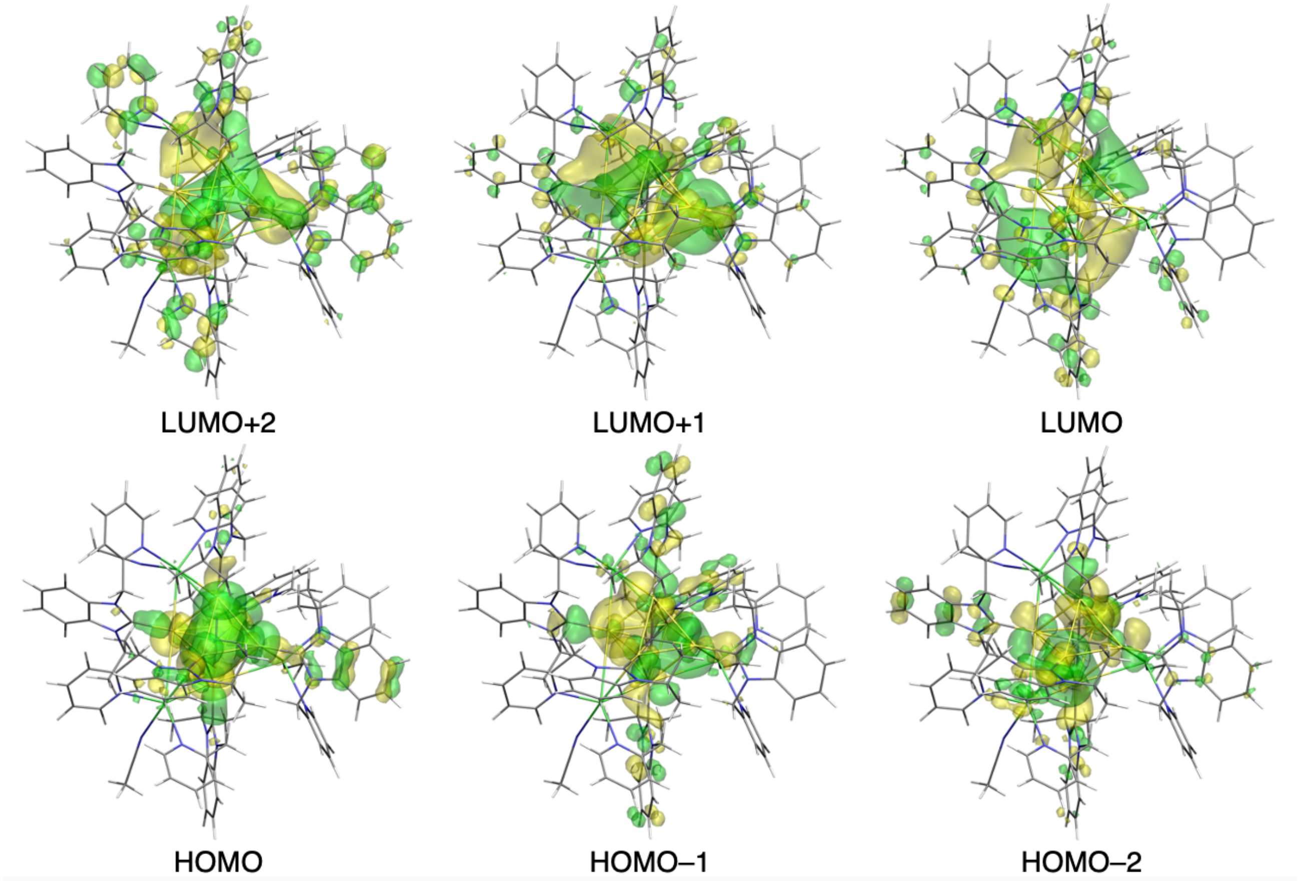

Figure S54. Involved molecular orbitals of **3**

Table S7. Excited states of **4** with oscillator strength (*f*) larger than 0.02

State number	λ (nm)	ΔE (eV)	*f*	Transition character
2	416	2.980	0.3009	H–2 → L
3	415	2.986	0.1329	H–1 → L
5	361	3.433	0.1614	H–1 → L+1, H → L+1
6	361	3.436	0.0812	H–1 → L+1, H → L+1
7	359	3.453	0.1491	H–2 → L+1, H–1 → L+2
8	359	3.457	0.1486	H → L+2
9	355	3.490	0.0618	H–2 → L+2
10	352	3.520	0.2487	H–5 → L, H–2 → L+1, H–1 → L+2
11	345	3.597	0.2150	H–5 → L
13	327	3.796	0.1045	H–6 → L
15	320	3.877	0.0299	H–7 → L
17	318	3.898	0.1237	H–10 → L, H–6 → L+1
22	313	3.955	0.0250	H–13 → L, H–3 → L+2
24	311	3.989	0.0558	H–16 → L, H–3 → L+1
29	301	4.123	0.1062	H–20 → L, H–19 → L
33	297	4.170	0.0254	H–23 → L
37	296	4.192	0.0477	H–1 → L+6, H → L+3
42	294	4.220	0.0265	H–2 → L+6
51	291	4.261	0.0261	H–1 → L+7

66	287	4.324	0.0231	H–7 → L+1
71	285	4.344	0.0379	H–9 → L+1, H–2 → L+11
78	282	4.404	0.0386	H–12 → L+1
79	282	4.404	0.0568	H–12 → L+1, H–1 → L+13, H → L+15
81	281	4.415	0.0281	H–11 → L+2
82	280	4.421	0.0245	H–1 → L+13, H → L+15
83	280	4.424	0.0369	H–2 → L+13
85	279	4.441	0.0698	H–14 → L+1
87	278	4.448	0.0563	H–13 → L+2
90	278	4.458	0.0255	H–13 → L+2
91	278	4.460	0.0333	H–2 → L+14
98	276	4.485	0.0370	H–12 → L+2

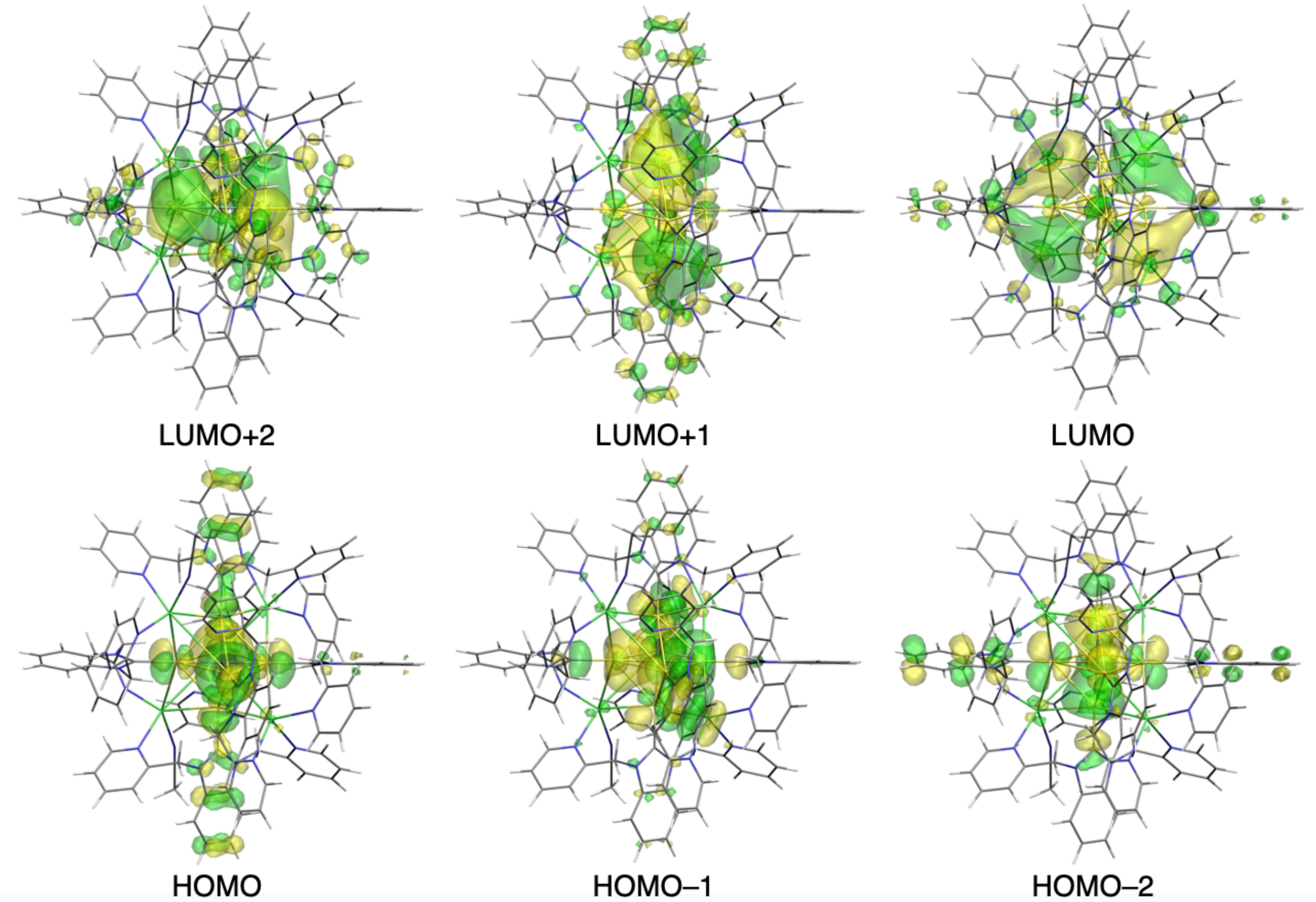

Figure S55. Involved molecular orbitals of **4**

Table S8. Orbital composition analysis with Mulliken partition of **2**-**6**

	LUMO+2	LUMO+1	LUMO	HOMO	HOMO–1	HOMO–2
			$[(C)(Au^{I}\text{-BIPy})_6Ag^{I}_2](BF_4)_4$ (**2**)			
C	0.04%	0.02%	0.25%	20.83%	22.15%	21.96%
Au^{I}_6	10.57%	12.75%	33.21%	40.18%	51.11%	50.34%
Ag^{I}_2	4.16%	4.51%	31.30%	3.09%	1.14%	1.02%
$(BIPy)_6$	85.24%	82.71%	35.23%	35.90%	25.60%	26.68%
			$[(C)(Au^{I}\text{-BIPc})_6Ag^{I}_3(CH_3CN)_3](BF_4)_5$ (**3**)			
C	0.41%	1.59%	1.61%	24.71%	23.59%	23.60%
Au^{I}_6	29.86%	34.72%	34.66%	53.74%	44.82%	44.81%
Ag^{I}_3	18.32%	27.67%	28.02%	0.75%	3.55%	3.54%
$(BIPc)_6+(CH_3CN)_3$	51.41%	36.03%	35.70%	20.80%	28.04%	28.05%
			$[(C)(Au^{I}\text{-BIPc}^2)_6Ag^{I}_4(CH_3CN)_2](BF_4)_6$ (**4**)			
C	0.91%	1.14%	1.24%	24.66%	21.18%	22.76%
Au^{I}_6	27.81%	35.03%	26.19%	45.59%	43.62%	44.82%
Ag^{I}_4	29.89%	22.81%	44.93%	2.80%	2.77%	4.32%
$(BIPc^2)_6+(CH_3CN)_2$	41.40%	41.02%	27.64%	26.96%	32.44%	28.09%
			$[(C)(Au^{I}\text{-BIPc})_6](BF_4)_2$ (**5**)			
C	0.04%	0.04%	0.04%	26.76%	27.11%	25.17%
Au^{I}_6	30.75%	35.71%	38.85%	52.21%	52.95%	59.06%
$(BIPc)_6$	69.22%	64.25%	61.11%	21.03%	19.93%	15.78%
			$[(C)(Au^{I}\text{-BIPc}^2)_6](BF_4)_2$ (**6**)			
C	0.05%	0.08%	0.09%	27.02%	25.85%	26.11%
Au^{I}_6	35.04%	35.74%	39.42%	51.70%	53.37%	55.88%
$(BIPc^2)_6$	64.91%	64.18%	60.50%	21.28%	20.78%	18.01%

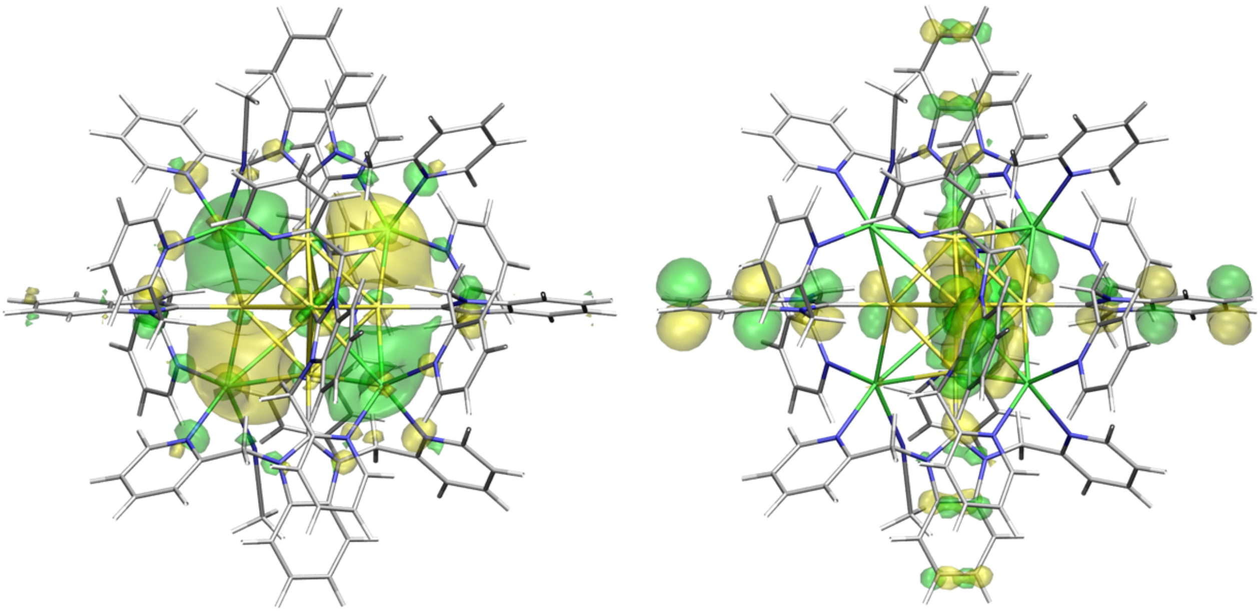

Figure S56. SOMO and SOMO-1 orbitals of **4**

Table S9. Phosphorescence energies of **2**, **3**, **4** and **4*** by the DFT calculations with the non-equilibrium scheme of IEF-PCM (CH_2Cl_2)

compound	Exp.	Phosphorescence energy	
	nm	eV	nm
**2**	562	2.09	592
**3**	612	1.94	642
[a]**4**	634	1.97	629
[a]**4***	-	1.82	681

([a]The optimized triplet structures of **4** and **4*** have an imaginary frequency at -36.95 and -33.87 nm, respectively.)

Table S10. Key structural parameters of **4***

Au–C (central C^{4-}, Å)	Au–Au (Å)	Au–C (NHC ligands, Å)*	Au–Ag (Å)	Ag–N (Å)
2.193	3.101	2.067	3.117	2.580

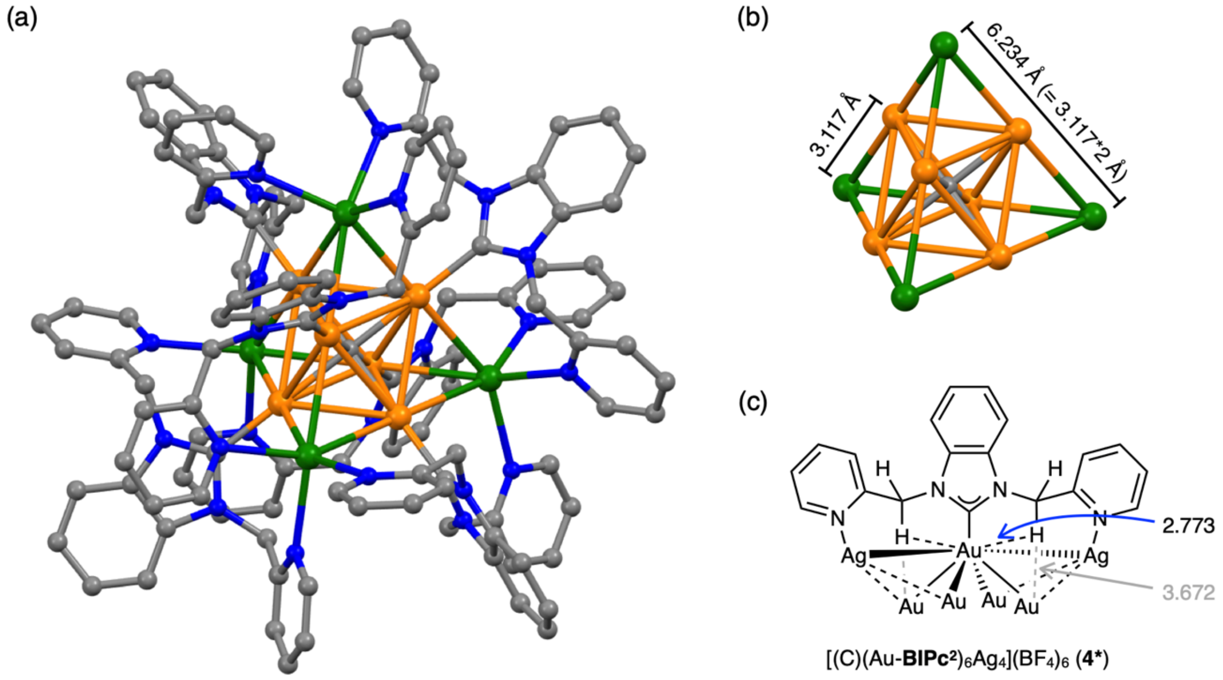

Figure S57. (a) Molecular structure of simulated [(C)(Au-**BIPc**^{2})$_6$Ag$_4$](BF$_4$)$_6$ (**4***). (b) Structure of a tetrahedral CAu_6Ag_4 core. (c) Schematic illustration of intramolecular Au···H–C bonds in **4***.

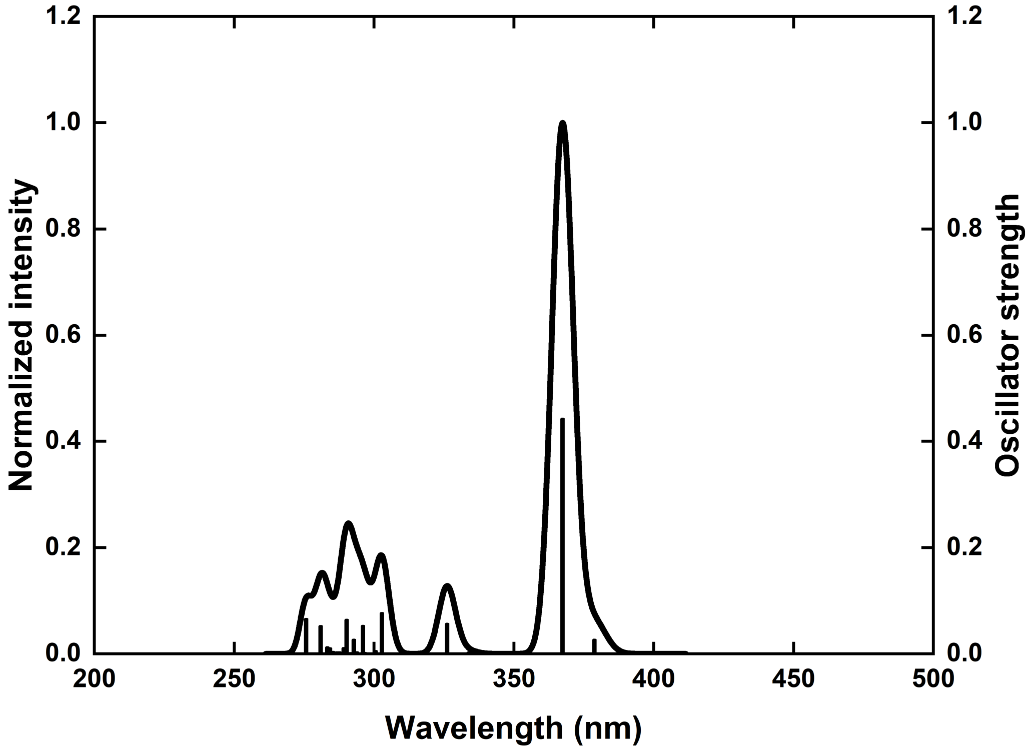

Figure S58. Calculated UV-vis absorption spectrum of cluster **4*** in CH_2Cl_2.

Table S11. Calculated radiative rate constants of the low-lying spin-orbit states of **2**, **3** and **4*** by ZORA (B3LYP, DZ) and their main components.

	Expt.			Theory					
	Φ	τ (μs)	k_r (10^5 s^{-1})	St.	ΔE	*f*	τ (μs)	k_r (10^5 s^{-1})	Main component
**2**	0.86	1.66	5.18	1	2.111	0.000050	102.80	0.097	T_1(Sz = 0)
				2	2.111	0.000050	103.30	0.097	T_1(Sz = ±1)
				3	2.114	0.000146	35.25	0.284	T_1(Sz = ±1)
				4	2.200	0.016000	0.30	33.591	S_1
				5	2.823	0.000324	8.93	1.119	T_2(Sz = ±1)
**3**	0.04	0.23	1.74	1	1.984	0.000010	595.80	0.017	T_1(Sz = ±1)
				2	1.984	0.000209	27.97	0.358	T_1(Sz = 0)
				3	1.986	0.000122	48.03	0.208	T_1(Sz = ±1)
				4	2.069	0.021660	0.25	40.258	S_1
				5	2.546	0.000040	88.99	0.112	T_2(Sz = ±1)
**4**	0.40	1.41	2.84	-					
**4***	-	-	-	1	1.835	0.000027	249.90	0.040	T_1
				2	1.835	0.000044	155.40	0.064	T_1
				3	1.835	0.000108	63.10	0.158	T_1
				4	1.924	0.044520	0.14	71.531	S_1
				5	2.237	0.000123	37.41	0.267	T_2

Table S12. Singlet and triplet components and spin-orbit coupling (SO) of the low-lying spin-orbit states of **2**, **3** and **4***

Complex	Triplet	E_T (eV)	SO(T_x/S_1) cm^{-1}	SO(T_x/S_3) cm^{-1}	Singlet	E_S (eV)	*f*
	T_1	2.120	43	401	S_1	2.208	0.01619
**2**	T_2	2.847	200	77	S_2	2.988	0.03152
					S_3	3.141	0.05004
	T_1	1.989	18	91	S_1	2.075	0.02205
**3**	T_2	2.548	416	36	S_2	2.646	0.02993
					S_3	2.852	0.08439
	T_1	1.833	65	47	S_1	1.922	0.04541
**4***	T_2	2.239	149	24	S_2	2.321	0.03578
					S_3	2.524	0.00061

10. References

1. Z. Lei, M. Endo, H. Ube, T. Shiraogawa, P. Zhao, K. Nagata, X.-L. Pei, T. Eguchi, T. Kamachi, M. Ehara, T. Ozawa, M. Shionoya, *Nat. Commun.*, 2022, **13**, 4288.
2. M. C. Jahnke, T. Pape, F. E. Hahn, *Eur. J. Inorg. Chem.*, 2009, 1960.
3. H. V. Huynh, V. T. Pham, N. T. T. Chi, *Eur. J. Inorg. Chem.*, 2017, 5650.
4. X. Zhang, S. Gu, Q. Xia, W. Chen, *J. Organomet. Chem.*, 2009, **694**, 2359.
5. H. Ube, Q. Zhang, M. Shionoya, *Organometallics*, 2018, **37**, 2007.
6. Z. Lei, K. Nagata, H. Ube, M. Shionoya, *J. Organomet. Chem.*, 2020, **917**, 121271.
7. Z. Lei, Q.-M. Wang, *Coord. Chem. Rev.*, 2019, **378**, 382.
8. J.-H. Jia, Q.-M. Wang, *J. Am. Chem. Soc.*, 2009, **131**, 16634.
9. Z. Lei, X.-L. Pei, Z.-J. Guan, Q.-M. Wang, *Angew. Chem. Int. Ed.*, 2017, **56**, 7117.
10. A. D. Becke, *J. Chem. Phys.*, 1993, **98**, 5648.
11. P. J. Hay, W. R. Wadt, *J. Chem. Phys.*, 1985, **82**, 270.
12. J. S. Binkley, J. A. Pople, W. J. Hehre, *J. Am. Chem. Soc.*, 1980, **102**, 939.
13. J. Tomasi, B. Mennucci, R. Cammi, *Chem. Rev.*, 2005, **105**, 2999.
14. M. J. Frisch et al, *Gaussian 16 rev. A03*, Wallingford, CT, 2016.
15. T. Lu, F. Chen, *J. Comput. Chem.*, 2012, **33**, 580.
16. F. Wang, T. Ziegler, *J. Chem. Phys.*, 2005, **123**, 154102.
17. G. te Velde, F. M. Bickelhaupt, E. J. Baerends, C. Fonseca Guerra, S. J. A. van Gisbergen, J. G. Snijders, T. Ziegler, *J. Comput. Chem.*, 2001, **22**, 931.
18. F. P. Gabbaï, A. Schier, J. Riede, H. Schmidbaur, *Chem. Ber.*, 1997, **130**, 111.
